# A heuristic method for solving the Steiner tree problem in graphs using network centralities

**DOI:** 10.1371/journal.pone.0303764

**Published:** 2024-06-06

**Authors:** Misa Fujita, Yutaka Shimada, Takayuki Kimura, Tohru Ikeguchi

**Affiliations:** 1 Department of Electrical and Electronic Engineering, School of Engineering, Chukyo University, Nagoya-shi, Aichi, Japan; 2 Department of Management Science, Graduate School of Engineering, Tokyo University of Science, Katsushika-ku, Tokyo, Japan; 3 Graduate School of Science and Engineering, Saitama University, Sakura-ku, Saitama, Japan; 4 Department of Electrical, Electronics and Communication Engineering, Faculty of Fundamental Engineering, Nippon Institute of Technology, Miyashiro-cho, Minami-saitama-gun, Saitama, Japan; 5 Department of Information and Computer Technology, Faculty of Engineering, Tokyo University of Science, Katsushika-ku, Tokyo, Japan; Rutgers The State University of New Jersey, UNITED STATES

## Abstract

We propose a heuristic method of using network centralities for constructing small-weight Steiner trees in this paper. The Steiner tree problem in graphs is one of the practical NP-hard combinatorial optimization problems. Given a graph and a set of vertices called terminals in the graph, the objective of the Steiner tree problem in graphs is to find a minimum weight Steiner tree that is a tree containing all the terminals. Conventional construction methods make a Steiner tree based on the shortest paths between terminals. If these shortest paths are overlapped as much as possible, we can obtain a small-weight Steiner tree. Therefore, we proposed to use network centralities to distinguish which edges should be included to make a small-weight Steiner tree. Experimental results revealed that using the vertex or the edge betweenness centralities contributes to making small-weight Steiner trees.

## Introduction

The Steiner tree problem in graphs is one of the practical and important combinatorial optimization problems. For example, it can be used to solve various real-world problems such as the design of communication and power transmission networks [1, 2] and sewer layouts [3]. Although the Steiner tree problem in graphs has many variations [4–6], their purpose is the same: to find the Steiner tree with the minimum weight. Given an undirected weighted graph *G* = (*V*, *E*, *w*) and a set *T* of vertices called terminals in the graph *G*, a Steiner tree is defined as a subtree containing all the terminals, where *V* is a set of vertices, *E* is a set of edges, and *w* is a weight function of edges. The weight of the Steiner tree is the sum of the weights of the edges of the tree. Let *S* be the Steiner tree, where *S* = (*V*_*S*_, *E*_*S*_, *w*), *V*_*S*_ is a subset of *V*, *E*_*S*_ is a subset of *E*, and *V*_*S*_ satisfies *T* ⊆ *V*_*S*_ ⊆ *V*. The objective function *F* of the Steiner tree problem in graphs is defined by

$$\begin{array}{c} F\left(S\right)=\sum_{e\in E_{S}}w\left(e\right). \end{array}$$ (1)

where *w*(*e*) is the weight of the edge *e*.

The Steiner tree problem in graphs has many variations: for example, the terminal Steiner tree problem, the hop-constrained Steiner tree problem, and the prize-collecting Steiner tree problem. The terminal Steiner tree problem [4] is the problem of finding the minimum-weight Steiner tree for which all terminals are leaves. The hop-constrained Steiner tree problem [5] is to find a rooted minimum-weight Steiner tree in that the number of hops from a root to any leaves is smaller than the maximum hops. In the prize-collecting Steiner tree problems [6], not only edges but also vertices have their weight. The objective of the prize-collecting Steiner tree problem is to find a Steiner tree that minimizes the sum of the edge weights included in a tree and the vertex weights that are not included in a tree.

**Fig 1:**
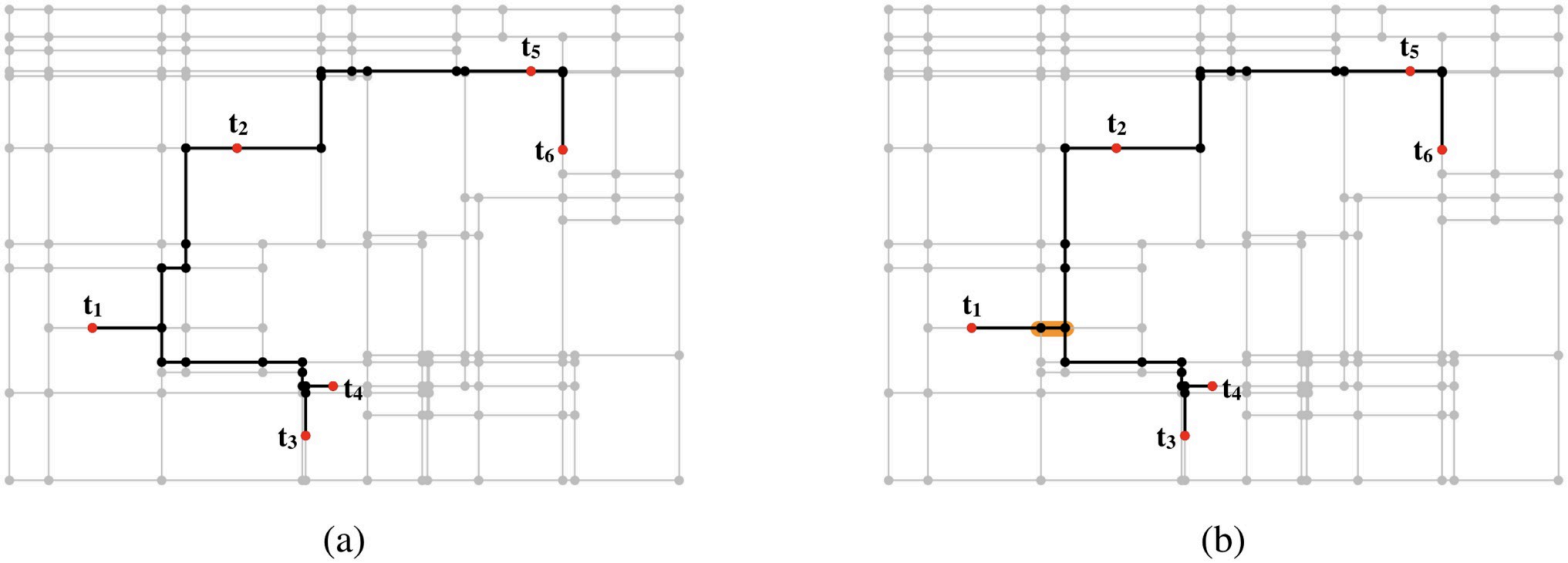
Effects of overlapping the shortest paths. In this example, lin04 in SteinLib [16] was used. Circles indicate vertices and lines indicate edges. Red vertices are terminals, and black vertices and edges are those of the Steiner trees. The weights of each edge correspond to its length. An objective function value of (a) is 1, 267 and that of (b) is 1, 239. In (b), the shortest paths between (*t*_1_, *t*_2_) and (*t*_1_, *t*_4_) share an edge shown in orange. However, in (a), the shortest paths between these pairs of terminals do not overlap. Thus, the objective function value of (b) becomes smaller than that of (a).

Numerous heuristics have been proposed to solve the Steiner tree problem in graphs. To make Steiner trees from an input graph, three heuristics are frequently used [7–11]: the distance network heuristic (DNH) [12], the shortest path heuristic (SPH) [13], and the average distance heuristic (ADH) [14]. These heuristics construct Steiner trees based on the shortest paths [15]. However, these heuristics occasionally produce Steiner trees with large weights because they do not consider overlaps of edges in the shortest paths. The weight of the Steiner tree for a graph becomes small if the shortest paths between terminals in the tree have as many common edges as possible. Fig 1 shows this example. Both Fig 1(a) and 1(b) show Steiner trees of the same inputs. However, the weight of the Steiner tree shown in Fig 1(b) is smaller than that of the Steiner tree in Fig 1(a). The difference in this example comes from an overlap of the shortest paths between (*t*_1_, *t*_2_) and (*t*_1_, *t*_4_). In Fig 1(a), the shortest paths between (*t*_1_, *t*_2_) and (*t*_1_, *t*_4_) do not share the orange-colored edge shown in Fig 1(b), while in Fig 1(b), the shortest paths between (*t*_1_, *t*_2_) and (*t*_1_, *t*_4_) share the orange-colored edge shown. This overlap decreases the weight of the obtained Steiner tree.

To solve this issue, we have already proposed a method that uses the edge betweenness centrality [17]. This method provides information about overlapping edges in the shortest paths between all vertices. Then, a DNH using the edge betweenness centrality successfully decreases the weight of the obtained Steiner trees more than the conventional DNH. However, many other network centralities, defined not only for edges but also for vertices, could be effective for this issue: which network centrality demonstrates the best performance?

From this perspective, in this paper, we aim to clarify which network centrality provides the best performance for solving the Steiner tree problem in graphs by comparing the performance of the three heuristics using five network centralities. In addition, we extend a heuristic to use not only edge network centralities but also vertex network centralities.

## Methods

### Heuristics

In this study, we used the three heuristics to solve the Steiner tree problem in graphs: DNH [12], SPH [13], and ADH [14]. We briefly review these three heuristics in the following.

The DNH [12] uses the shortest paths and a minimum spanning tree to construct a Steiner tree. First, a complete weighted graph comprising all the terminals in *T*, denoted as *H*, is constructed, where the edge weight between terminals *t*_*i*_ and *t*_*j*_ (*t*_*i*_, *t*_*j*_ ∈ *T*, *i* ≠ *j*) is defined by the shortest distance between these terminals in the input graph *G*. Next, a minimum spanning tree of *H*, MST(*H*), is constructed. Then, each edge of MST(*H*) is replaced by the corresponding shortest path in *G*. If multiple shortest paths that have the same weight are obtained, we select one of them randomly. Finally, a Steiner tree is obtained by making a minimum spanning tree of the subgraph induced from the obtained vertex set. This procedure eliminates any loops from the obtained graph. The cost of calculating the DNH depends on the calculation cost of the shortest paths. In addition, the calculation cost of a single source shortest path is $O(|V{|}^{2})$ when using the Dijkstra algorithm [15], where |⋅| indicates the number of elements in a set. When using the Dijkstra algorithm [15], the calculation cost of the DNH becomes $O(|T||V{|}^{2})$, because the DNH repeatedly calculates the single source shortest path |*T*| times.

The SPH [13] uses only the shortest paths to construct a Steiner tree. First, we set *S*_SPH_ = (*V*_SPH_, *E*_SPH_), where *V*_SPH_ = {*r*} and *E*_SPH_ = {∅}; *r* is referred to as a root, which is selected randomly from *T*. Second, including the shortest path between vertices *v* ∈ *V*_SPH_ and *t* ∈ *T*\*V*_SPH_ to *S*_SPH_ (update *V*_SPH_ and *E*_SPH_), is repeated until *T* ⊆ *V*_SPH_. The calculation cost of the SPH is $O(|T||V{|}^{2})$. In addition, Sun *et al.,* proposed the LANCET algorithm [18] to reduce the calculation cost of the SPH. The calculation cost of the SPH can be reduced to O(|T|(|E|+|V|log|V|)) by using the LANCET algorithm.

The ADH [14] also uses the shortest paths to construct a Steiner tree. The ADH starts from a forest comprising |*T*| trees. Each initial tree includes only a single terminal. Then, the ADH repeats the combination of trees until they become a single Steiner tree. In each step, two trees are combined via a vertex *v* that minimizes the following evaluation function *D*(*v*).

$$\begin{array}{c} D\left(v\right)=\underset{1\leq j\leq k}{\text{min}}\sum_{i=0}^{j}\;\frac{d(v,t_{v.i})}{j}, \end{array}$$ (2)

where *k* is the number of trees and is decreased by one when two trees are combined, *t*_*v*.*i*_ is the *i*th nearest tree from *v*, and *d*(*v*, *t*_*v*.*i*_) is the shortest distance between the vertex *v* and the nearest vertex in *t*_*v*.*i*_ from *v*. In the case of calculating Eq (2), *t*_*v*.*i*_ is sorted in ascending order of distance from *v*. Then, the order of *t*_*v*.*i*_ is updated after the two trees are combined. Thus, the ADH needs to calculate the shortest distance between all pairs of vertices in the graph. Therefore, the calculation cost of the ADH is $O(|V{|}^{3})$.

### Network centralities

Network centralities measure the importance of the vertices or edges in a graph or network. In this study, we used five network centralities to solve the Steiner tree problem in graphs: the degree centrality [19], the eigenvector centrality [20], the closeness centrality [21], the vertex betweenness centrality [22], and the edge betweenness centrality [22]. We briefly introduce these five network centralities below.

The degree centrality [19] measures how many vertices are adjacent to a given vertex in the network. The degree centrality of the *i*th vertex *v*_*i*_, *d*_*v*_(*v*_*i*_), is defined

$$\begin{array}{c} d_{v}\left(v_{i}\right)=\sum_{j=1}^{\left|V\right|}A_{ij}\;\left(i=1,\;...,\;|V|\right), \end{array}$$ (3)

where *A*_*ij*_ is the *ij*th element of the adjacency matrix of the network. If an edge between the *i*th and the *j*th vertices exists, *A*_*ij*_ = 1; otherwise, *A*_*ij*_ = 0. The calculation costs of the degree centrality of all vertices are $O(|V{|}^{2})$ when using either the adjacency matrix or the adjacency list.

The eigenvector centrality [20] measures the influence of a vertex in a network. If a vertex *v* connects to vertices with large centralities, the vertex *v* is also assumed to have a large centrality. The eigenvector centrality can be calculated by the power method described as follows:

$$\begin{array}{c} z_{n+1}=Az_{n}\;/\;∥Az_{n}∥\;\left(n=0,\;1,\;2,\;\cdots \right) \end{array}$$ (4)

where ***z***_*n*_ = (*z*_*n*1_, ⋯, *z*_*n*|*V*|_)^⊤^ is the vector whose *i*th element is the centrality of the *i*th vertex, *A* is the adjacency matrix, and ‖*A**z***_*n*_‖ denotes a norm of the vector *A**z***_*n*_. Note that, each element of ***z***_0_ is randomly drawn from a uniform distribution. In the case that *A* is a diagonalizable matrix, ***z***_*n*_ converges to the eigenvector corresponding to the largest eigenvalue of *A*. Thus, for sufficiently large *n*, ***z***_*n*_ is called the eigenvector centrality.

The time complexity of the matrix-vector multiplication is $O(|V{|}^{2})$. However, the number of matrix-vector multiplications required to estimate the eigenvector centrality cannot be known in advance. The number of iterations depends on how accurately the vector is judged to have converged and the value |λ_2_/λ_1_|, where λ_1_ is the largest eigenvalue, and λ_2_ is the second-largest eigenvalue. Thus, it is not easy to write down the time complexity to obtain the eigenvector centrality. It is one of the future issues of our study.

The closeness centrality [21] measures how a vertex is close to all the other vertices in the network. Then, the closeness centrality of the *i*th vertex *v*_*i*_, *c*_*v*_(*v*_*i*_), is defined as follows.

$$\begin{array}{c} c_{v}\left(v_{i}\right)=\frac{1}{\sum_{j=1,j\neq i}^{\left|V\right|}d\left(v_{i},v_{j}\right)}, \end{array}$$ (5)

where *d*(*v*_*i*_, *v*_*j*_) is the shortest distance between the *i*th vertex *v*_*i*_ and the *j*th vertex *v*_*j*_. The calculation cost of the closeness centrality of all vertices is $O(|V{|}^{3})$ because the calculation cost of the single source shortest path is $O(|V{|}^{2})$, which is repeated |*V*| times.

The betweenness centrality [22] detects the central vertices or edges in the network using the shortest path information. If a vertex or an edge frequently appears on the shortest paths of a network, the vertex or the edge has a large betweenness centrality. The betweenness centrality of the *i*th vertex *v*_*i*_, *b*_*v*_(*v*_*i*_), is defined as follows.

$$\begin{array}{c} b_{v}\left(v_{i}\right)=\sum_{s=1,s\neq i}^{\left|V\right|}\sum_{g=1}^{s-1}\frac{P_{v_{i}\left(s,g\right)}}{P_{\left(s,g\right)}}, \end{array}$$ (6)

where *s* and *g* are the starting and goal vertices of the shortest path, *P*_(*s*,*g*)_ is the total number of shortest paths between *s* and *g*, and $P_{v_{i}(s,g)}$ is the total number of shortest paths between *s* and *g* through the *i*th vertex *v*_*i*_.

Similarly, the betweenness centrality of the *i*th edge *e*_*i*_, *b*_*e*_(*e*_*i*_), is defined as follows.

$$\begin{array}{c} b_{e}\left(e_{i}\right)=\sum_{s=1,s\neq i}^{\left|V\right|}\sum_{g=1}^{s-1}\frac{P_{e_{i}\left(s,g\right)}}{P_{\left(s,g\right)}}, \end{array}$$ (7)

where $P_{e_{i}(s,g)}$ is the total number of shortest paths between *s* and *g* through the *i*th edge *e*_*i*_. The calculation cost of both betweenness centralities of all vertices or edges is $O(|V{|}^{3})$ [23]. We can significantly reduce the practical calculation cost of the betweenness centrality by using the hub labeling algorithm [24].

### The proposed method

In order to construct a Steiner tree including edges shared by multiple shortest paths between terminals, we proposed a method for solving the Steiner tree problem in graphs that uses new edge weights based on the weights of input edges and network centralities. Then, the new edge weight function *w*′(*e*_*i*_) of the *i*th edge *e*_*i*_ is defined as follows.

$$\begin{array}{c} w^{\prime }\left(e_{i}\right)=\alpha w\left(e_{i}\right)+\left(1-\alpha \right)\frac{1}{C_{e}\left(e_{i}\right)}, \end{array}$$ (8)

where *w*(*e*_*i*_) is the input edge weight of *e*_*i*_, *C*_*e*_(*e*_*i*_) is one of the network centralities of *e*_*i*_, and *α* is a scaling parameter. A suitable value of *α* strongly depends on the range of the input edge weights. To balance the effect of two terms on the right-hand side of Eq (8), *w*(*e*_*i*_) and 1/*C*_*e*_(*e*_*i*_) were normalized by their maximum values, respectively.

Vertices and edges with large network centralities play important roles in the network. However, the objective of the Steiner tree problem in graphs is to find a Steiner tree with a small weight. To realize the minimization of the total edge weights and the maximization of information of the network centralities in a Steiner tree simultaneously, we use a reciprocal of network centralities. If the network centrality is 0, we set it to unity. This is because it corresponds to the maximum value of the reciprocal of the network centrality.

Many network centralities are defined for the vertices. When we use the network centrality of the vertices, we transform the network centrality of the vertices to that of the edges by

$$\begin{array}{c} C_{e}\left(e_{ij}\right)=\frac{C_{v}\left(v_{i}\right)+C_{v}\left(v_{j}\right)}{2}, \end{array}$$ (9)

where *e*_*ij*_ is the edge between the *i*th vertex *v*_*i*_ and the *j*th vertex *v*_*j*_, and *C*_*v*_(*v*_*i*_) is the network centrality of *v*_*i*_.

We apply the heuristics, the DNH [12], the SPH [13], and the ADH [14], with the network centralities by the following procedure. First, the network centrality of the vertices is converted to that of the edges. Next, *w*′ is calculated using Eq (8). Next, the DNH [12], the SPH [13], or the ADH [14] is then applied to an input graph with *w*′. Then, extra leaves, i.e. non-terminal vertices with a single degree, are removed from the obtained Steiner tree. Finally, *w*′ is replaced with *w* and the weight of the obtained Steiner tree is recalculated. The source code of our proposed method is available on GitHub (https://github.com/misafujita/stp_centrality).

## Results

### Experimental conditions

We evaluated the performance of the heuristics using the network centralities through numerical experiments. We used three heuristics: the DNH, the SPH, and the ADH, and the five network centralities: the degree, the eigenvector, the closeness, the vertex betweenness, and the edge betweenness centralities.

For numerical experiments, we used the benchmark problem sets B, C, D, E, I080, I160, I320, and I640 in Steinlib [16]. Table 1 shows the number of instances, the number of vertices (|*V*|), the number of undirected edges (|*E*|), the number of terminals (|*T*|), and the densities of edges against all combinations of vertices (2|*E*|/{|*V*|(|*V*| − 1)}) for these benchmark problem sets. The densities of the benchmark problem sets B, C, D, and E are low and the edge weights are assigned according to uniformly distributed random numbers between 1 and 10. On the other hand, the range of the density of the edges of the benchmark problem sets I080, I160, I320, and I640 is wide and the edge weights are assigned using random numbers according to a normal distribution. The average of the normal distribution of the edge weights between non-terminals is 100, between the terminal and non-terminal is 200, and between the terminals is 300.

**Table 1:** Statistical features of the benchmark problem sets [16].

Name	# of instances	|*V*|	|*E*|	|*T*|	$\frac{2|E|}{\left|V|(|V|-1\right)}$
B	18	50–100	63–200	9–50	0.025–0.082
C	20	500	625–12,500	5–250	0.005–0.100
D	20	1,000	1,250–25,000	5–500	0.003–0.050
E	20	2,500	3,125–62,500	5–1,250	0.001–0.020
I080	100	80	120–3,160	6–20	0.038–1.000
I160	100	160	240–12,720	7–40	0.019–1.000
I320	100	320	480–51,040	8–80	0.009–1.000
I640	100	640	960–204,480	9–160	0.005–1.000

If the scaling parameter *α* of Eq (8) is set to zero, *w*′(*e*_*i*_) = 1/*C*_*e*_(*e*_*i*_). If *α* is set to unity, *w*′(*e*_*i*_) = *w*(*e*_*i*_). Thus, *w*′(*e*_*i*_) differs from the conventional method (i.e. without using network centralities) in case 0 ≤ *α* < 1. Then, the value of *α* was selected from {0.0, 0.1, ⋯, 0.9} for each instance based on the results of pre-experiments. The details of pre-experiments are shown in the S1 File.

The performances of the conventional and the proposed methods were compared based on the average gaps and average CPU time. The gap is defined as (*F*(*S*) − *F*(S*))/*F*(S*) × 100 [%], where *F*(*S*) is the objective function value of the obtained Steiner tree *S*, and *F*(S*) is the objective function value of the optimum Steiner tree S*. Each method was implemented in C language, using the GCC compiler (ver. 4.2.1). All numerical experiments were conducted on an iMacPro (2017) with a 3.2GHz Intel Xeon W processor and 64GB of 2, 666MHz DDR4 random access memory. We constructed 50 Steiner trees for each instance and averaged the gaps and CPU time.

### Gaps and calculation time

**Fig 2:**
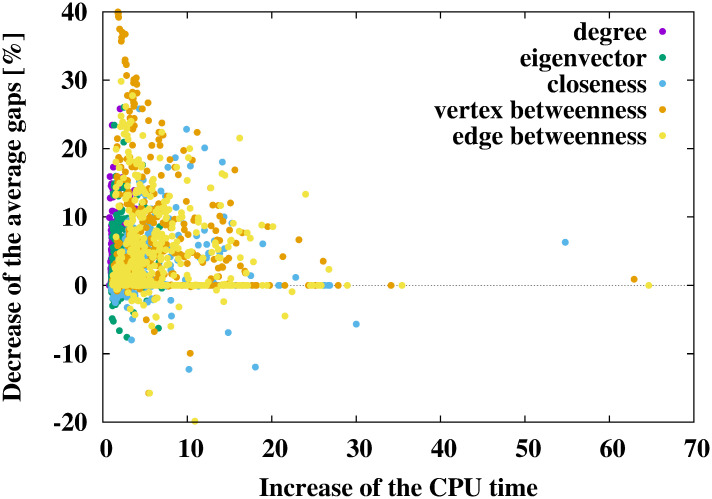
Relationship between the increase in the CPU time (average CPU time of the proposed method divided by that of the conventional method) and the decrease in the average gaps (average gap of the conventional method minus that of the proposed method) when using the DNH.

**Fig 3:**
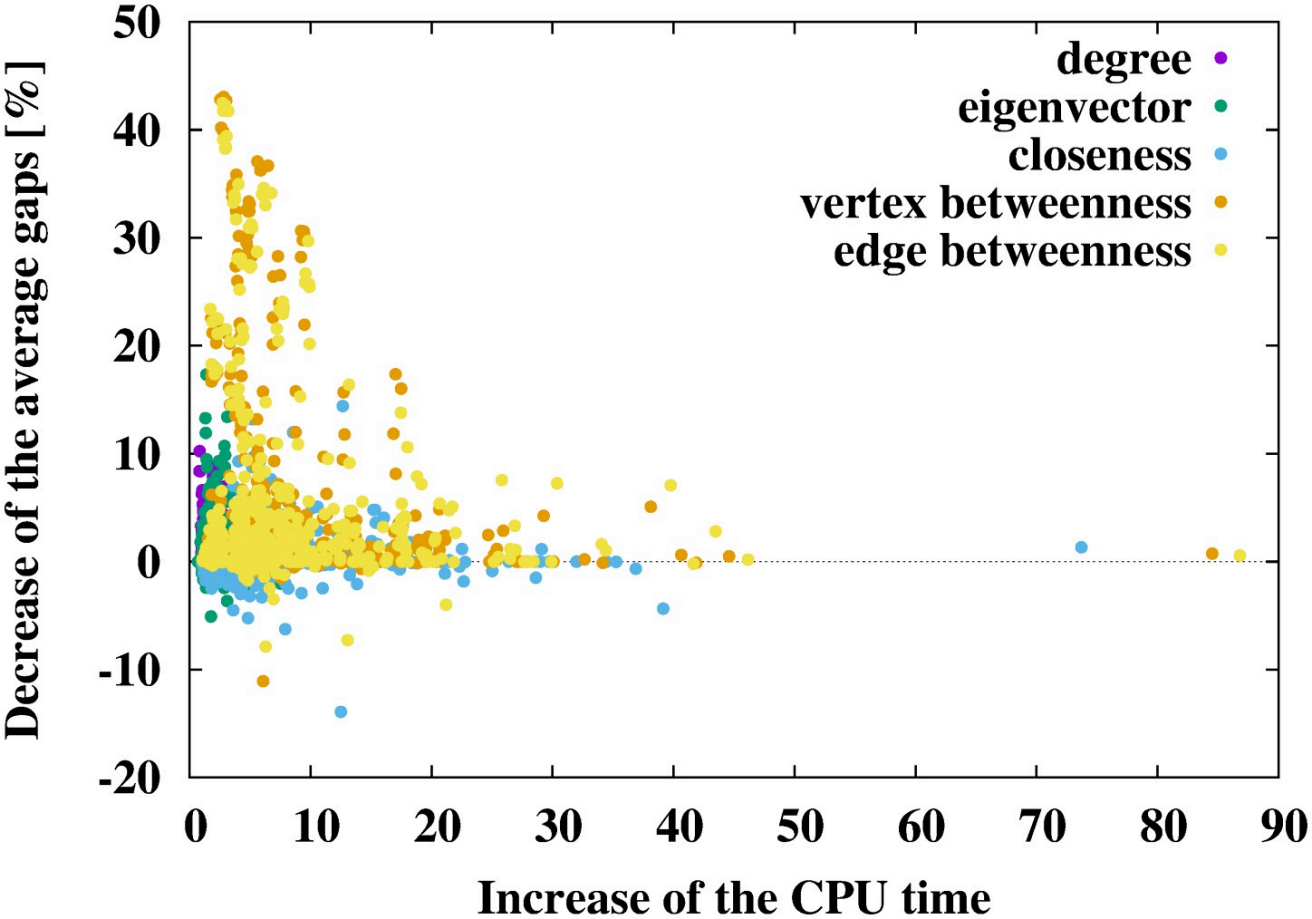
Relationship between the increase in the CPU time (average CPU time of the proposed method divided by that of the conventional method) and the decrease in the average gaps (average gap of the conventional method minus that of the proposed method) when using the SPH.

**Fig 4:**
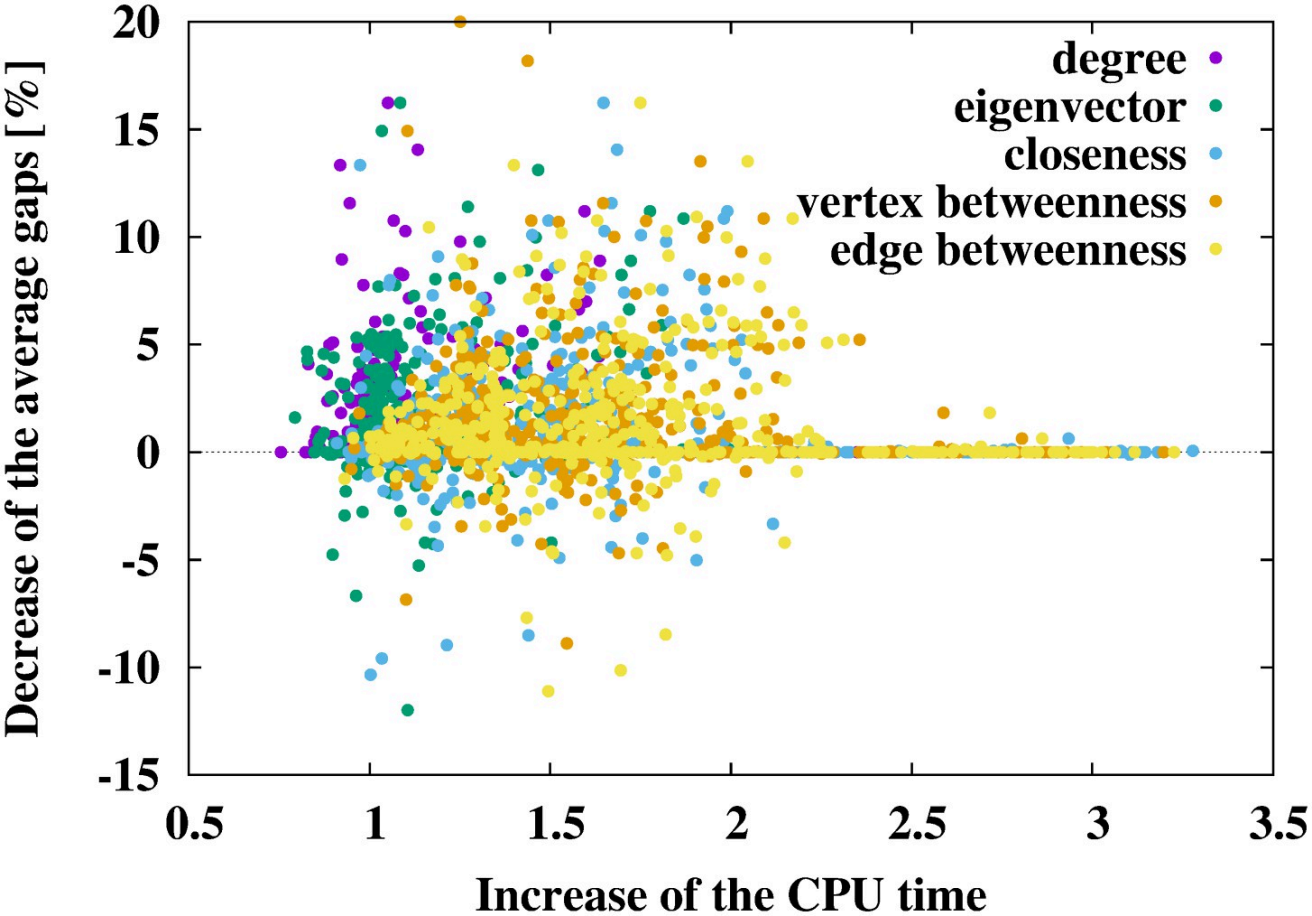
Relationship between the increase in the CPU time (average CPU time of the proposed method divided by that of the conventional method) and the decrease in the average gaps (average gap of the conventional method minus that of the proposed method) when using the ADH.

The results of the numerical experiments are shown in Tables 2–7 and Figs 2–4. In Tables 2, 4 and 6, the cases with the smallest gaps are shown in bold. The results of numerical experiments for each benchmark instance are shown in the S1 File.

**Table 2:** Average gaps [%] of using the DNH.

	without network centrality	with network centrality				
		degree	eigenvector	closeness	betweenness	
					vertex	edge
B	2.73	2.18	2.42	2.37	1.74	**1.39**
C	5.91	5.38	5.84	4.99	4.75	**4.03**
D	5.76	5.72	5.86	7.14	**4.31**	4.38
E	8.93	8.08	8.99	8.60	7.24	**6.72**
I080	17.90	15.45	15.02	15.64	**12.60**	13.52
I160	22.14	19.96	20.01	20.13	**14.60**	16.48
I320	24.06	22.05	22.40	22.47	**16.58**	18.91
I640	26.35	24.37	24.47	25.12	**18.61**	20.38
Average	14.22	12.90	13.13	13.31	**10.05**	10.73

**Table 3:** Average CPU time [s] of using the DNH.

	without network centrality	with network centrality				
		degree	eigenvector	closeness	betweenness	
					vertex	edge
B	0.00	0.00	0.00	0.00	0.00	0.00
C	0.16	0.16	0.18	0.30	0.34	0.36
D	1.11	1.11	1.21	1.74	1.96	2.00
E	16.85	16.86	16.85	20.64	23.49	25.20
I080	0.00	0.00	0.00	0.01	0.01	0.01
I160	0.01	0.01	0.01	0.03	0.03	0.03
I320	0.03	0.04	0.04	0.19	0.19	0.20
I640	0.24	0.24	0.28	1.57	1.49	1.54
Average	2.30	2.30	2.32	3.06	3.44	3.67

**Table 4:** Average gaps [%] using the SPH.

	without network centrality	with network centrality				
		degree	eigenvector	closeness	betweenness	
					vertex	edge
B	1.81	1.46	1.47	1.77	1.09	**0.78**
C	3.37	3.28	3.26	3.07	**2.17**	2.47
D	3.85	3.84	3.69	4.51	2.89	**2.39**
E	5.30	5.05	5.24	5.41	4.06	**3.47**
I080	14.68	13.40	12.88	13.63	**9.78**	10.12
I160	17.73	16.93	16.49	17.04	**11.10**	11.25
I320	19.37	18.72	18.37	18.80	**11.99**	12.28
I640	20.59	19.85	19.84	20.57	**12.66**	12.93
Average	10.84	10.32	10.15	10.60	6.97	**6.96**

**Table 5:** Average CPU time [s] of using the SPH.

	without network centrality	with network centrality				
		degree	eigenvector	closeness	betweenness	
					vertex	edge
B	0.00	0.00	0.00	0.00	0.00	0.00
C	0.11	0.11	0.13	0.26	0.29	0.31
D	0.78	0.78	0.86	1.38	1.54	1.62
E	12.21	12.21	12.45	16.58	18.73	19.50
I080	0.00	0.00	0.00	0.00	0.00	0.01
I160	0.00	0.01	0.01	0.03	0.03	0.03
I320	0.03	0.03	0.03	0.18	0.18	0.19
I640	0.17	0.17	0.21	1.51	1.44	1.47
Average	1.66	1.66	1.71	2.49	2.78	2.89

**Table 6:** Average gaps [%] of using the ADH.

	without network centrality	with network centrality				
		degree	eigenvector	closeness	betweenness	
					vertex	edge
B	1.50	1.31	0.83	1.25	0.70	**0.61**
C	3.90	3.40	3.76	3.60	**2.70**	4.48
D	3.99	3.37	3.27	3.54	**2.72**	3.24
E	5.29	3.99	4.43	4.71	**3.15**	3.73
I080	3.26	1.76	1.50	1.65	1.44	**1.25**
I160	3.31	1.86	2.18	2.06	1.88	**1.79**
I320	3.05	2.30	1.93	2.14	**1.84**	1.91
I640	3.91	2.81	2.73	2.79	**2.48**	2.54
Average	3.53	2.60	2.58	2.72	**2.11**	2.44

**Table 7:** Average CPU time [s] of using the ADH.

	without network centrality	with network centrality				
		degree	eigenvector	closeness	betweenness	
					vertex	edge
B	0.01	0.01	0.01	0.01	0.01	0.01
C	1.28	1.26	1.29	1.40	1.50	1.54
D	10.38	10.29	10.72	10.73	11.44	11.92
E	189.50	188.66	186.46	187.41	197.97	203.73
I080	0.00	0.01	0.01	0.01	0.01	0.01
I160	0.02	0.03	0.03	0.05	0.05	0.05
I320	0.20	0.20	0.21	0.36	0.36	0.36
I640	1.68	1.67	1.72	3.05	3.01	3.08
Average	25.38	25.27	25.06	25.38	26.79	27.59

Tables 2 and 3, and Fig 2 show the results of using the DNH [12]. As shown in Table 2, the DNH with network centralities obtained Steiner trees with a smaller gap than those obtained by the DNH without network centralities. In particular, the DNH with the vertex betweenness centrality exhibited the best performance, reducing the gaps by approximately 14.22 − 10.05 = 4.17%. As shown in Table 3, the DNH with the degree centrality did not increase the CPU time. However, using the eigenvector, the closeness, the vertex betweenness, and the edge betweenness centralities increased the CPU time by at most 3.67/2.30 ≃ 1.60 times compared to the conventional DNH. This is because the calculation time of network centralities is added to that of the DNH in our method. Thus, it is important to find a balance between the increase in the calculation time and the decrease in the average gap. The relationship between the increase in the calculation time and the decrease of the average gap is shown in Fig 2. In Fig 2, the horizontal axis expresses the increase in the calculation time compared to the conventional DNH and the vertical axis expresses the decrease of the average gaps compared to the conventional DNH. Different colors express methods using different network centralities. Each point corresponds to each instance. Consequently, 478 (instances) ×5 (network centralities) = 2, 390 points are plotted in Fig 2. As shown in Fig 2, if network centralities are used, the average gaps decrease for many instances. The increase of the calculation time of the proposed method was less than ten times the CPU time of the conventional method in many cases. In particular, if vertex betweenness centrality is used, the average gaps decreased by approximately 40% with the twice CPU time of the conventional method for some instances. These results indicate that using network centralities, especially vertex betweenness centrality, led to good performance when using the DNH.

Tables 4 and 5, and Fig 3 show the results of using the SPH [13]. As shown in Table 4, similar to the results of the DNH, the SPH with network centralities obtained Steiner trees with a smaller gap than the SPH without network centralities. In particular, the SPH with the edge betweenness centrality shows the best performance and improves the average gaps by approximately 10.84 − 6.96 = 3.88%. From this result, the SPH with network centralities obtains Steiner trees with smaller gaps than those obtained by the DNH. The average gap of the SPH with the edge betweenness centrality (6.96%) was smaller than that of the DNH with the vertex betweenness centrality (10.05% in Table 2). From Table 5, the SPH with the degree centrality shows the same calculation time compared to the SPH without degree centrality. By contrast, using the eigenvector, the closeness, the vertex betweenness, and the edge betweenness centralities increased the CPU time by at most 2.89/1.66 ≃ 1.74 times that of the conventional SPH. The computational cost of the SPH was the same as that of the DNH. Thus, the trends of their calculation time were the same. As seen in Fig 3, using network centralities decreased the average gaps for many instances within ten times the conventional method’s CPU time. These results indicate that using network centralities, especially edge betweenness centrality, led to good performance when using the SPH. In addition, the combination of network centralities and the SPH exhibited better performance than the combination of network centralities and the DNH.

Tables 6 and 7, and Fig 4 show the results of using the ADH [14]. As shown in Table 6, the ADH without any network centralities obtained smaller-gap Steiner trees (3.53%) than the DNH with the vertex betweenness centrality (10.05% from Table 2) and the SPH with the edge betweenness centrality (6.96% from Table 4). It should be noted that the calculation cost of the ADH was larger than that of the DNH and the SPH. The calculation cost of the ADH is $O(|V{|}^{3})$, and that of the DNH and the SPH is $O(|T||V{|}^{2})$. The ADH with network centralities obtained Steiner trees with a smaller gap than those without any network centralities. In particular, the ADH with the vertex betweenness centrality showed the best performance, improving the gaps by approximately 3.53 − 2.11 = 1.42%. As shown in Table 7, the CPU time of the ADH with the degree and eigenvector centralities was shorter than that without network centrality. One of the reasons why the CPU time of the ADH with some network centralities was relatively short is that the order of the calculation costs of these network centralities was smaller than or equal to the calculation cost of the ADH. As shown in Fig 4, using network centralities decreased the average gaps for many instances within twice the conventional method’s CPU time.

The results of the numerical experiments indicate the following consequences:

1. Using the network centrality can reduce the gaps of the obtained Steiner trees with major heuristics: the DNH, the SPH, and the ADH.
2. Using the vertex or the edge betweenness centralities shows the best performance among the degree, the eigenvector, the closeness, the vertex or the edge betweenness centralities.
3. The increase of the average CPU time of heuristics with network centralities from the one without any network centralities is approximately ten times for the DNH or the SPH and approximately twice for the ADH.

### Parameter tuning

The proposed method has a scaling parameter *α* which must be set to an appropriate value. However, this procedure requires a long calculation time. Thus, to tune the value of *α* more easily, we investigated the relationship between *α* and the improvement in the gaps for the benchmark problem sets B, C, D, E, I080, I160, I320, and I640. Parameters were tuned per instance, and we averaged their average gaps.

**Fig 5:**
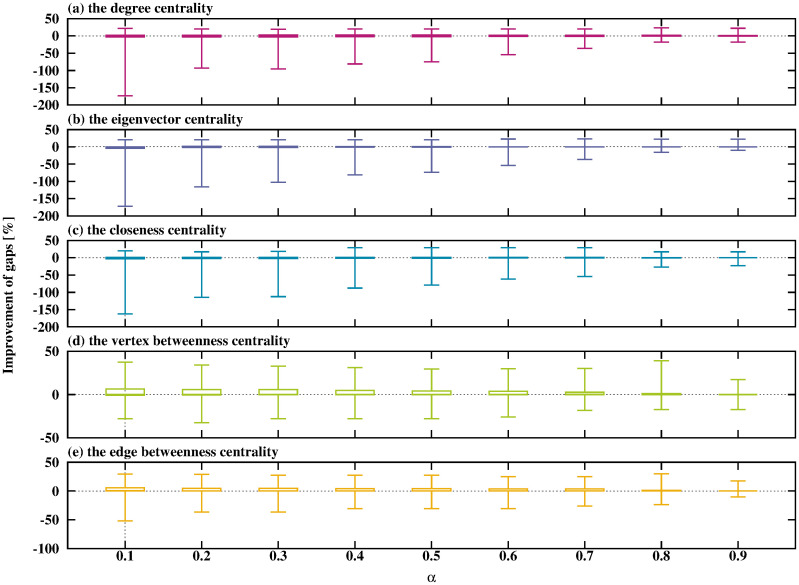
Relationship between *α* and the improvement in gaps when using the DNH.

**Fig 6:**
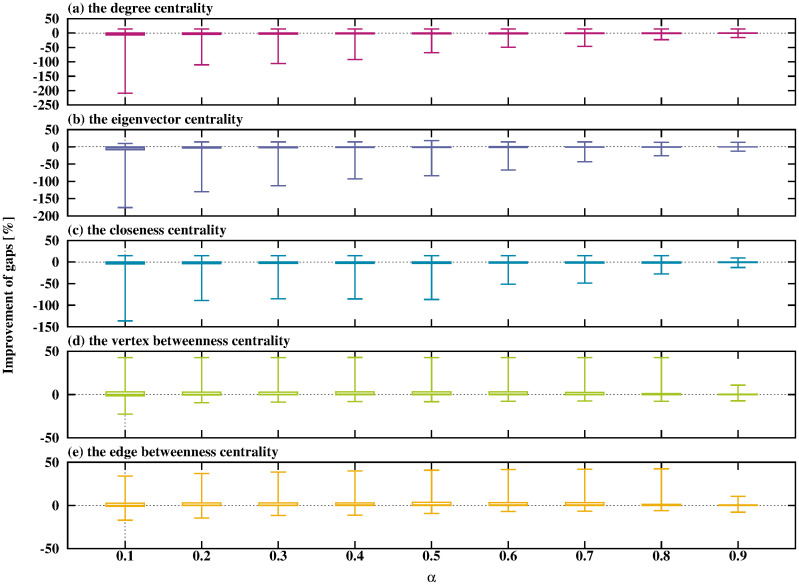
Relationship between *α* and the improvement in gaps when using the SPH.

**Fig 7:**
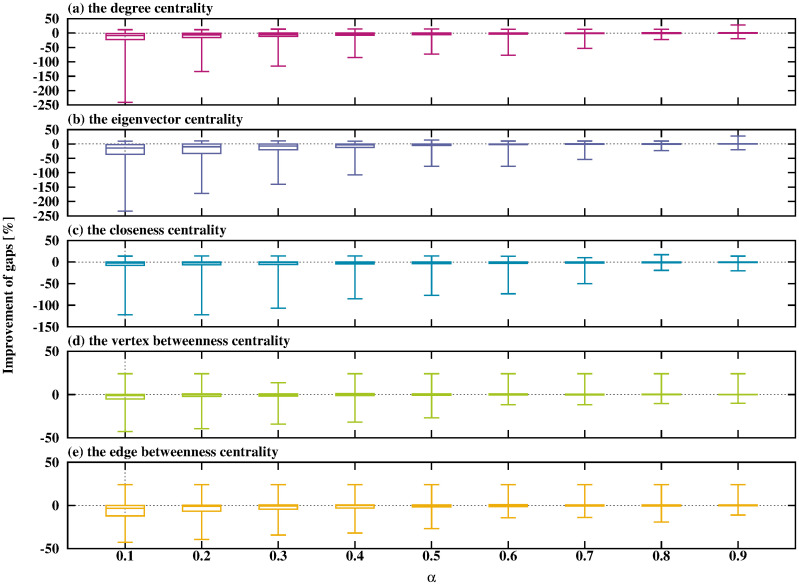
Relationship between *α* and the improvement in gaps when using the ADH.

Figs 5–7 show the relationship between *α* and the average decrease in the gaps. Positive values indicate that the proposed method shows better performance than the conventional method. Different colors indicate the use of different network centralities.

From Fig 5, the maximum value of the average decrease in the gaps by the DNH is in the positive region and the minimum value of them is in the negative region, regardless of the types of network centralities and the values of *α*. This means that using network centralities assists in constructing a small-weight Steiner tree for some instances. In the case of using the vertex or the edge betweenness centralities, the upper quartiles are on the positive region. The DNH performs the best performance when using the vertex betweenness centrality with small *α*.

From Fig 6, the SPH and the DNH show similar trends for the type of network centrality and the value of *α*. The SPH shows the best performance when using the edge betweenness centrality with small *α*.

As shown in Fig 7, large *α* reduces the negative region of the average decrease by using any type of network centralities. The ADH shows the best performance when using the vertex betweenness centrality with large *α*.

## Conclusions

Selecting a heuristic is one of the most important steps to solve NP-hard combinatorial optimization problems such as the Steiner tree problem in graphs. Many heuristics to solve the Steiner tree problem in graphs use the shortest path between vertices and have shown good performance. However, if multiple shortest paths exist between vertices, using shortest paths sometimes results in a Steiner tree with a large weight. In this case, if the selected shortest paths share common edges as much as possible, the weight of the Steiner tree or the sum of the weights of the edges included in the Steiner tree becomes small. From this perspective, using an edge betweenness centrality can distinguish which shortest paths contribute to making a Steiner tree with minimal weight. This method successfully reduces the weight of the obtained Steiner tree when compared to the method that does not use the edge betweenness centrality. However, the following points were unclear: (1) Does the use of network centralities lead to good performance with any type of heuristic? (2) What is the most efficient network centrality to obtain a Steiner tree with a small weight? (3) To what extent does the calculation time increase when using the network centralities?

To answer these points, we first modified a heuristic using edge network centralities to a version using vertex network centralities; this is because many network centralities are defined for vertices. Based on this approach, we conducted some numerical experiments. We solved 478 benchmark instances in SteinLib using 15 combinations of heuristics and network centralities, which included three heuristics: the DNH, the SPH, and the ADH, and five network centralities: the degree, the eigenvector, the closeness, the vertex betweenness, and the edge betweenness centralities. We evaluated the average gap from the optimum solutions and the calculation time of each method.

The results of numerical experiments revealed the following:

1. Using network centralities successfully reduces the weight of the obtained Steiner trees for any major heuristics.
2. Using the vertex or the edge betweenness centralities shows the best performance among major network centralities.
3. Using network centralities can reduce the gap from the optimum solution by approximately 15% within ten times the calculation time in the cases of using the DNH or the SPH and within twice the calculation time in the case of using the ADH for many instances.

These results indicate that network centrality, especially the vertex or the edge betweenness centralities can be a good indicator for constructing Steiner trees with small weights.

The practical calculation cost of the vertex or the edge betweenness centralities can be reduced by applying the hub labeling algorithm [24]. Thus, using the hub labeling algorithm to obtain the vertex or the edge betweenness centralities is one of the future issues of our proposed method.

The original method of the DNH and the SPH can guarantee their theoretical approximation ratios because these methods use the original edge weight. However, our proposed method uses a new edge weight that merges the original edge weight and its network centrality. Thus, one of the important future issues is to guarantee the theoretical approximation ratio of our proposed method.

## Supporting information

S1 File Supporting data. (PDF)
