# Supplementary material for: A heuristic method for solving the Steiner tree problem in graphs using network centralities
Source: PLoS One. 2024 Jun 6;19(6):e0303764. doi: 10.1371/journal.pone.0303764 (PMC11156324; doi:10.1371/journal.pone.0303764)
Supplement: S1 File — (PDF) [file pone.0303764.s001.pdf]

Supplementary Information: A heuristic method for solving the Steiner tree problem in graphs using network centralities [1]

Misa Fujita[a,b,*], Yutaka Shimada[c], Takayuki Kimura[d], Tohru Ikeguchi[b,e]

[a] Department of Electrical and Electronic Engineering, School of Engineering, Chukyo University, Nagoya-shi, Aichi, Japan

[b] Department of Management Science, Graduate School of Engineering, Tokyo University of Science, Katsushika-ku, Tokyo, Japan

[c] Graduate School of Science and Engineering, Saitama University, Sakura-ku, Saitama, Japan

[d] Department of Electrical, Electronics and Communication Engineering, Faculty of Fundamental Engineering, Nippon Institute of Technology, Miyashiro-cho, Minami-saitama-gun, Saitama, Japan

[e] Department of Information and Computer Technology, Faculty of Engineering, Tokyo University of Science, Katsushika-ku, Tokyo, Japan

[*] Corresponding author

E-mail: m-fujita@sist.chukyo-u.ac.jp (MF)

Contents

S1 How to set the value of parameter α **2**

S2 Gaps and calculation time for each instance **5**

[1] All relevant data are available on GitHub (`https://github.com/misafujita/stp_centrality`).

S1. How to set the value of parameter α

As the most essential point of our proposed method, the newly defined edge weight w' is used instead of the original edge weight w. Here, w' is determined from two pieces of information: w and the network centrality (see Eq. (8) in the main text). The balance between such information is regulated by the parameter α. If $\alpha = 0.0$, w' equals the reciprocal of the network centrality. However, if $\alpha = 1.0$, w' equals w. Thus, we should set $0.0 \leq \alpha < 1.0$ to distinguish between the result of the conventional method and that of our proposed approach. We can obtain Steiner trees with a small weight when α is set to a suitable value.

An example of the Steiner trees obtained with a different α is shown in Fig. S1. In Fig. S1, the weights of each edge correspond to its length. In Fig. S1, the Steiner tree in (b) is the Steiner tree with the smallest weight. The weight of the Steiner tree in (a) is large because the original edge weight w is ignored when $\alpha = 0.0$. However, the weight of the Steiner tree in (c) is also large because the network centrality is ignored when $\alpha = 1.0$. Therefore, the value of α strongly affects the weights of the Steiner trees obtained. However, we do not know the best balance of both terms, namely, the best value of α. To select the best value of α, we changed α from 0.0 to 0.9 per 0.1. We select the value of α that can obtain the Steiner tree with the smallest weight for each instance.

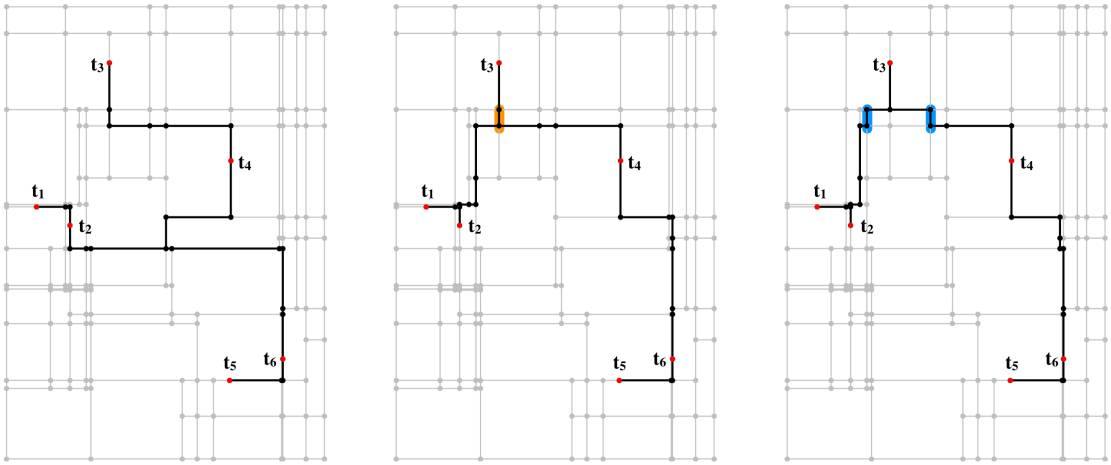

(a) $\alpha = 0.0$ (Gap = 17.92%) (b) $\alpha = 0.5$ (Gap = 0.00%) (c) $\alpha = 1.0$ (Gap = 2.26%)

Figure S1. Steiner trees obtained by the DNH using the edge betweenness centrality with different α's. The instance used in this figure is lin04 in SteinLib [1]. Here, α controls the balance of information between the input edge weight $w(e_i)$ and the edge betweenness centrality $1/C_e(e_i)$. In the case that $\alpha = 0.0$, the new edge weight w' equals the reciprocal of the network centrality. In the case that $\alpha = 1.0$, $w'(e_i)$ equals the input edge weight (the same as the conventional method). The weight of the Steiner tree in (b) is the smallest among them. The weight of the Steiner tree in (a) is the largest because the input edge weight is ignored. The weight of the Steiner tree in (c) is large because the blue edges do not overlap (these paths are overlapped in (b), shown as orange edges).

The relationships between α and an improvement in the gaps for the benchmark problem set I080 (consisting of 100 instances) are shown in Figs S2, S3, and S4. Figure S2 shows the relationship between α and an improvement ratio [%] of the gaps obtained using the DNH [2]. The horizontal axis is the instance index,

the vertical axis is the α, and colors indicate an improvement in the gaps from the conventional method (i.e. without the use of network centralities). We plot the results for $0.1 \leq \alpha \leq 0.9$.

From Fig. S2, the use of network centralities generally improves the gaps because warm-colored areas (the improvement ratios in the gaps are positive) are wider than the cool-colored areas (the improvement ratios in the gaps are negative). For the $11 \sim 15, 36 \sim 40, 61 \sim 65$, and $86 \sim 90$th instances, the improvement ratios in the gaps are zero (white colored). This means that the use of network centralities does not affect these instances. These instances are complete graphs. In complete graphs, all vertices or edges have similar network centralities. Thus, applying network centralities has no effect.

Figure S3 shows the relationship between α and improvement ratios [%] in the gaps obtained using the SPH [3]. From Fig. S3, the use of network centralities generally helps improve the gaps because warm-colored areas are wider than cool-colored areas.

Figure S4 shows the relationship between α and improvement ratios [%] in the gaps obtained when using the ADH [4]. From Fig. S4, the range of improvement ratios in the gaps is smaller than that of the DNH and SPH because the conventional ADH obtains smaller gap solutions than one of them. Because warm-colored areas are wider than cool-colored areas, the use of network centralities improves the gaps. For the complete graph (the $11 \sim 15, 36 \sim 40, 61 \sim 65$, and $86 \sim 90$th) instances, the improvement in the gaps of a small α is negative except for the use of the closeness centrality. These results indicate that we should use a conventional heuristic method for complete graph instances.

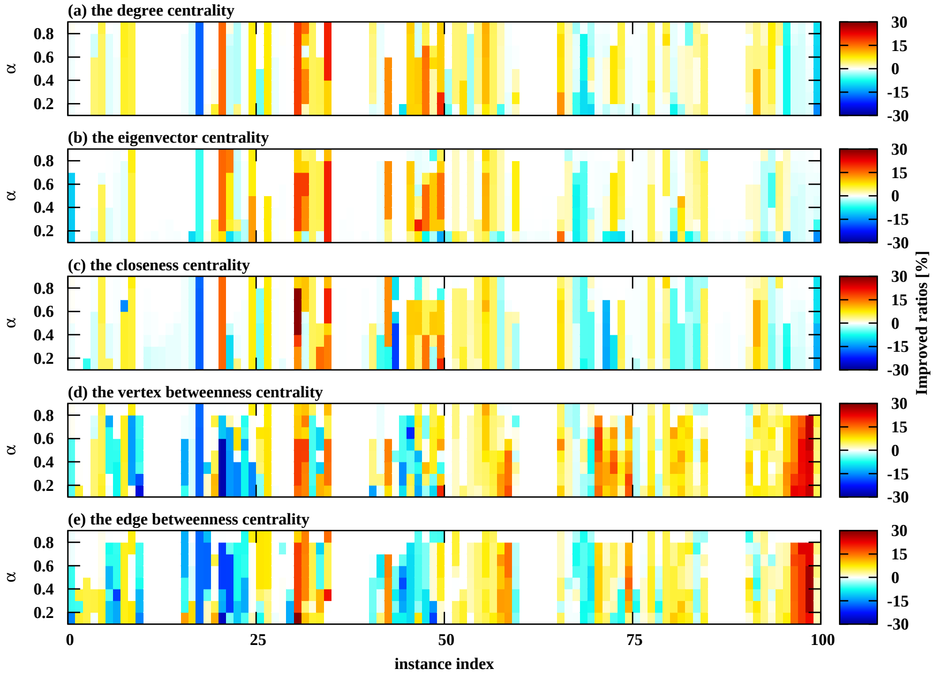

Figure S2. The relationships between α and the improved ratios obtained when using the DNH. The results are shown for all instances in I080.

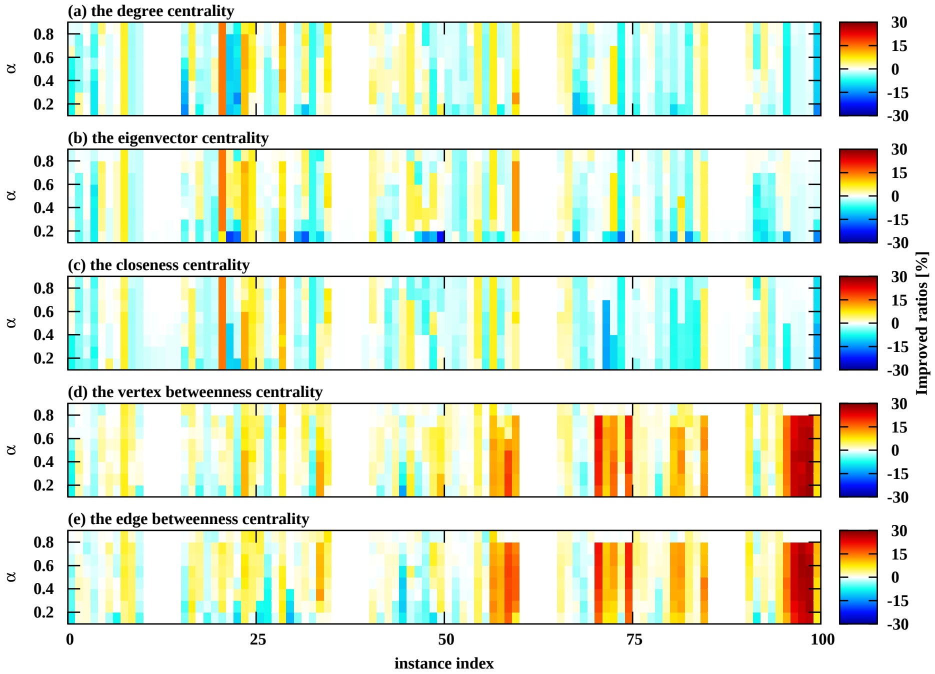

Figure S3. The relationships between α and improved ratios obtained by using the SPH. The results are shown for all instances in I080.

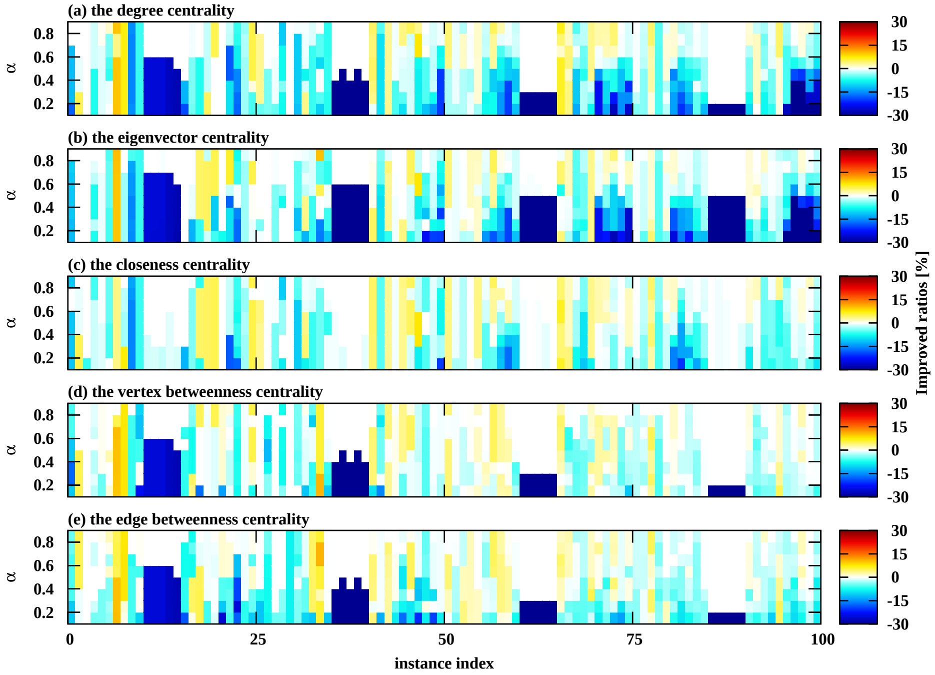

Figure S4. The relationships between α and improved ratios obtained using the ADH. The results are shown for all instances in I080.

S2. Gaps and calculation time for each instance

We use eight benchmark problem sets, B, C, D, E, I080, I160 I320, and I640 in Steinlib [1] to evaluate the performance of the proposed method, namely the heuristic methods using the network centralities. Each benchmark problem set consists of many instances (please see Table 1 in the main text). We showed the results of the average gaps and average time per benchmark problem set in the main text. Thus, in this supplementary information, we showed the best α, average gaps, and average time for each instance.

Tables S1–S24 show the results of the numerical experiments for each instance. In Tables S1–S24, "α" indicates the best value of α that minimizes the average gaps, "gap" is the average gap, and "time" is the average CPU time. If the average gap is minimized for various values of α, we select their minimum values. If the gap of the proposed method is smaller than the original method, the gap is shown in bold type. In addition, texts with a gray background indicate that these are complete graph instances.

References

[1] T. Koch, A. Martin, S. Voß, SteinLib: An updated library on Steiner tree problems in graphs, in: X. Z. Cheng, D.-Z. Du (Eds.), Steiner Trees in Industry, Springer US, Boston, MA, 2001, pp. 285–325. doi:10.1007/978-1-4613-0255-1_9.

[2] L. Kou, G. Markowsky, L. Berman, A fast algorithm for Steiner trees 15 (1981) 141–145. doi:10.1007/BF00288961.

[3] H. Takahashi, A. Matsuyama, An approximate solution for the Steiner problem in graphs, Mathematica Japonica 24 (6) (1980) 573–577.

[4] V. J. Rayward-Smith, The computation of nearly minimal Steiner trees in graphs, International Journal of Mathematical Education in Science and Technology 14 (1) (1983) 15–23. doi:10.1080/0020739830140103.

Table S1. Results of the benchmark problem set B by using the DNH.

	without		with centrality															
	centrality			degree			eigenvector			closeness			vertex betweenness			edge betweenness	
name	gap	time	α	gap	time	α	gap	time	α	gap	time	α	gap	time	α	gap	time
b01	0.00	0.00	0.50	0.00	0.00	0.30	0.00	0.00	0.40	0.00	0.00	0.10	0.00	0.00	0.00	0.00	0.00
b02	7.42	0.00	0.90	**6.02**	0.00	0.70	**6.02**	0.00	0.10	**7.23**	0.00	0.00	**3.61**	0.00	0.10	**0.00**	0.00
b03	0.22	0.00	0.80	**0.00**	0.00	0.10	**0.00**	0.00	0.70	**0.00**	0.00	0.30	**0.00**	0.00	0.10	**0.00**	0.00
b04	0.00	0.00	0.80	8.47	0.00	0.90	8.47	0.00	0.90	5.08	0.00	0.30	5.08	0.00	0.10	0.00	0.00
b05	0.00	0.00	0.90	0.00	0.00	0.80	0.00	0.00	0.80	0.00	0.00	0.40	0.00	0.00	0.10	0.00	0.00
b06	1.64	0.00	0.80	1.64	0.00	0.70	1.64	0.00	0.60	4.10	0.00	0.30	4.10	0.00	0.30	4.10	0.00
b07	0.00	0.00	0.80	0.00	0.00	0.40	0.00	0.00	0.40	0.00	0.00	0.10	0.00	0.00	0.10	0.00	0.00
b08	0.00	0.00	0.60	0.00	0.00	0.10	0.00	0.00	0.60	0.00	0.00	0.10	0.00	0.00	0.10	0.00	0.00
b09	0.45	0.00	0.70	0.45	0.00	0.40	0.91	0.00	0.60	0.45	0.00	0.50	0.45	0.00	0.10	**0.00**	0.00
b10	7.49	0.00	0.40	**5.81**	0.00	0.40	**5.81**	0.00	0.70	**5.81**	0.00	0.10	**1.16**	0.00	0.00	**4.65**	0.00
b11	4.55	0.00	0.60	**2.27**	0.00	0.70	4.55	0.00	0.50	4.55	0.00	0.20	**2.27**	0.00	0.00	**0.00**	0.00
b12	0.00	0.00	0.80	0.00	0.00	0.80	0.00	0.00	0.90	0.00	0.00	0.60	0.00	0.00	0.10	0.00	0.00
b13	6.06	0.00	0.00	**2.42**	0.00	0.90	6.06	0.00	0.00	**3.64**	0.00	0.00	**3.64**	0.00	0.20	6.06	0.00
b14	0.85	0.00	0.80	0.85	0.00	0.40	0.85	0.00	0.60	0.85	0.00	0.50	0.85	0.00	0.50	0.85	0.00
b15	0.00	0.00	0.90	0.00	0.00	0.50	0.00	0.00	0.70	0.00	0.00	0.70	0.00	0.00	0.80	0.00	0.00
b16	7.09	0.00	0.50	**6.30**	0.00	0.70	**5.51**	0.00	0.50	**6.30**	0.00	0.40	**5.51**	0.00	0.10	**5.51**	0.00
b17	1.53	0.00	0.70	2.29	0.00	0.80	2.29	0.00	0.80	2.29	0.00	0.10	2.29	0.00	0.10	1.53	0.00
b18	1.38	0.00	0.80	2.75	0.00	0.30	1.38	0.00	0.90	2.29	0.00	0.40	2.29	0.00	0.10	2.29	0.01
average	2.15	0.00	-	2.18	0.00	-	2.42	0.00	-	2.37	0.00	-	**1.74**	0.00	-	**1.39**	0.00
				(0.03)			(0.27)			(0.22)			(-0.41)			(-0.76)	

Table S2. Results of the benchmark problem set C by using the DNH.

	without		with centrality															
	centrality			degree			eigenvector			closeness			vertex betweenness			edge betweenness	
name	gap	time	α	gap	time	α	gap	time	α	gap	time	α	gap	time	α	gap	time
c01	3.53	0.00	0.20	**2.35**	0.00	0.10	3.53	0.01	0.50	**2.35**	0.04	0.10	**0.00**	0.04	0.10	**1.18**	0.04
c02	0.00	0.01	0.90	0.00	0.01	0.10	0.00	0.01	0.70	0.00	0.04	0.10	0.00	0.05	0.10	0.00	0.05
c03	1.19	0.03	0.90	**0.93**	0.03	0.90	1.99	0.04	0.90	1.72	0.07	0.70	1.99	0.07	0.50	1.99	0.08
c04	2.69	0.05	0.80	**2.59**	0.05	0.30	**2.41**	0.06	0.80	**2.59**	0.09	0.30	**1.76**	0.09	0.70	**1.58**	0.10
c05	0.42	0.11	0.90	0.44	0.11	0.10	0.44	0.11	0.90	0.44	0.14	0.60	0.44	0.15	0.10	0.44	0.15
c06	9.09	0.00	0.30	**0.00**	0.00	0.20	9.09	0.01	0.40	**0.00**	0.04	0.10	9.09	0.05	0.10	9.09	0.06
c07	12.75	0.01	0.90	12.75	0.01	0.60	12.75	0.01	0.90	**6.86**	0.05	0.10	**11.76**	0.06	0.00	**6.86**	0.06
c08	2.91	0.06	0.90	3.15	0.06	0.90	2.95	0.07	0.90	4.32	0.10	0.20	**2.16**	0.11	0.10	**2.55**	0.11
c09	1.84	0.09	0.90	2.55	0.09	0.60	2.55	0.10	0.90	2.97	0.13	0.50	1.84	0.14	0.10	1.98	0.15
c10	1.18	0.19	0.90	1.41	0.19	0.70	1.46	0.20	0.90	1.92	0.23	0.70	**1.01**	0.25	0.10	**0.55**	0.25
c11	12.50	0.01	0.80	15.62	0.01	0.70	15.62	0.01	0.10	15.62	0.06	0.10	15.62	0.08	0.10	**9.38**	0.08
c12	5.74	0.01	0.90	6.52	0.01	0.80	6.52	0.02	0.90	8.70	0.07	0.10	**2.17**	0.09	0.00	**2.17**	0.09
c13	5.43	0.13	0.90	5.43	0.13	0.90	**4.65**	0.13	0.90	5.81	0.18	0.30	**2.71**	0.20	0.10	**2.33**	0.22
c14	2.28	0.19	0.90	4.33	0.19	0.90	4.33	0.20	0.90	5.57	0.24	0.20	2.79	0.27	0.10	**2.17**	0.29
c15	0.66	0.38	0.90	**0.54**	0.39	0.90	0.90	0.39	0.90	0.90	0.42	0.10	**0.18**	0.46	0.10	**0.18**	0.49
c16	18.18	0.02	0.80	18.18	0.02	0.80	18.18	0.02	0.90	18.18	0.17	0.30	18.18	0.20	0.00	18.18	0.20
c17	8.67	0.03	0.90	11.11	0.04	0.90	11.11	0.04	0.90	**5.56**	0.18	0.10	11.11	0.21	0.10	**5.56**	0.21
c18	8.07	0.25	0.90	**7.96**	0.25	0.90	**7.96**	0.26	0.90	**7.96**	0.40	0.40	**7.08**	0.45	0.00	**7.96**	0.46
c19	6.26	0.37	0.90	10.96	0.37	0.90	9.59	0.37	0.90	7.53	0.51	0.10	**4.79**	0.55	0.10	**6.16**	0.60
c20	0.31	0.77	0.90	0.75	0.74	0.90	0.75	0.75	0.90	0.75	0.87	0.20	0.37	0.95	0.10	0.37	1.03
average	5.18	0.13	-	5.38	0.13	-	5.84	0.13	-	**4.99**	0.19	-	**4.75**	0.21	-	**4.03**	0.22
				(0.20)			(0.66)			(-0.19)			(-0.43)			(-1.15)	

Table S3. Results of the benchmark problem set D by using the DNH.

	without centrality		with centrality																	
			degree			eigenvector			closeness			vertex betweenness			edge betweenness					
name	gap	time	α	gap	time	α	gap	time	α	gap	time	α	gap	time	α	gap	time			
d01	0.94	0.01	0.90	0.94	0.01	0.10	0.94	0.01	0.20	6.60	0.15	0.30	0.94	0.18	0.10	0.94	0.19			
d02	7.73	0.02	0.70	**5.45**	0.02	0.10	7.73	0.02	0.70	**5.45**	0.17	0.00	**3.64**	0.20	0.00	**3.64**	0.21			
d03	2.20	0.24	0.70	2.88	0.25	0.40	3.07	0.26	0.90	3.19	0.40	0.70	3.07	0.43	0.10	3.07	0.44			
d04	2.38	0.37	0.90	**1.60**	0.38	0.30	**1.86**	0.39	0.90	**2.07**	0.52	0.60	**1.81**	0.56	0.10	**2.17**	0.57			
d05	0.43	0.74	0.90	0.49	0.76	0.10	**0.40**	0.77	0.90	0.52	0.90	0.60	**0.40**	0.95	0.10	**0.40**	0.96			
d06	11.94	0.01	0.90	**5.97**	0.02	0.20	**5.97**	0.02	0.80	17.91	0.19	0.30	**5.97**	0.23	0.00	**10.45**	0.24			
d07	12.62	0.02	0.70	**1.94**	0.02	0.20	**1.94**	0.03	0.80	**1.94**	0.20	0.10	**1.94**	0.24	0.10	**1.94**	0.24			
d08	3.92	0.45	0.80	**3.73**	0.45	0.90	4.01	0.47	0.90	4.74	0.63	0.60	4.10	0.68	0.10	4.10	0.69			
d09	4.14	0.65	0.90	4.14	0.67	0.60	**3.73**	0.67	0.90	4.70	0.83	0.30	**3.66**	0.87	0.10	**2.83**	0.89			
d10	0.98	1.32	0.90	2.04	1.35	0.30	1.94	1.37	0.90	2.42	1.50	0.80	1.28	1.57	0.20	**0.57**	1.60			
d11	10.34	0.03	0.90	10.34	0.03	0.80	10.34	0.03	0.90	17.24	0.27	0.10	10.34	0.34	0.10	**6.90**	0.35			
d12	2.38	0.06	0.80	4.76	0.06	0.80	4.76	0.06	0.90	4.76	0.30	0.10	**0.00**	0.37	0.10	4.76	0.38			
d13	3.83	0.92	0.90	4.40	0.94	0.80	5.40	0.94	0.90	5.60	1.14	0.20	4.40	1.25	0.10	**3.60**	1.31			
d14	1.87	1.39	0.90	3.93	1.42	0.90	3.15	1.44	0.90	4.65	1.61	0.10	3.30	1.76	0.10	2.55	1.86			
d15	1.82	2.73	0.90	2.15	2.79	0.90	2.15	2.83	0.90	2.06	2.95	0.30	**1.34**	3.16	0.10	**1.25**	3.35			
d16	23.08	0.07	0.80	23.08	0.07	0.80	23.08	0.07	0.90	23.08	0.69	0.30	**15.38**	0.82	0.10	**15.38**	0.85			
d17	8.70	0.12	0.60	13.04	0.09	0.60	13.04	0.10	0.70	13.04	0.72	0.20	8.70	0.89	0.10	**4.35**	0.93			
d18	9.77	1.82	0.90	9.87	1.82	0.90	9.87	1.83	0.90	**8.97**	2.42	0.20	**8.97**	2.64	0.10	**9.42**	2.84			
d19	7.11	2.80	0.90	12.26	2.72	0.90	12.26	2.75	0.90	12.26	3.36	0.30	**6.45**	3.71	0.10	8.71	4.02			
d20	1.07	5.50	0.90	1.49	5.44	0.90	1.49	5.47	0.90	1.49	5.95	0.50	**0.56**	6.51	0.10	**0.56**	7.04			
average	5.86	0.92	-	**5.72**	0.92	-	5.86	0.93	-	7.13	1.19	-	**4.31**	1.30	-	**4.38**	1.38			
				(-0.14)			(0.00)			(1.27)			(-1.55)			(-1.48)				

Table S4. Results of the benchmark problem set E by using the DNH.

	without centrality		with centrality														
			degree			eigenvector			closeness			vertex betweenness			edge betweenness		
name	gap	time	α	gap	time	α	gap	time	α	gap	time	α	gap	time	α	gap	time
e01	12.61	0.04	0.20	**1.80**	0.05	0.10	12.61	0.17	0.00	**6.31**	2.08	0.10	**11.71**	2.39	0.00	12.61	2.46
e02	14.02	0.12	0.70	**7.94**	0.12	0.10	14.02	0.25	0.40	**7.94**	2.25	0.30	**9.81**	2.53	0.60	14.95	2.66
e03	4.60	4.56	0.80	**4.45**	4.57	0.30	**4.24**	4.90	0.90	**4.48**	6.66	0.30	**4.04**	7.23	0.10	**4.04**	7.21
e04	2.20	7.30	0.90	2.20	7.30	0.10	**2.06**	6.88	0.90	2.59	8.75	0.30	**1.63**	9.68	0.50	**1.65**	9.83
e05	0.52	13.67	0.80	**0.48**	13.97	0.10	0.52	13.95	0.90	0.59	15.22	0.60	**0.46**	16.17	0.10	**0.48**	16.38
e06	15.07	0.11	0.60	15.07	0.10	0.10	15.07	0.26	0.90	15.07	2.68	0.10	15.07	3.04	0.10	15.07	3.15
e07	18.62	0.18	0.70	18.62	0.18	0.30	**16.55**	0.33	0.90	18.62	2.72	0.20	**16.55**	3.09	0.10	**11.72**	3.21
e08	4.73	7.99	0.90	4.73	7.99	0.60	**4.47**	8.55	0.90	5.68	10.29	0.60	**3.67**	11.34	0.10	**3.94**	11.51
e09	3.39	12.00	0.90	3.39	12.00	0.60	**3.33**	12.50	0.90	3.87	14.32	0.70	**3.25**	15.43	0.10	**2.58**	15.79
e10	0.98	24.57	0.90	0.98	24.57	0.60	1.02	25.02	0.90	1.68	25.64	0.80	**0.79**	27.19	0.10	**0.71**	28.05
e11	14.71	0.25	0.90	14.71	0.25	0.70	14.71	0.32	0.90	14.71	3.83	0.20	14.71	4.49	0.00	**5.88**	4.68
e12	5.97	0.49	0.90	5.97	0.49	0.80	8.96	0.56	0.70	10.45	3.98	0.10	5.97	4.69	0.10	**4.48**	4.89
e13	5.23	17.09	0.90	5.23	17.09	0.90	5.31	17.82	0.90	7.11	20.02	0.10	**4.53**	23.41	0.10	**4.38**	24.14
e14	4.07	25.19	0.90	4.07	25.19	0.80	4.16	25.72	0.90	4.73	26.40	0.20	**2.71**	32.33	0.10	**2.48**	33.81
e15	1.44	51.18	0.90	1.44	51.18	0.90	1.47	52.92	0.90	1.82	52.07	0.60	**1.04**	60.25	0.10	**0.72**	64.09
e16	33.33	0.50	0.80	33.33	0.46	0.80	33.33	0.53	0.90	33.33	10.38	0.10	**26.67**	11.51	0.10	**20.00**	11.91
e17	16.00	0.89	0.90	16.00	0.89	0.90	16.00	0.97	0.90	**12.00**	10.94	0.10	**8.00**	11.93	0.10	**12.00**	12.40
e18	12.06	32.55	0.90	12.06	32.55	0.90	12.77	32.70	0.90	12.23	41.40	0.30	**8.87**	49.23	0.10	**10.99**	52.67
e19	7.39	41.21	0.90	7.39	41.21	0.90	7.52	40.92	0.90	**7.12**	49.30	0.50	**4.49**	54.56	0.10	**4.88**	59.59
e20	1.71	97.02	0.90	1.71	97.02	0.90	1.71	91.74	0.90	1.71	103.90	0.60	**0.82**	119.27	0.10	**0.89**	135.52
average	8.93	16.85	-	**8.08**	16.86	-	8.99	16.85	-	**8.60**	20.64	-	**7.24**	23.49	-	**6.72**	25.20
				(-0.85)			(0.06)			(-0.33)			(-1.69)			(-2.21)	

Table S5. Results of the benchmark problem set I080 by using the DNH. Texts with a gray background are complete graph instances.

	without centrality		with centrality															
			degree			eigenvector			closeness			vertex betweenness			edge betweenness			
name	gap	time	α	gap	time	α	gap	time	α	gap	time	α	gap	time	α	gap	time	
i080-001	11.58	0.00	0.80	**11.19**	0.00	0.70	11.58	0.00	0.80	**11.19**	0.00	0.00	**11.08**	0.00	0.60	12.09	0.00	
i080-002	11.95	0.00	0.00	11.95	0.00	0.00	11.95	0.00	0.00	11.95	0.00	0.00	**0.00**	0.00	0.10	**5.72**	0.00	
i080-003	5.49	0.00	0.00	5.49	0.00	0.00	**0.00**	0.00	0.20	5.49	0.00	0.00	**0.00**	0.00	0.00	**0.00**	0.00	
i080-004	16.45	0.00	0.00	**12.27**	0.00	0.40	16.45	0.00	0.90	16.45	0.00	0.10	**12.27**	0.00	0.00	**5.68**	0.00	
i080-005	11.40	0.00	0.80	**5.92**	0.00	0.10	**6.37**	0.00	0.50	**5.92**	0.00	0.00	**4.92**	0.00	0.00	**4.92**	0.00	
i080-011	7.17	0.00	0.80	7.23	0.00	0.90	7.17	0.00	0.10	**2.50**	0.00	0.90	7.17	0.00	0.80	7.23	0.00	
i080-012	0.47	0.00	0.60	0.67	0.00	0.90	0.47	0.00	0.50	0.67	0.00	0.60	0.47	0.00	0.80	0.47	0.00	
i080-013	6.81	0.00	0.60	**0.14**	0.00	0.80	6.81	0.00	0.10	**0.51**	0.00	0.00	**0.00**	0.00	0.10	**0.00**	0.00	
i080-014	20.83	0.00	0.90	**13.82**	0.00	0.80	**13.74**	0.00	0.80	**13.74**	0.00	0.80	**13.74**	0.00	0.10	**13.17**	0.00	
i080-015	7.16	0.00	0.00	7.16	0.00	0.00	7.16	0.00	0.00	7.16	0.00	0.80	7.16	0.00	0.80	7.16	0.00	
i080-021	25.19	0.00	0.10	25.19	0.00	0.20	25.19	0.00	0.80	25.19	0.01	0.10	25.19	0.01	0.10	25.19	0.01	
i080-022	25.38	0.00	0.10	25.38	0.00	0.20	25.38	0.00	0.60	25.38	0.01	0.10	25.38	0.01	0.10	25.38	0.01	
i080-023	25.30	0.00	0.10	25.30	0.00	0.30	25.30	0.00	0.40	25.30	0.01	0.10	25.30	0.01	0.10	25.30	0.01	
i080-024	26.87	0.00	0.10	26.87	0.00	0.30	26.87	0.00	0.50	26.87	0.01	0.10	26.87	0.01	0.10	26.87	0.01	
i080-025	27.62	0.00	0.10	27.62	0.00	0.20	27.62	0.00	0.60	27.62	0.01	0.10	27.62	0.01	0.10	27.62	0.01	
i080-031	18.73	0.00	0.80	18.73	0.00	0.10	18.73	0.00	0.90	18.73	0.00	0.80	18.73	0.00	0.10	**6.50**	0.00	
i080-032	12.88	0.00	0.00	**9.00**	0.00	0.00	**5.27**	0.00	0.90	12.88	0.00	0.90	12.88	0.00	0.10	**4.26**	0.00	
i080-033	5.35	0.00	0.10	23.08	0.00	0.10	11.59	0.00	0.80	22.74	0.00	0.70	22.74	0.00	0.00	10.70	0.00	
i080-034	6.93	0.00	0.10	**6.46**	0.00	0.10	**6.46**	0.00	0.00	6.93	0.00	0.10	**5.33**	0.00	0.10	**5.09**	0.00	
i080-035	16.70	0.00	0.00	**10.53**	0.00	0.00	**11.17**	0.00	0.00	16.70	0.00	0.20	**5.91**	0.00	0.10	**5.42**	0.00	
i080-041	17.24	0.00	0.10	**1.57**	0.00	0.20	**1.57**	0.00	0.00	**1.57**	0.00	0.80	17.32	0.00	0.80	17.32	0.00	
i080-042	22.61	0.00	0.80	**21.76**	0.00	0.70	**8.08**	0.00	0.50	22.61	0.00	0.70	**20.20**	0.00	0.00	**18.88**	0.00	
i080-043	21.31	0.00	0.00	**17.61**	0.00	0.20	**16.99**	0.00	0.00	**17.61**	0.00	0.50	**13.13**	0.00	0.80	21.31	0.00	
i080-044	0.81	0.00	0.90	0.81	0.00	0.80	0.81	0.00	0.70	0.81	0.00	0.80	0.81	0.00	0.90	0.81	0.00	
i080-045	18.47	0.00	0.10	**11.22**	0.00	0.10	**6.18**	0.00	0.10	**11.22**	0.00	0.80	**11.22**	0.00	0.80	**11.22**	0.00	
i080-101	7.94	0.00	0.50	7.94	0.00	0.10	**7.78**	0.00	0.80	7.94	0.00	0.60	**0.00**	0.00	0.40	**0.00**	0.00	
i080-102	11.90	0.00	0.00	**4.33**	0.00	0.10	**4.20**	0.00	0.90	**4.20**	0.00	0.10	**3.83**	0.00	0.40	**4.20**	0.00	
i080-103	6.53	0.00	0.60	6.72	0.00	0.40	6.53	0.00	0.80	6.53	0.00	0.20	6.53	0.00	0.00	**2.96**	0.00	
i080-104	7.84	0.00	0.70	**7.72**	0.00	0.10	**7.72**	0.00	0.20	**7.72**	0.00	0.10	**7.80**	0.00	0.10	**7.60**	0.00	
i080-105	0.00	0.00	0.00	0.00	0.00	0.00	0.00	0.00	0.00	0.00	0.00	0.00	0.00	0.00	0.40	0.00	0.00	
i080-111	29.64	0.00	0.70	**10.97**	0.00	0.20	**11.07**	0.00	0.40	**0.44**	0.00	0.20	**11.07**	0.00	0.10	**0.15**	0.00	
i080-112	24.67	0.00	0.80	**9.60**	0.00	0.50	**6.21**	0.00	0.70	**14.59**	0.00	0.50	**6.15**	0.00	0.90	**9.76**	0.00	
i080-113	15.98	0.00	0.00	**11.15**	0.00	0.10	**11.15**	0.00	0.00	**11.15**	0.00	0.80	**11.15**	0.00	0.10	**9.71**	0.00	
i080-114	26.28	0.00	0.20	**21.06**	0.00	0.30	**21.06**	0.00	0.00	**10.87**	0.00	0.00	**9.55**	0.00	0.20	**15.09**	0.00	
i080-115	31.75	0.00	0.40	**11.35**	0.00	0.00	**11.35**	0.00	0.50	**11.35**	0.00	0.40	**16.38**	0.00	0.30	**10.44**	0.00	
i080-121	31.58	0.00	0.10	31.58	0.00	0.10	31.58	0.00	0.10	31.58	0.01	0.10	31.58	0.01	0.10	31.58	0.01	
i080-122	31.97	0.00	0.10	31.97	0.00	0.30	31.97	0.00	0.80	31.97	0.01	0.10	31.97	0.01	0.10	31.97	0.01	
i080-123	31.36	0.00	0.10	31.36	0.00	0.40	31.36	0.00	0.10	31.36	0.01	0.10	31.36	0.01	0.10	31.36	0.01	
i080-124	30.68	0.00	0.10	30.68	0.00	0.10	30.68	0.00	0.10	30.68	0.01	0.10	30.68	0.01	0.10	30.68	0.01	
i080-125	31.11	0.00	0.10	31.11	0.00	0.20	31.11	0.00	0.80	31.11	0.01	0.10	31.11	0.01	0.10	31.11	0.01	
i080-131	8.01	0.00	0.80	**3.81**	0.00	0.10	8.01	0.00	0.40	**3.81**	0.00	0.50	**4.03**	0.00	0.70	8.01	0.00	
i080-132	8.90	0.00	0.00	9.50	0.00	0.80	8.90	0.00	0.50	9.50	0.00	0.20	**5.14**	0.00	0.10	**4.72**	0.00	
i080-133	14.02	0.00	0.10	**0.84**	0.00	0.30	**0.84**	0.00	0.30	**0.84**	0.00	0.40	**0.00**	0.00	0.50	**0.00**	0.00	
i080-134	6.62	0.00	0.00	6.62	0.00	0.00	6.62	0.00	0.60	6.62	0.00	0.00	**5.36**	0.00	0.60	6.62	0.00	
i080-135	3.81	0.00	0.20	3.81	0.00	0.10	3.81	0.00	0.00	3.81	0.00	0.80	3.81	0.00	0.70	3.81	0.00	
i080-141	15.10	0.00	0.80	**5.59**	0.00	0.60	**5.59**	0.00	0.40	**5.59**	0.00	0.20	**9.68**	0.00	0.80	15.10	0.00	
i080-142	25.31	0.00	0.20	**16.16**	0.00	0.20	**5.04**	0.00	0.40	**16.16**	0.00	0.70	**18.85**	0.00	0.10	**17.68**	0.00	
i080-143	16.19	0.00	0.60	**0.74**	0.00	0.20	**0.74**	0.00	0.00	**1.47**	0.00	0.30	**5.72**	0.00	0.80	16.24	0.00	
i080-144	22.57	0.00	0.50	**14.00**	0.00	0.20	**12.92**	0.00	0.40	**12.98**	0.00	0.00	**12.36**	0.00	0.30	**21.33**	0.00	
i080-145	22.64	0.00	0.00	**2.67**	0.00	0.30	**7.55**	0.00	0.00	**2.67**	0.00	0.00	**2.67**	0.00	0.50	**14.76**	0.00	

Table S5. (Continued) Results of the benchmark problem set I080 by using the DNH. Texts with a gray background are complete graph instances.

	without		with centrality																
	centrality			degree			eigenvector			closeness			vertex betweenness			edge betweenness	
name	gap	time	α	gap	time	α	gap	time	α	gap	time	α	gap	time	α	gap	time
i080-201	4.20	0.00	0.50	4.20	0.00	0.30	4.20	0.00	0.10	4.20	0.00	0.30	4.20	0.00	0.20	4.20	0.00
i080-202	6.52	0.00	0.20	**2.54**	0.00	0.10	**0.56**	0.00	0.20	**2.54**	0.00	0.90	**2.65**	0.00	0.70	**0.52**	0.00
i080-203	14.98	0.00	0.00	**6.57**	0.00	0.10	**10.83**	0.00	0.00	**6.57**	0.00	0.00	**4.35**	0.00	0.00	**4.41**	0.00
i080-204	5.88	0.00	0.90	5.97	0.00	0.80	**3.54**	0.00	0.60	**3.58**	0.00	0.20	**3.54**	0.00	0.50	**3.54**	0.00
i080-205	8.37	0.00	0.90	**4.16**	0.00	0.20	**4.54**	0.00	0.80	**4.18**	0.00	0.80	**4.16**	0.00	0.70	**4.23**	0.00
i080-211	17.87	0.00	0.80	**6.77**	0.00	0.60	**6.55**	0.00	0.90	**6.33**	0.00	0.80	**6.33**	0.00	0.50	**10.66**	0.00
i080-212	20.34	0.00	0.80	**15.61**	0.00	0.90	**12.40**	0.00	0.90	**15.56**	0.00	0.00	**11.78**	0.00	0.00	**10.72**	0.00
i080-213	16.67	0.00	0.70	**14.46**	0.00	0.80	**14.38**	0.00	0.90	16.69	0.00	0.20	**4.16**	0.00	0.60	**4.05**	0.00
i080-214	22.68	0.00	0.90	22.68	0.00	0.70	22.68	0.00	0.30	**20.19**	0.00	0.10	**5.38**	0.00	0.00	**4.58**	0.00
i080-215	15.54	0.00	0.20	**8.58**	0.00	0.20	**8.42**	0.00	0.50	**13.26**	0.00	0.30	**12.33**	0.00	0.80	15.54	0.00
i080-221	38.89	0.00	0.10	38.89	0.00	0.40	38.89	0.00	0.10	38.89	0.01	0.10	38.89	0.01	0.10	38.89	0.01
i080-222	38.81	0.00	0.10	38.81	0.00	0.40	38.81	0.00	0.10	38.81	0.01	0.10	38.81	0.01	0.10	38.81	0.01
i080-223	39.01	0.00	0.10	39.01	0.00	0.30	39.01	0.00	0.10	39.01	0.01	0.10	39.01	0.01	0.10	39.01	0.01
i080-224	38.46	0.00	0.10	38.46	0.00	0.50	38.46	0.00	0.10	38.46	0.01	0.10	38.46	0.01	0.10	38.46	0.01
i080-225	38.44	0.00	0.10	38.44	0.00	0.50	38.44	0.00	0.10	38.44	0.01	0.10	38.44	0.01	0.10	38.44	0.01
i080-231	20.28	0.00	0.10	**7.40**	0.00	0.10	**4.94**	0.00	0.50	**11.81**	0.00	0.50	**6.78**	0.00	0.00	**8.61**	0.00
i080-232	8.81	0.00	0.80	**6.67**	0.00	0.70	**6.60**	0.00	0.80	**6.69**	0.00	0.50	**6.55**	0.00	0.90	8.88	0.00
i080-233	9.98	0.00	0.50	10.78	0.00	0.90	9.98	0.00	0.10	10.98	0.00	0.00	**8.40**	0.00	0.30	**8.04**	0.00
i080-234	8.73	0.00	0.80	9.12	0.00	0.90	8.73	0.00	0.20	14.04	0.00	0.40	**4.87**	0.00	0.00	**6.79**	0.00
i080-235	7.93	0.00	0.40	**4.41**	0.00	0.20	**6.08**	0.00	0.80	**6.08**	0.00	0.70	**6.40**	0.00	0.90	7.93	0.00
i080-241	26.14	0.00	0.90	26.31	0.00	0.90	26.14	0.00	0.90	26.14	0.00	0.60	**7.55**	0.00	0.20	**16.85**	0.00
i080-242	17.26	0.00	0.50	**16.08**	0.00	0.20	**16.08**	0.00	0.80	17.26	0.00	0.20	**5.49**	0.00	0.10	**12.90**	0.00
i080-243	22.37	0.00	0.20	**14.48**	0.00	0.20	**14.48**	0.00	0.90	22.37	0.00	0.30	**5.07**	0.00	0.70	**17.53**	0.00
i080-244	16.62	0.00	0.70	**11.22**	0.00	0.50	**11.22**	0.00	0.40	**11.22**	0.00	0.30	**9.43**	0.00	0.50	**15.26**	0.00
i080-245	28.50	0.00	0.80	28.58	0.00	0.50	28.55	0.00	0.80	28.53	0.00	0.20	**10.64**	0.00	0.40	**12.43**	0.00
i080-301	9.26	0.00	0.90	9.26	0.00	0.90	9.26	0.00	0.90	9.26	0.00	0.00	**4.13**	0.00	0.40	9.26	0.00
i080-302	6.83	0.00	0.80	6.83	0.00	0.00	**5.59**	0.00	0.70	6.83	0.00	0.10	**3.50**	0.00	0.00	**3.94**	0.00
i080-303	10.30	0.00	0.30	**3.58**	0.00	0.10	**5.30**	0.00	0.20	**5.30**	0.00	0.10	**1.90**	0.00	0.30	**3.74**	0.00
i080-304	7.00	0.00	0.90	7.00	0.00	0.10	**5.62**	0.00	0.80	7.00	0.00	0.60	7.18	0.00	0.70	7.04	0.00
i080-305	15.73	0.00	0.70	**7.45**	0.00	0.50	**10.40**	0.00	0.90	**7.13**	0.00	0.00	**4.70**	0.00	0.00	**4.10**	0.00
i080-311	19.39	0.00	0.90	20.00	0.00	0.90	19.39	0.00	0.90	19.39	0.00	0.60	**7.51**	0.00	0.10	**9.82**	0.00
i080-312	22.17	0.00	0.20	**20.84**	0.00	0.40	**11.54**	0.00	0.90	22.17	0.00	0.40	**10.32**	0.00	0.20	**12.22**	0.00
i080-313	17.01	0.00	0.50	**15.10**	0.00	0.70	**14.66**	0.00	0.50	19.23	0.00	0.60	**8.21**	0.00	0.70	**10.20**	0.00
i080-314	8.75	0.00	0.90	**5.14**	0.00	0.90	**5.05**	0.00	0.00	**8.36**	0.00	0.10	**5.76**	0.00	0.00	**7.40**	0.00
i080-315	16.91	0.00	0.70	**11.91**	0.00	0.70	**11.50**	0.00	0.70	**11.71**	0.00	0.40	**7.51**	0.00	0.10	**10.25**	0.00
i080-321	40.56	0.00	0.10	40.56	0.00	0.40	40.56	0.00	0.10	40.56	0.01	0.10	40.56	0.01	0.10	40.56	0.01
i080-322	39.85	0.00	0.10	39.85	0.00	0.30	39.85	0.00	0.50	39.85	0.01	0.10	39.85	0.01	0.10	39.85	0.01
i080-323	40.02	0.00	0.10	40.02	0.00	0.50	40.02	0.00	0.80	40.02	0.01	0.10	40.02	0.01	0.10	40.02	0.01
i080-324	40.31	0.00	0.10	40.31	0.00	0.30	40.31	0.00	0.10	40.31	0.01	0.10	40.31	0.01	0.10	40.31	0.01
i080-325	41.03	0.00	0.10	41.03	0.00	0.30	41.03	0.00	0.80	41.03	0.01	0.10	41.03	0.01	0.10	41.03	0.01
i080-331	13.61	0.00	0.30	**10.33**	0.00	0.30	**12.13**	0.00	0.00	**9.73**	0.00	0.30	**3.65**	0.00	0.40	**5.07**	0.00
i080-332	17.23	0.00	0.10	**6.08**	0.00	0.10	**13.82**	0.00	0.30	**5.97**	0.00	0.70	**7.91**	0.00	0.20	**9.46**	0.00
i080-333	13.99	0.00	0.50	**10.57**	0.00	0.80	**12.27**	0.00	0.30	**8.59**	0.00	0.40	**7.02**	0.00	0.10	**10.50**	0.00
i080-334	11.72	0.00	0.90	**4.14**	0.00	0.90	11.72	0.00	0.00	12.12	0.00	0.60	**4.22**	0.00	0.00	**8.43**	0.00
i080-335	11.00	0.00	0.70	**7.43**	0.00	0.70	**7.29**	0.00	0.80	**5.83**	0.00	0.10	**5.83**	0.00	0.30	**5.71**	0.00
i080-341	19.31	0.00	0.90	19.38	0.00	0.90	**17.40**	0.00	0.90	19.31	0.00	0.00	**5.69**	0.00	0.10	**11.95**	0.00
i080-342	29.49	0.00	0.90	29.74	0.00	0.90	29.54	0.00	0.90	29.54	0.00	0.00	**5.28**	0.00	0.60	**11.51**	0.00
i080-343	32.85	0.00	0.90	33.33	0.00	0.90	32.95	0.00	0.90	32.88	0.00	0.00	**7.85**	0.00	0.70	**9.33**	0.00
i080-344	31.83	0.00	0.70	32.02	0.00	0.90	31.95	0.00	0.90	31.83	0.00	0.00	**4.59**	0.00	0.20	**4.27**	0.00
i080-345	15.53	0.00	0.90	24.83	0.00	0.90	15.53	0.00	0.90	15.55	0.00	0.70	**9.49**	0.00	0.50	**11.63**	0.00
average	18.58	0.00	-	**15.45** (-3.13)	0.00	-	**15.02** (-3.56)	0.00	-	**15.64** (-2.94)	0.00	-	**12.60** (-5.98)	0.00	-	**13.52** (-5.06)	0.00

Table S6. Results of the benchmark problem set I160 by using the DNH. Texts with a gray background are complete graph instances.

	without centrality		with centrality															
			degree			eigenvector			closeness			vertex betweenness			edge betweenness			
name	gap	time	α	gap	time	α	gap	time	α	gap	time	α	gap	time	α	gap	time	
i160-001	24.22	0.00	0.00	**8.43**	0.00	0.00	**9.12**	0.00	0.40	**12.81**	0.00	0.30	**4.50**	0.00	0.00	**4.22**	0.00	
i160-002	2.49	0.00	0.00	9.59	0.00	0.50	**0.09**	0.00	0.00	9.59	0.00	0.90	**0.09**	0.00	0.40	**0.09**	0.00	
i160-003	8.45	0.00	0.90	9.27	0.00	0.10	8.45	0.00	0.90	8.45	0.00	0.00	**5.92**	0.00	0.00	**5.62**	0.01	
i160-004	5.27	0.00	0.40	5.27	0.00	0.00	5.27	0.00	0.00	5.27	0.00	0.60	5.27	0.00	0.10	5.27	0.00	
i160-005	8.26	0.00	0.60	8.26	0.00	0.80	8.26	0.00	0.40	8.26	0.00	0.70	8.26	0.00	0.00	**8.18**	0.00	
i160-011	29.64	0.00	0.00	**12.88**	0.00	0.10	**12.88**	0.00	0.10	**12.88**	0.01	0.00	**11.69**	0.01	0.10	**16.04**	0.01	
i160-012	24.69	0.00	0.70	24.69	0.00	0.00	**13.89**	0.00	0.40	24.69	0.01	0.20	**6.29**	0.01	0.30	**17.49**	0.01	
i160-013	29.14	0.00	0.30	**24.26**	0.00	0.10	**18.06**	0.00	0.60	**11.86**	0.01	0.80	**11.86**	0.01	0.80	**11.86**	0.01	
i160-014	22.83	0.00	0.80	22.83	0.00	0.50	22.83	0.00	0.10	22.83	0.01	0.80	22.83	0.01	0.20	**18.95**	0.01	
i160-015	28.45	0.00	0.10	**18.89**	0.00	0.20	**18.89**	0.00	0.00	**24.43**	0.01	0.00	**12.44**	0.01	0.40	**17.99**	0.01	
i160-021	30.47	0.01	0.10	30.47	0.01	0.10	30.47	0.01	0.10	30.47	0.04	0.10	30.47	0.04	0.10	30.47	0.04	
i160-022	29.74	0.01	0.10	29.74	0.01	0.20	29.74	0.01	0.10	29.74	0.04	0.10	29.74	0.04	0.10	29.74	0.04	
i160-023	29.90	0.01	0.10	29.90	0.01	0.10	29.90	0.01	0.10	29.90	0.04	0.10	29.90	0.04	0.10	29.90	0.04	
i160-024	28.96	0.01	0.10	28.96	0.01	0.20	28.96	0.01	0.10	28.96	0.04	0.10	28.96	0.04	0.10	28.96	0.04	
i160-025	28.99	0.01	0.10	28.99	0.01	0.40	28.99	0.01	0.10	28.99	0.04	0.10	28.99	0.04	0.10	28.99	0.04	
i160-031	9.35	0.00	0.00	19.12	0.00	0.90	9.35	0.00	0.90	9.35	0.00	0.90	9.45	0.01	0.70	9.35	0.01	
i160-032	6.22	0.00	0.80	6.22	0.00	0.80	6.22	0.00	0.40	6.22	0.00	0.60	6.22	0.01	0.10	**1.42**	0.01	
i160-033	18.94	0.00	0.00	18.94	0.00	0.00	18.94	0.00	0.00	18.94	0.00	0.00	**14.14**	0.01	0.10	**9.71**	0.01	
i160-034	8.83	0.00	0.10	9.70	0.00	0.90	8.83	0.00	0.40	**0.00**	0.00	0.60	9.70	0.01	0.40	**0.00**	0.01	
i160-035	10.08	0.00	0.80	10.08	0.00	0.80	10.08	0.00	0.30	10.08	0.00	0.40	10.08	0.01	0.80	10.08	0.01	
i160-041	17.20	0.00	0.10	**12.78**	0.00	0.30	**12.52**	0.00	0.70	17.20	0.01	0.80	17.20	0.01	0.80	**10.31**	0.01	
i160-042	32.23	0.00	0.30	**13.93**	0.00	0.80	**13.06**	0.00	0.60	**13.06**	0.01	0.10	**10.77**	0.01	0.30	**17.03**	0.01	
i160-043	14.91	0.00	0.90	14.91	0.00	0.70	14.91	0.00	0.10	14.91	0.01	0.40	**12.78**	0.01	0.80	14.91	0.01	
i160-044	12.04	0.00	0.20	22.80	0.00	0.90	12.04	0.00	0.60	12.04	0.01	0.90	12.04	0.01	0.80	12.04	0.01	
i160-045	18.60	0.00	0.90	**6.56**	0.00	0.70	**13.19**	0.00	0.60	**6.69**	0.01	0.80	**6.69**	0.01	0.80	**12.55**	0.01	
i160-101	8.06	0.00	0.10	10.83	0.00	0.40	8.06	0.00	0.90	**5.44**	0.00	0.10	**5.44**	0.01	0.10	**5.44**	0.01	
i160-102	11.13	0.00	0.50	11.13	0.00	0.10	11.13	0.00	0.00	11.13	0.00	0.00	**5.12**	0.01	0.00	**2.72**	0.01	
i160-103	5.99	0.00	0.00	8.60	0.00	0.00	**5.68**	0.00	0.00	11.21	0.00	0.00	6.20	0.01	0.70	5.99	0.01	
i160-104	2.54	0.00	0.00	2.54	0.00	0.20	2.54	0.00	0.00	2.54	0.00	0.30	2.54	0.01	0.70	2.54	0.01	
i160-105	17.01	0.00	0.10	**8.59**	0.00	0.30	**11.45**	0.00	0.50	17.01	0.00	0.30	**8.59**	0.01	0.60	**11.20**	0.01	
i160-111	17.25	0.00	0.20	**11.47**	0.00	0.30	**11.15**	0.00	0.60	**14.29**	0.01	0.00	**11.01**	0.01	0.80	17.25	0.01	
i160-112	19.66	0.00	0.40	**18.30**	0.00	0.00	**16.38**	0.00	0.60	**17.31**	0.01	0.00	**16.59**	0.01	0.10	**15.53**	0.01	
i160-113	26.34	0.00	0.00	**14.24**	0.00	0.30	**20.59**	0.00	0.00	**17.38**	0.01	0.30	**9.07**	0.01	0.00	**12.14**	0.01	
i160-114	18.23	0.00	0.50	**12.41**	0.00	0.10	**16.79**	0.00	0.10	**16.53**	0.01	0.20	**8.80**	0.01	0.70	**17.93**	0.01	
i160-115	13.07	0.00	0.80	**4.32**	0.00	0.50	**4.53**	0.00	0.30	**4.32**	0.01	0.20	**7.46**	0.01	0.90	16.79	0.01	
i160-121	35.84	0.01	0.10	35.84	0.01	0.40	35.84	0.01	0.90	35.84	0.04	0.10	35.84	0.04	0.10	35.84	0.04	
i160-122	36.46	0.01	0.10	36.46	0.01	0.30	36.46	0.01	0.90	36.46	0.04	0.10	36.46	0.04	0.10	36.46	0.04	
i160-123	36.31	0.01	0.10	36.31	0.01	0.30	36.31	0.01	0.80	36.31	0.04	0.10	36.31	0.04	0.10	36.31	0.05	
i160-124	37.76	0.01	0.10	37.76	0.01	0.20	37.76	0.01	0.90	37.76	0.04	0.10	37.76	0.04	0.10	37.76	0.05	
i160-125	36.88	0.01	0.10	36.88	0.01	0.30	36.88	0.01	0.80	36.88	0.04	0.10	36.88	0.04	0.10	36.88	0.04	
i160-131	3.13	0.00	0.90	3.13	0.00	0.70	3.13	0.00	0.90	3.13	0.01	0.90	3.13	0.01	0.90	3.16	0.01	
i160-132	12.09	0.00	0.90	14.99	0.00	0.60	14.99	0.00	0.80	12.09	0.00	0.10	**6.14**	0.01	0.50	**11.88**	0.01	
i160-133	22.12	0.00	0.10	**13.81**	0.00	0.20	**16.85**	0.00	0.10	**16.85**	0.01	0.70	**7.70**	0.01	0.00	**12.86**	0.01	
i160-134	14.15	0.00	0.00	**3.52**	0.00	0.10	**11.56**	0.00	0.00	**3.52**	0.01	0.60	**8.85**	0.01	0.20	**11.33**	0.01	
i160-135	13.46	0.00	0.10	**8.34**	0.00	0.20	**13.29**	0.00	0.00	**10.20**	0.01	0.00	**6.11**	0.01	0.00	**5.81**	0.01	
i160-141	28.09	0.00	0.80	28.25	0.00	0.80	28.09	0.01	0.70	28.09	0.01	0.60	**17.34**	0.01	0.40	**24.13**	0.01	
i160-142	27.83	0.00	0.90	27.95	0.00	0.90	27.83	0.01	0.10	27.83	0.01	0.80	**13.78**	0.01	0.80	**14.95**	0.01	
i160-143	23.00	0.00	0.40	**15.96**	0.00	0.30	**12.63**	0.00	0.10	**10.83**	0.01	0.40	**13.77**	0.01	0.10	**20.41**	0.02	
i160-144	11.66	0.00	0.80	**9.36**	0.00	0.30	13.66	0.00	0.50	**9.40**	0.01	0.40	**11.12**	0.01	0.80	11.66	0.01	
i160-145	17.34	0.00	0.10	**13.23**	0.00	0.50	**11.71**	0.01	0.10	**13.23**	0.01	0.80	17.34	0.01	0.80	17.34	0.01	

Table S6. (Continued) Results of the benchmark problem set I160 by using the DNH. Texts with a gray background are complete graph instances.

	without centrality		with centrality															
			degree			eigenvector			closeness			vertex betweenness			edge betweenness		
name	gap	time	α	gap	time	α	gap	time	α	gap	time	α	gap	time	α	gap	time
i160-201	8.97	0.00	0.40	**7.58**	0.00	0.10	**7.58**	0.00	0.90	**7.58**	0.01	0.50	**7.58**	0.01	0.00	**4.19**	0.01
i160-202	22.08	0.00	0.40	**13.51**	0.00	0.10	**16.20**	0.00	0.00	22.25	0.01	0.30	**10.85**	0.01	0.10	**4.49**	0.01
i160-203	14.65	0.00	0.00	**12.47**	0.00	0.00	**11.25**	0.00	0.00	**13.92**	0.01	0.10	**6.78**	0.01	0.00	**11.50**	0.01
i160-204	9.85	0.00	0.00	10.30	0.00	0.10	10.57	0.00	0.90	9.85	0.01	0.10	**8.77**	0.01	0.90	9.85	0.01
i160-205	14.39	0.00	0.00	**10.28**	0.00	0.10	**7.44**	0.00	0.00	**13.65**	0.01	0.00	**3.13**	0.01	0.00	**4.42**	0.01
i160-211	20.10	0.00	0.70	**12.61**	0.00	0.40	**15.15**	0.00	0.80	**12.50**	0.01	0.20	**10.73**	0.01	0.00	**13.72**	0.01
i160-212	15.10	0.00	0.30	**11.38**	0.00	0.40	**10.63**	0.00	0.10	**10.83**	0.01	0.00	**12.28**	0.01	0.60	**12.64**	0.01
i160-213	18.74	0.00	0.80	**13.48**	0.00	0.70	**12.17**	0.00	0.90	**13.37**	0.01	0.10	**13.53**	0.01	0.80	**13.37**	0.01
i160-214	18.90	0.00	0.20	**14.81**	0.00	0.40	**12.94**	0.00	0.10	**16.96**	0.01	0.00	**12.99**	0.01	0.10	**14.90**	0.01
i160-215	22.80	0.00	0.90	22.89	0.00	0.70	22.80	0.01	0.90	22.80	0.01	0.00	**12.18**	0.01	0.10	**16.46**	0.01
i160-221	41.15	0.01	0.10	41.15	0.01	0.40	41.15	0.02	0.90	41.15	0.05	0.10	41.15	0.05	0.10	41.15	0.05
i160-222	42.09	0.01	0.10	42.09	0.01	0.30	42.09	0.02	0.80	42.09	0.05	0.10	42.09	0.05	0.10	42.09	0.05
i160-223	41.35	0.01	0.10	41.35	0.01	0.40	41.35	0.02	0.10	41.35	0.05	0.10	41.35	0.05	0.10	41.35	0.05
i160-224	41.96	0.01	0.10	41.96	0.01	0.20	41.96	0.02	0.10	41.96	0.05	0.10	41.96	0.05	0.10	41.96	0.05
i160-225	40.67	0.01	0.10	40.67	0.01	0.30	40.67	0.02	0.90	40.67	0.05	0.10	40.67	0.05	0.10	40.67	0.05
i160-231	11.62	0.00	0.70	**10.25**	0.00	0.50	11.62	0.00	0.80	**8.74**	0.01	0.70	11.62	0.01	0.80	11.62	0.01
i160-232	17.93	0.00	0.40	**12.37**	0.00	0.50	**14.78**	0.00	0.90	**14.78**	0.01	0.00	**6.57**	0.01	0.10	**10.25**	0.01
i160-233	18.09	0.00	0.10	**14.18**	0.00	0.70	**15.35**	0.00	0.60	20.22	0.01	0.00	**9.42**	0.01	0.00	**10.25**	0.01
i160-234	7.26	0.00	0.80	**4.56**	0.00	0.50	**6.14**	0.00	0.80	**2.87**	0.01	0.30	**1.49**	0.01	0.30	**1.62**	0.01
i160-235	24.56	0.00	0.40	**13.35**	0.00	0.90	**15.89**	0.00	0.70	**14.52**	0.01	0.00	**13.60**	0.01	0.00	**12.08**	0.01
i160-241	34.17	0.01	0.90	34.29	0.01	0.90	34.25	0.01	0.10	34.17	0.02	0.30	**7.75**	0.02	0.70	**16.54**	0.02
i160-242	33.45	0.01	0.90	33.51	0.01	0.90	33.49	0.01	0.90	33.45	0.02	0.00	**13.05**	0.02	0.70	**20.02**	0.02
i160-243	34.91	0.01	0.90	34.97	0.01	0.90	34.95	0.01	0.90	34.91	0.02	0.20	**4.89**	0.02	0.60	**17.09**	0.02
i160-244	33.59	0.01	0.90	33.87	0.01	0.90	33.59	0.01	0.90	33.59	0.02	0.20	**11.01**	0.02	0.30	**20.06**	0.02
i160-245	33.93	0.01	0.90	34.15	0.01	0.90	34.05	0.01	0.90	33.93	0.02	0.10	**9.74**	0.02	0.10	**15.76**	0.02
i160-301	10.93	0.00	0.50	**10.23**	0.00	0.10	**9.33**	0.00	0.70	10.94	0.01	0.00	**5.76**	0.01	0.10	**5.70**	0.01
i160-302	8.87	0.00	0.00	**7.24**	0.00	0.90	**8.85**	0.00	0.90	9.03	0.01	0.00	**5.63**	0.01	0.20	**8.20**	0.01
i160-303	8.75	0.00	0.10	**3.55**	0.00	0.20	**5.03**	0.00	0.80	**7.18**	0.01	0.10	**2.66**	0.01	0.00	**2.73**	0.01
i160-304	6.27	0.00	0.40	**3.77**	0.00	0.90	**5.46**	0.00	0.90	6.29	0.01	0.20	**3.70**	0.01	0.80	8.01	0.01
i160-305	5.70	0.00	0.90	5.79	0.00	0.60	5.71	0.00	0.80	**5.62**	0.01	0.10	**4.39**	0.01	0.10	**3.14**	0.01
i160-311	23.05	0.01	0.60	**18.61**	0.01	0.50	**18.43**	0.01	0.50	**22.29**	0.01	0.00	**9.81**	0.01	0.10	**12.62**	0.01
i160-312	26.45	0.01	0.20	**22.09**	0.01	0.20	**22.12**	0.01	0.90	26.45	0.01	0.10	**10.03**	0.01	0.20	**9.75**	0.01
i160-313	21.64	0.01	0.80	**21.17**	0.01	0.40	**20.59**	0.01	0.60	**21.17**	0.01	0.10	**8.46**	0.01	0.10	**8.54**	0.01
i160-314	26.40	0.01	0.70	**24.55**	0.01	0.50	**21.33**	0.01	0.00	**26.33**	0.01	0.00	**10.12**	0.01	0.00	**14.07**	0.01
i160-315	24.02	0.01	0.50	**17.72**	0.01	0.70	**22.20**	0.01	0.10	**20.23**	0.01	0.10	**7.61**	0.01	0.30	**11.51**	0.01
i160-321	43.27	0.02	0.10	43.27	0.02	0.40	43.27	0.02	0.10	43.27	0.05	0.10	43.27	0.05	0.10	43.27	0.05
i160-322	43.70	0.02	0.10	43.70	0.02	0.30	43.70	0.02	0.10	43.70	0.05	0.10	43.70	0.05	0.10	43.70	0.05
i160-323	43.38	0.02	0.10	43.38	0.02	0.40	43.38	0.02	0.10	43.38	0.05	0.10	43.38	0.05	0.10	43.38	0.05
i160-324	43.42	0.02	0.10	43.42	0.02	0.50	43.42	0.02	0.10	43.42	0.05	0.10	43.42	0.05	0.10	43.42	0.05
i160-325	43.59	0.02	0.10	43.59	0.02	0.40	43.59	0.02	0.10	43.59	0.05	0.10	43.59	0.05	0.10	43.59	0.05
i160-331	10.58	0.00	0.90	**7.77**	0.00	0.60	10.58	0.01	0.90	**7.89**	0.01	0.10	**7.43**	0.01	0.00	**7.54**	0.01
i160-332	15.07	0.00	0.30	**9.32**	0.00	0.10	**13.34**	0.01	0.10	**10.09**	0.01	0.00	**6.20**	0.01	0.00	**7.56**	0.01
i160-333	14.75	0.00	0.70	**7.68**	0.00	0.20	**10.67**	0.01	0.00	**5.86**	0.01	0.00	**4.34**	0.01	0.00	**5.44**	0.01
i160-334	13.08	0.00	0.30	**9.44**	0.00	0.50	**11.49**	0.01	0.80	**5.45**	0.01	0.00	**8.11**	0.01	0.40	**5.75**	0.01
i160-335	12.96	0.00	0.50	**6.40**	0.00	0.50	**11.29**	0.00	0.70	**6.84**	0.01	0.00	**4.89**	0.01	0.10	**7.15**	0.01
i160-341	37.13	0.01	0.90	37.28	0.01	0.90	37.17	0.01	0.90	37.13	0.02	0.10	**9.51**	0.02	0.70	**18.38**	0.02
i160-342	37.28	0.01	0.90	37.49	0.01	0.90	37.43	0.01	0.90	37.28	0.02	0.10	**5.21**	0.02	0.60	**17.98**	0.02
i160-343	38.82	0.01	0.90	38.92	0.01	0.90	38.89	0.01	0.90	38.83	0.02	0.20	**6.24**	0.02	0.40	**17.00**	0.02
i160-344	38.00	0.01	0.90	38.24	0.01	0.90	38.03	0.01	0.80	38.00	0.02	0.10	**5.27**	0.02	0.20	**20.22**	0.02
i160-345	37.38	0.01	0.90	37.48	0.01	0.90	37.43	0.01	0.80	37.38	0.02	0.10	**6.79**	0.02	0.40	**16.15**	0.02
average	22.32	0.00	-	**19.96** (-2.36)	0.01	-	**20.01** (-2.31)	0.01	-	**20.13** (-2.19)	0.02	-	**14.60** (-7.72)	0.02	-	**16.48** (-5.84)	0.02

Table S7. Results of the benchmark problem set I320 by using the DNH. Texts with a gray background are complete graph instances.

	without centrality		with centrality															
			degree			eigenvector			closeness			vertex betweenness			edge betweenness		
name	gap	time	α	gap	time	α	gap	time	α	gap	time	α	gap	time	α	gap	time
i320-001	11.94	0.00	0.00	12.39	0.00	0.10	11.94	0.00	0.90	11.94	0.02	0.10	**4.30**	0.02	0.10	**11.38**	0.02
i320-002	8.04	0.00	0.20	**7.80**	0.00	0.30	**7.80**	0.00	0.50	8.04	0.02	0.10	**4.36**	0.02	0.40	**1.72**	0.02
i320-003	22.07	0.00	0.00	**20.12**	0.00	0.00	**20.05**	0.00	0.60	22.07	0.02	0.00	**10.03**	0.02	0.20	**12.99**	0.02
i320-004	14.22	0.00	0.70	**7.26**	0.00	0.10	**7.26**	0.00	0.90	**7.26**	0.02	0.20	**7.06**	0.02	0.30	**7.06**	0.02
i320-005	17.02	0.00	0.10	**14.41**	0.00	0.10	**14.41**	0.00	0.00	**14.41**	0.02	0.10	**14.41**	0.02	0.30	**10.36**	0.02
i320-011	17.95	0.00	0.80	20.26	0.01	0.50	19.78	0.01	0.10	20.26	0.03	0.00	**15.78**	0.03	0.00	**8.57**	0.03
i320-012	27.54	0.00	0.00	**19.13**	0.00	0.20	**19.13**	0.01	0.00	**9.35**	0.03	0.20	**5.16**	0.03	0.10	**23.54**	0.03
i320-013	24.57	0.00	0.00	**16.65**	0.00	0.00	**16.65**	0.01	0.10	**17.23**	0.03	0.80	24.57	0.03	0.80	24.57	0.03
i320-014	15.24	0.00	0.40	**11.74**	0.00	0.60	**6.02**	0.01	0.70	**6.02**	0.03	0.80	15.24	0.03	0.10	**15.14**	0.03
i320-015	34.48	0.00	0.10	**20.79**	0.00	0.60	**20.79**	0.01	0.70	34.48	0.03	0.00	**16.22**	0.03	0.00	**26.81**	0.03
i320-021	31.68	0.03	0.10	31.68	0.04	0.10	31.68	0.04	0.10	31.68	0.31	0.10	31.68	0.29	0.10	31.68	0.32
i320-022	31.57	0.03	0.10	31.57	0.04	0.20	31.57	0.04	0.10	31.57	0.30	0.10	31.57	0.30	0.10	31.57	0.31
i320-023	33.70	0.03	0.10	33.70	0.04	0.10	33.70	0.04	0.10	33.70	0.30	0.10	33.70	0.29	0.10	33.70	0.31
i320-024	31.81	0.03	0.10	31.81	0.04	0.40	31.81	0.04	0.80	31.81	0.30	0.10	31.81	0.30	0.10	31.81	0.32
i320-025	32.00	0.03	0.10	32.00	0.04	0.20	32.00	0.04	0.10	32.00	0.31	0.10	32.00	0.29	0.10	32.00	0.31
i320-031	22.71	0.00	0.00	**5.42**	0.00	0.10	**9.13**	0.00	0.90	**15.45**	0.02	0.10	**8.45**	0.02	0.20	**8.45**	0.02
i320-032	8.19	0.00	0.90	8.27	0.00	0.90	8.19	0.00	0.10	8.38	0.02	0.20	8.19	0.02	0.00	**3.43**	0.02
i320-033	15.96	0.00	0.60	15.96	0.00	0.40	15.96	0.00	0.90	15.96	0.02	0.60	15.96	0.02	0.40	**15.46**	0.02
i320-034	19.83	0.00	0.00	**7.77**	0.00	0.10	**15.75**	0.00	0.60	**15.83**	0.02	0.00	**4.09**	0.02	0.00	**11.74**	0.02
i320-035	15.72	0.00	0.80	15.72	0.00	0.60	**12.03**	0.00	0.90	15.72	0.02	0.00	**11.91**	0.02	0.00	**14.47**	0.02
i320-041	9.20	0.01	0.80	9.20	0.01	0.60	9.20	0.01	0.10	**5.16**	0.07	0.90	9.20	0.08	0.90	9.20	0.08
i320-042	34.24	0.01	0.90	**12.01**	0.01	0.70	**12.01**	0.01	0.00	**24.33**	0.07	0.10	**21.94**	0.08	0.40	**31.87**	0.08
i320-043	13.81	0.01	0.80	13.81	0.01	0.50	13.81	0.01	0.40	13.81	0.07	0.80	**6.38**	0.08	0.80	13.81	0.08
i320-044	23.74	0.01	0.90	23.74	0.01	0.70	23.74	0.02	0.70	23.74	0.07	0.10	**19.57**	0.07	0.80	**22.78**	0.08
i320-045	32.09	0.01	0.80	**8.66**	0.01	0.70	**8.66**	0.02	0.40	**9.25**	0.07	0.20	**19.81**	0.08	0.80	**20.82**	0.08
i320-101	14.01	0.00	0.20	**10.71**	0.00	0.10	14.01	0.01	0.90	14.01	0.02	0.10	**12.33**	0.02	0.10	**8.98**	0.02
i320-102	7.29	0.00	0.10	**5.99**	0.00	0.10	**7.22**	0.01	0.10	7.29	0.02	0.10	**5.49**	0.02	0.50	**5.76**	0.02
i320-103	13.16	0.00	0.10	**8.93**	0.00	0.40	**9.97**	0.01	0.80	**9.97**	0.02	0.50	**5.24**	0.02	0.20	**3.59**	0.02
i320-104	21.83	0.00	0.70	**17.04**	0.00	0.20	**14.82**	0.01	0.90	**14.82**	0.02	0.00	**10.24**	0.02	0.00	**7.01**	0.02
i320-105	13.23	0.00	0.10	**5.43**	0.00	0.20	**6.93**	0.01	0.00	**6.92**	0.02	0.10	**5.30**	0.02	0.10	**6.68**	0.02
i320-111	31.94	0.01	0.80	**12.12**	0.01	0.80	**9.83**	0.01	0.10	**14.77**	0.03	0.00	**15.02**	0.03	0.50	**27.66**	0.03
i320-112	25.92	0.01	0.80	**15.45**	0.01	0.70	**17.26**	0.01	0.00	**16.41**	0.03	0.10	**19.51**	0.03	0.10	**20.77**	0.03
i320-113	21.43	0.01	0.70	**12.68**	0.01	0.10	**12.96**	0.01	0.10	**7.82**	0.03	0.40	**17.81**	0.03	0.80	21.43	0.03
i320-114	19.10	0.01	0.80	**7.41**	0.01	0.80	**12.33**	0.01	0.60	**11.94**	0.03	0.30	**16.74**	0.03	0.80	**14.57**	0.03
i320-115	20.58	0.01	0.90	**18.74**	0.01	0.30	23.97	0.01	0.90	20.86	0.03	0.80	20.58	0.03	0.80	20.58	0.03
i320-121	39.51	0.04	0.10	39.51	0.05	0.30	39.51	0.05	0.10	39.51	0.31	0.10	39.51	0.31	0.10	39.51	0.32
i320-122	40.98	0.04	0.10	40.98	0.05	0.20	40.98	0.05	0.10	40.98	0.31	0.10	40.98	0.30	0.10	40.98	0.33
i320-123	39.47	0.04	0.10	39.47	0.05	0.10	39.47	0.05	0.10	39.47	0.31	0.10	39.47	0.30	0.10	39.47	0.32
i320-124	40.02	0.05	0.10	40.02	0.05	0.30	40.02	0.06	0.10	40.02	0.31	0.10	40.02	0.31	0.10	40.02	0.32
i320-125	39.70	0.04	0.10	39.70	0.05	0.20	39.70	0.05	0.10	39.70	0.31	0.10	39.70	0.30	0.10	39.70	0.32
i320-131	18.71	0.00	0.50	**15.55**	0.00	0.70	**15.11**	0.01	0.50	**11.74**	0.02	0.60	**9.67**	0.02	0.40	**9.67**	0.02
i320-132	20.92	0.00	0.90	20.98	0.00	0.70	20.92	0.01	0.60	**13.84**	0.02	0.50	20.98	0.02	0.20	**9.90**	0.02
i320-133	8.18	0.00	0.00	8.25	0.01	0.40	8.18	0.01	0.00	8.25	0.02	0.90	8.18	0.02	0.30	**2.40**	0.02
i320-134	20.31	0.01	0.00	**13.92**	0.01	0.20	**18.21**	0.01	0.00	**16.24**	0.02	0.00	**11.17**	0.02	0.00	**5.65**	0.02
i320-135	20.05	0.00	0.60	**15.14**	0.00	0.60	**14.86**	0.01	0.70	**14.92**	0.02	0.50	**13.87**	0.02	0.40	**11.61**	0.02
i320-141	32.09	0.02	0.90	32.42	0.02	0.90	32.20	0.02	0.90	32.20	0.08	0.20	**19.02**	0.08	0.50	**21.24**	0.09
i320-142	33.19	0.02	0.90	33.25	0.02	0.80	33.25	0.02	0.90	33.19	0.08	0.10	**12.78**	0.08	0.80	33.19	0.09
i320-143	30.33	0.02	0.90	**29.99**	0.02	0.70	**16.71**	0.02	0.40	**30.13**	0.08	0.00	**11.23**	0.08	0.40	**25.72**	0.09
i320-144	34.82	0.02	0.90	34.88	0.02	0.90	34.82	0.02	0.90	34.82	0.08	0.10	**8.17**	0.08	0.50	**19.70**	0.09
i320-145	31.85	0.02	0.90	32.10	0.02	0.90	31.91	0.03	0.90	31.88	0.08	0.10	**8.00**	0.08	0.50	**16.58**	0.09

Table S7. (Continued) Results of the benchmark problem set I320 by using the DNH. Texts with a gray background are complete graph instances.

	without centrality		with centrality															
			degree			eigenvector			closeness			vertex betweenness			edge betweenness		
name	gap	time	α	gap	time	α	gap	time	α	gap	time	α	gap	time	α	gap	time
i320-201	12.95	0.01	0.80	**7.60**	0.01	0.30	**9.23**	0.01	0.80	**10.15**	0.02	0.10	**6.47**	0.03	0.10	**7.29**	0.03
i320-202	12.71	0.01	0.50	**10.18**	0.01	0.10	**11.85**	0.01	0.00	**9.86**	0.02	0.00	**6.71**	0.03	0.10	**8.48**	0.02
i320-203	16.57	0.01	0.00	**8.94**	0.01	0.10	16.57	0.01	0.70	**9.80**	0.02	0.00	**8.04**	0.03	0.00	**8.19**	0.03
i320-204	16.51	0.01	0.00	**7.81**	0.01	0.10	**10.13**	0.01	0.10	**9.40**	0.02	0.10	**7.56**	0.02	0.10	**4.74**	0.03
i320-205	16.19	0.01	0.10	**9.66**	0.01	0.10	**11.61**	0.01	0.10	**10.04**	0.02	0.00	**11.19**	0.02	0.00	**8.35**	0.03
i320-211	24.36	0.02	0.80	**17.30**	0.02	0.40	**18.20**	0.02	0.40	**11.10**	0.04	0.20	**14.79**	0.04	0.20	**15.24**	0.04
i320-212	19.06	0.02	0.50	**10.90**	0.01	0.90	**15.44**	0.02	0.00	**13.80**	0.04	0.00	**14.15**	0.04	0.10	**16.63**	0.04
i320-213	21.04	0.02	0.80	**16.82**	0.02	0.40	**16.08**	0.02	0.10	**14.17**	0.04	0.00	**13.17**	0.04	0.20	**18.76**	0.04
i320-214	16.26	0.02	0.70	**15.26**	0.02	0.10	**12.76**	0.02	0.60	**15.40**	0.04	0.10	**11.72**	0.04	0.40	**11.56**	0.04
i320-215	23.63	0.02	0.10	**15.17**	0.02	0.50	**13.64**	0.02	0.00	**17.60**	0.04	0.00	**16.19**	0.04	0.00	**17.80**	0.05
i320-221	42.91	0.06	0.10	42.91	0.07	0.30	42.91	0.07	0.10	42.91	0.33	0.10	42.91	0.32	0.10	42.91	0.34
i320-222	42.88	0.07	0.10	42.88	0.07	0.20	42.88	0.07	0.10	42.88	0.33	0.10	42.88	0.32	0.10	42.88	0.34
i320-223	42.63	0.06	0.10	42.63	0.07	0.30	42.63	0.07	0.10	42.63	0.33	0.10	42.63	0.32	0.10	42.63	0.34
i320-224	42.65	0.06	0.10	42.65	0.07	0.40	42.65	0.07	0.10	42.65	0.33	0.10	42.65	0.32	0.10	42.65	0.34
i320-225	42.94	0.06	0.10	42.94	0.07	0.20	42.94	0.07	0.10	42.94	0.33	0.10	42.94	0.32	0.10	42.94	0.34
i320-231	11.57	0.01	0.00	**7.68**	0.01	0.30	**8.22**	0.01	0.00	**8.80**	0.03	0.00	**9.89**	0.03	0.70	**10.65**	0.03
i320-232	15.59	0.01	0.20	**7.13**	0.01	0.80	**13.78**	0.01	0.70	**12.14**	0.03	0.20	**9.99**	0.03	0.30	**11.87**	0.03
i320-233	19.47	0.01	0.10	**15.79**	0.01	0.10	**19.00**	0.01	0.10	19.97	0.02	0.00	**10.96**	0.03	0.10	**12.49**	0.03
i320-234	18.09	0.01	0.10	**12.93**	0.01	0.70	**14.97**	0.01	0.00	**11.56**	0.02	0.30	**10.51**	0.03	0.00	**9.74**	0.03
i320-235	9.79	0.01	0.90	9.88	0.01	0.30	**9.01**	0.01	0.90	9.79	0.02	0.20	**9.38**	0.03	0.30	**7.81**	0.03
i320-241	37.77	0.04	0.90	37.88	0.04	0.90	37.77	0.04	0.10	37.77	0.09	0.10	**10.54**	0.10	0.70	**25.43**	0.10
i320-242	37.60	0.04	0.90	37.71	0.04	0.90	37.64	0.04	0.10	37.60	0.09	0.00	**9.44**	0.09	0.70	**23.80**	0.10
i320-243	37.31	0.03	0.90	37.44	0.04	0.90	37.31	0.04	0.10	37.31	0.09	0.00	**10.69**	0.09	0.30	**23.77**	0.11
i320-244	37.21	0.03	0.90	37.27	0.04	0.90	37.21	0.04	0.10	37.21	0.09	0.00	**13.92**	0.09	0.70	**21.48**	0.10
i320-245	37.87	0.03	0.90	37.92	0.04	0.90	37.87	0.04	0.10	37.87	0.09	0.00	**11.74**	0.09	0.50	**21.84**	0.10
i320-301	10.70	0.02	0.90	**9.50**	0.02	0.10	**7.17**	0.02	0.80	**8.66**	0.03	0.10	**7.65**	0.03	0.00	**8.30**	0.04
i320-302	8.41	0.02	0.90	**6.47**	0.02	0.60	8.41	0.02	0.20	**7.88**	0.03	0.50	**6.48**	0.03	0.50	**5.27**	0.03
i320-303	5.15	0.02	0.80	6.10	0.02	0.10	**5.11**	0.02	0.90	5.57	0.03	0.10	**4.79**	0.04	0.50	**4.79**	0.04
i320-304	8.99	0.02	0.40	**3.28**	0.02	0.30	**6.87**	0.02	0.10	**3.21**	0.03	0.10	**2.46**	0.04	0.10	**2.06**	0.04
i320-305	9.26	0.02	0.80	**5.33**	0.02	0.10	**6.36**	0.02	0.10	**6.24**	0.03	0.00	**3.47**	0.04	0.00	**4.22**	0.04
i320-311	23.82	0.04	0.90	**22.94**	0.04	0.90	23.91	0.04	0.80	24.01	0.06	0.00	**11.31**	0.07	0.10	**11.46**	0.06
i320-312	29.81	0.04	0.90	**28.34**	0.04	0.80	**27.19**	0.04	0.90	**29.32**	0.06	0.00	**11.05**	0.07	0.20	**13.38**	0.06
i320-313	30.02	0.04	0.70	**28.38**	0.04	0.80	**27.98**	0.04	0.90	**28.38**	0.06	0.00	**10.08**	0.07	0.10	**13.53**	0.06
i320-314	26.28	0.04	0.00	**20.35**	0.04	0.60	**21.77**	0.04	0.60	**22.36**	0.06	0.00	**9.52**	0.07	0.10	**11.21**	0.07
i320-315	30.12	0.04	0.60	**29.67**	0.04	0.70	**28.09**	0.04	0.10	**29.41**	0.06	0.00	**10.96**	0.07	0.50	**12.46**	0.06
i320-321	45.28	0.12	0.10	45.28	0.13	0.30	45.28	0.12	0.10	45.28	0.39	0.10	45.28	0.38	0.10	45.28	0.40
i320-322	45.19	0.12	0.10	45.19	0.12	0.30	45.19	0.12	0.10	45.19	0.38	0.10	45.19	0.38	0.10	45.19	0.39
i320-323	45.41	0.12	0.10	45.41	0.12	0.40	45.41	0.13	0.10	45.41	0.38	0.10	45.41	0.38	0.10	45.41	0.39
i320-324	45.45	0.12	0.10	45.45	0.12	0.30	45.45	0.12	0.10	45.45	0.38	0.10	45.45	0.38	0.10	45.45	0.39
i320-325	45.41	0.11	0.10	45.41	0.12	0.40	45.41	0.12	0.10	45.41	0.38	0.10	45.41	0.37	0.10	45.41	0.39
i320-331	14.35	0.02	0.70	**10.88**	0.02	0.20	**11.84**	0.03	0.80	**10.95**	0.04	0.10	**8.20**	0.04	0.10	**7.20**	0.04
i320-332	11.60	0.02	0.80	**7.42**	0.02	0.80	**9.14**	0.03	0.70	**8.14**	0.04	0.70	**7.00**	0.04	0.50	**6.89**	0.04
i320-333	12.39	0.02	0.20	**10.88**	0.02	0.90	**9.54**	0.03	0.70	**11.17**	0.04	0.00	**10.11**	0.04	0.40	**8.99**	0.04
i320-334	9.74	0.02	0.50	11.14	0.02	0.90	11.09	0.03	0.80	**9.07**	0.04	0.10	**8.97**	0.04	0.80	**9.39**	0.04
i320-335	15.18	0.02	0.80	**10.81**	0.03	0.30	**9.62**	0.03	0.10	**10.07**	0.04	0.00	**8.18**	0.05	0.00	**7.47**	0.05
i320-341	41.45	0.07	0.90	41.59	0.08	0.90	41.46	0.08	0.80	41.46	0.14	0.00	**5.35**	0.12	0.20	**18.67**	0.17
i320-342	42.11	0.08	0.90	42.18	0.08	0.90	42.18	0.08	0.80	42.12	0.14	0.00	**4.70**	0.12	0.10	**16.03**	0.18
i320-343	41.38	0.08	0.90	41.52	0.08	0.90	41.45	0.08	0.90	41.40	0.14	0.00	**5.82**	0.12	0.40	**18.10**	0.15
i320-344	41.59	0.07	0.90	41.83	0.08	0.90	41.69	0.08	0.90	41.64	0.14	0.10	**5.10**	0.12	0.60	**18.13**	0.15
i320-345	41.98	0.07	0.90	42.07	0.08	0.90	42.01	0.08	0.90	41.98	0.14	0.10	**5.03**	0.13	0.70	**19.95**	0.15
average	25.25	0.03	-	**22.05** (-3.20)	0.03	-	**22.40** (-2.85)	0.03	-	**22.46** (-2.79)	0.10	-	**16.58** (-8.67)	0.10	-	**18.91** (-6.34)	0.11

Table S8. Results of the benchmark problem set I640 by using the DNH. Texts with a gray background are complete graph instances.

	without centrality		with centrality															
			degree			eigenvector			closeness			vertex betweenness			edge betweenness			
name	gap	time	α	gap	time	α	gap	time	α	gap	time	α	gap	time	α	gap	time	
i640-001	23.51	0.01	0.50	**14.65**	0.01	0.00	**22.86**	0.05	0.60	**18.79**	0.13	0.40	**11.83**	0.14	0.40	**7.17**	0.15	
i640-002	13.94	0.01	0.00	**6.55**	0.01	0.50	**5.35**	0.05	0.00	**12.52**	0.12	0.70	**5.35**	0.14	0.20	**5.35**	0.14	
i640-003	8.81	0.01	0.90	8.81	0.01	0.10	9.22	0.06	0.80	8.81	0.13	0.10	**5.96**	0.14	0.40	8.81	0.15	
i640-004	14.47	0.01	0.00	**9.45**	0.01	0.10	14.47	0.05	0.10	14.47	0.12	0.20	**8.30**	0.14	0.00	**6.55**	0.15	
i640-005	24.01	0.01	0.00	**9.69**	0.01	0.00	**12.13**	0.04	0.10	**19.27**	0.12	0.20	**7.14**	0.14	0.00	**2.47**	0.15	
i640-011	28.93	0.02	0.00	**28.80**	0.02	0.10	**24.67**	0.06	0.90	28.93	0.22	0.10	**19.06**	0.23	0.20	**19.06**	0.24	
i640-012	20.08	0.03	0.90	20.08	0.03	0.90	20.08	0.06	0.10	20.08	0.21	0.80	20.08	0.24	0.80	20.08	0.24	
i640-013	27.76	0.02	0.70	27.76	0.02	0.20	27.76	0.05	0.10	27.76	0.21	0.80	27.76	0.23	0.80	27.76	0.24	
i640-014	22.85	0.02	0.00	**14.79**	0.02	0.00	**14.79**	0.05	0.10	**14.79**	0.21	0.10	**14.14**	0.22	0.10	**17.60**	0.24	
i640-015	25.95	0.02	0.70	25.95	0.02	0.90	25.95	0.06	0.10	25.95	0.22	0.00	**22.75**	0.24	0.10	**21.01**	0.25	
i640-021	34.36	0.22	0.10	34.36	0.23	0.10	34.36	0.31	0.10	34.36	5.77	0.10	34.36	5.41	0.10	34.36	5.52	
i640-022	33.77	0.21	0.10	33.77	0.21	0.20	33.77	0.28	0.10	33.77	5.39	0.10	33.77	5.07	0.10	33.77	5.05	
i640-023	33.87	0.22	0.10	33.87	0.21	0.10	33.87	0.30	0.10	33.87	5.79	0.10	33.87	5.42	0.10	33.87	5.55	
i640-024	33.87	0.22	0.10	33.87	0.22	0.40	33.87	0.31	0.10	33.87	5.71	0.10	33.87	5.44	0.10	33.87	5.59	
i640-025	34.73	0.21	0.10	34.73	0.21	0.20	34.73	0.29	0.10	34.73	5.66	0.10	34.73	5.35	0.10	34.73	5.50	
i640-031	32.25	0.01	0.10	**27.30**	0.01	0.10	**21.20**	0.07	0.00	**12.14**	0.14	0.70	**25.69**	0.16	0.40	**20.56**	0.16	
i640-032	21.21	0.01	0.00	**15.81**	0.01	0.10	**18.14**	0.05	0.00	**15.22**	0.14	0.00	**11.83**	0.16	0.10	**5.11**	0.16	
i640-033	6.07	0.01	0.70	6.07	0.01	0.30	**2.73**	0.07	0.80	6.07	0.14	0.10	**3.44**	0.16	0.00	**0.46**	0.16	
i640-034	21.00	0.01	0.40	**14.56**	0.01	0.70	21.00	0.05	0.00	**14.49**	0.14	0.10	**17.34**	0.15	0.70	**14.49**	0.16	
i640-035	6.26	0.01	0.70	6.26	0.01	0.10	12.52	0.08	0.90	6.26	0.14	0.30	6.26	0.16	0.10	6.96	0.16	
i640-041	29.15	0.08	0.10	**19.24**	0.06	0.30	**15.71**	0.09	0.00	**11.11**	1.08	0.10	**22.51**	1.03	0.80	**29.05**	1.07	
i640-042	21.04	0.08	0.60	**8.27**	0.06	0.60	**6.57**	0.10	0.10	**15.67**	1.09	0.40	**18.51**	1.03	0.50	23.42	1.08	
i640-043	24.96	0.08	0.00	24.96	0.06	0.00	24.96	0.08	0.00	24.96	1.05	0.40	**23.67**	1.03	0.80	24.96	1.06	
i640-044	20.54	0.07	0.80	**5.68**	0.08	0.50	**5.68**	0.10	0.70	20.54	1.07	0.70	**17.80**	1.05	0.80	20.54	1.08	
i640-045	28.24	0.07	0.20	**12.33**	0.06	0.50	**14.79**	0.10	0.10	**18.22**	1.06	0.60	**18.11**	1.02	0.70	**28.08**	1.07	
i640-101	18.13	0.02	0.90	18.13	0.03	0.50	**18.09**	0.08	0.90	**18.09**	0.14	0.00	**16.43**	0.16	0.00	**11.18**	0.17	
i640-102	13.02	0.02	0.80	**11.14**	0.02	0.80	**8.69**	0.07	0.20	**12.07**	0.14	0.80	**8.73**	0.16	0.10	**8.85**	0.17	
i640-103	12.63	0.02	0.90	12.63	0.02	0.80	**12.54**	0.07	0.00	12.63	0.14	0.10	**12.33**	0.16	0.20	**10.23**	0.16	
i640-104	13.82	0.03	0.90	13.82	0.03	0.20	**12.89**	0.09	0.20	**13.11**	0.14	0.00	**9.26**	0.16	0.00	**7.72**	0.17	
i640-105	15.48	0.02	0.20	**10.40**	0.03	0.10	19.45	0.09	0.00	**10.35**	0.14	0.30	**10.57**	0.16	0.20	**10.18**	0.16	
i640-111	26.03	0.06	0.50	**20.76**	0.06	0.50	**15.73**	0.10	0.20	**21.01**	0.26	0.20	**23.40**	0.27	0.00	**20.38**	0.29	
i640-112	35.91	0.06	0.30	**21.34**	0.06	0.20	**19.96**	0.10	0.00	**28.08**	0.26	0.00	**25.74**	0.28	0.00	**25.30**	0.30	
i640-113	26.84	0.06	0.60	**24.50**	0.06	0.20	**17.47**	0.10	0.00	**25.79**	0.26	0.00	**26.74**	0.28	0.80	29.73	0.27	
i640-114	23.29	0.06	0.80	**22.73**	0.06	0.60	**22.73**	0.09	0.70	**22.12**	0.25	0.80	23.35	0.27	0.10	**21.59**	0.28	
i640-115	19.53	0.06	0.90	19.53	0.06	0.90	**17.73**	0.10	0.00	**19.27**	0.25	0.70	**17.31**	0.27	0.80	**17.31**	0.28	
i640-121	42.13	0.33	0.10	42.13	0.32	0.20	42.13	0.40	0.10	42.13	5.65	0.10	42.13	5.32	0.10	42.13	5.42	
i640-122	42.23	0.32	0.10	42.23	0.33	0.20	42.23	0.41	0.10	42.23	5.59	0.10	42.23	5.20	0.10	42.23	5.43	
i640-123	41.50	0.33	0.10	41.50	0.33	0.20	41.50	0.40	0.10	41.50	5.51	0.10	41.50	5.19	0.10	41.50	5.22	
i640-124	41.93	0.31	0.10	41.93	0.31	0.20	41.93	0.39	0.10	41.93	5.48	0.10	41.93	5.14	0.10	41.93	5.20	
i640-125	41.93	0.28	0.10	41.93	0.27	0.20	41.93	0.41	0.10	41.93	5.76	0.10	41.93	5.46	0.10	41.93	5.37	
i640-131	15.85	0.03	0.40	**14.49**	0.03	0.30	16.99	0.08	0.80	**12.65**	0.15	0.10	**10.20**	0.18	0.20	**8.32**	0.19	
i640-132	13.97	0.03	0.90	13.97	0.03	0.80	**11.32**	0.07	0.90	13.97	0.16	0.80	13.97	0.18	0.90	**13.91**	0.18	
i640-133	15.23	0.03	0.70	**14.61**	0.04	0.10	**12.09**	0.09	0.10	**14.19**	0.16	0.10	**14.97**	0.20	0.40	**10.47**	0.19	
i640-134	15.21	0.03	0.90	15.21	0.03	0.20	**10.18**	0.07	0.80	17.64	0.16	0.90	15.21	0.18	0.50	**7.63**	0.19	
i640-135	16.98	0.03	0.60	**13.71**	0.03	0.50	**13.18**	0.08	0.10	**16.06**	0.16	0.00	**12.07**	0.19	0.80	**14.38**	0.18	
i640-141	36.39	0.17	0.90	36.39	0.17	0.90	**36.37**	0.20	0.10	**36.37**	1.18	0.80	**14.00**	1.13	0.80	**14.00**	1.19	
i640-142	36.40	0.16	0.90	36.40	0.16	0.90	**36.24**	0.20	0.10	**36.24**	1.19	0.00	**15.93**	1.09	0.30	**26.63**	1.21	
i640-143	36.54	0.15	0.90	36.54	0.15	0.90	**36.48**	0.19	0.10	**36.48**	1.17	0.00	**12.80**	1.07	0.80	**26.59**	1.15	
i640-144	36.33	0.16	0.90	36.33	0.16	0.90	**36.29**	0.19	0.10	**36.29**	1.18	0.00	**15.00**	1.07	0.70	**24.88**	1.17	
i640-145	35.49	0.16	0.80	35.49	0.15	0.70	**35.47**	0.19	0.10	**35.45**	1.19	0.20	**13.51**	1.14	0.70	**24.72**	1.19	

Table S8. (Continued) Results of the benchmark problem set I640 by using the DNH. Texts with a gray background are complete graph instances.

	without centrality		with centrality															
			degree			eigenvector			closeness			vertex betweenness			edge betweenness		
name	gap	time	α	gap	time	α	gap	time	α	gap	time	α	gap	time	α	gap	time
i640-201	11.18	0.05	0.50	**10.65**	0.06	0.30	12.89	0.10	0.90	11.80	0.17	0.00	**4.39**	0.20	0.00	**7.38**	0.20
i640-202	13.51	0.05	0.80	**12.97**	0.05	0.10	15.85	0.10	0.10	**13.01**	0.17	0.10	**10.65**	0.20	0.20	**10.78**	0.19
i640-203	18.96	0.05	0.80	**17.43**	0.05	0.30	**17.65**	0.10	0.00	**15.62**	0.17	0.00	**12.98**	0.19	0.10	**11.49**	0.20
i640-204	12.88	0.05	0.10	**8.54**	0.06	0.20	13.41	0.12	0.00	**11.91**	0.17	0.00	**10.54**	0.19	0.00	**12.53**	0.20
i640-205	11.64	0.05	0.30	**10.51**	0.06	0.30	**11.61**	0.11	0.10	**11.63**	0.17	0.10	**9.28**	0.19	0.30	**8.43**	0.19
i640-211	20.10	0.12	0.40	**17.70**	0.11	0.20	**17.08**	0.15	0.10	20.98	0.30	0.10	**20.09**	0.33	0.10	**14.02**	0.33
i640-212	25.36	0.12	0.70	**21.49**	0.11	0.60	**18.19**	0.14	0.80	**20.75**	0.31	0.00	**24.42**	0.34	0.80	**24.68**	0.34
i640-213	21.56	0.12	0.50	**19.55**	0.11	0.40	**13.48**	0.14	0.00	**20.57**	0.29	0.00	**18.60**	0.35	0.80	**21.31**	0.34
i640-214	19.76	0.12	0.80	**17.77**	0.11	0.70	**18.36**	0.15	0.30	**19.68**	0.31	0.00	**14.00**	0.35	0.80	**18.42**	0.34
i640-215	22.16	0.12	0.70	**20.11**	0.12	0.40	**17.28**	0.15	0.90	**21.14**	0.31	0.50	**20.10**	0.33	0.90	**20.18**	0.34
i640-221	44.07	0.52	0.10	44.07	0.52	0.30	44.07	0.57	0.10	44.07	5.79	0.10	44.07	5.40	0.10	44.07	5.53
i640-222	44.36	0.45	0.10	44.36	0.45	0.30	44.36	0.57	0.10	44.36	5.79	0.10	44.36	5.44	0.10	44.36	5.48
i640-223	44.15	0.52	0.10	44.15	0.52	0.20	44.15	0.58	0.10	44.15	5.77	0.10	44.15	5.41	0.10	44.15	5.56
i640-224	44.41	0.50	0.10	44.41	0.50	0.30	44.41	0.56	0.10	44.41	5.67	0.10	44.41	5.30	0.10	44.41	5.34
i640-225	44.18	0.50	0.10	44.18	0.50	0.20	44.18	0.56	0.10	44.18	5.70	0.10	44.18	5.35	0.10	44.18	5.40
i640-231	15.62	0.05	0.90	15.62	0.05	0.70	17.52	0.11	0.10	**15.11**	0.19	0.00	16.16	0.22	0.20	**15.11**	0.21
i640-232	16.82	0.06	0.80	**13.57**	0.06	0.80	**14.71**	0.12	0.40	**14.96**	0.19	0.40	**13.60**	0.21	0.80	**14.00**	0.20
i640-233	17.60	0.07	0.30	**10.64**	0.07	0.10	**13.18**	0.11	0.10	**15.79**	0.19	0.30	**9.92**	0.21	0.10	**11.29**	0.22
i640-234	18.87	0.07	0.60	**18.04**	0.07	0.90	**16.68**	0.12	0.90	**17.46**	0.19	0.30	**14.14**	0.22	0.10	**9.73**	0.22
i640-235	15.32	0.06	0.70	**13.59**	0.07	0.80	**14.61**	0.12	0.80	**12.77**	0.19	0.40	**13.54**	0.23	0.70	**13.93**	0.22
i640-241	40.09	0.29	0.90	40.09	0.29	0.90	**39.99**	0.33	0.10	**39.98**	1.32	0.00	**11.97**	1.16	0.60	**25.89**	1.35
i640-242	40.69	0.29	0.90	40.69	0.29	0.90	**40.63**	0.34	0.10	**40.62**	1.33	0.00	**11.28**	1.16	0.40	**18.77**	1.37
i640-243	40.71	0.29	0.90	40.71	0.29	0.90	**40.64**	0.32	0.10	**40.62**	1.23	0.00	**11.10**	1.04	0.70	**23.42**	1.30
i640-244	39.96	0.31	0.90	39.96	0.31	0.90	**39.94**	0.34	0.10	**39.91**	1.32	0.00	**10.16**	1.18	0.70	**25.19**	1.35
i640-245	40.46	0.28	0.90	40.46	0.29	0.90	**40.44**	0.29	0.10	**40.39**	1.24	0.00	**10.11**	1.13	0.20	**28.31**	1.33
i640-301	8.66	0.17	0.20	**6.54**	0.18	0.30	9.74	0.24	0.30	8.87	0.28	0.10	**4.73**	0.32	0.10	**4.86**	0.33
i640-302	5.39	0.16	0.90	5.39	0.17	0.10	7.05	0.24	0.30	7.28	0.27	0.60	5.88	0.30	0.20	**5.06**	0.32
i640-303	8.57	0.17	0.30	**6.62**	0.18	0.10	**8.41**	0.26	0.50	8.73	0.29	0.10	**6.35**	0.32	0.00	**6.04**	0.32
i640-304	9.09	0.17	0.90	9.09	0.17	0.90	9.54	0.23	0.10	9.18	0.27	0.10	**6.92**	0.32	0.60	**7.61**	0.30
i640-305	10.09	0.17	0.20	**8.27**	0.18	0.60	**9.77**	0.24	0.30	**9.34**	0.28	0.00	**5.58**	0.34	0.00	**6.27**	0.34
i640-311	24.94	0.38	0.90	24.94	0.38	0.70	**23.65**	0.41	0.10	27.46	0.56	0.00	**9.91**	0.66	0.10	**10.60**	0.59
i640-312	26.23	0.39	0.80	**25.72**	0.38	0.70	**23.48**	0.41	0.90	**26.16**	0.59	0.00	**10.49**	0.65	0.10	**12.85**	0.61
i640-313	24.67	0.38	0.90	24.67	0.38	0.70	**21.90**	0.41	0.00	26.20	0.54	0.00	**8.74**	0.64	0.20	**11.49**	0.57
i640-314	29.75	0.38	0.70	**27.87**	0.38	0.80	**27.39**	0.42	0.10	**29.73**	0.54	0.00	**8.28**	0.65	0.10	**9.85**	0.60
i640-315	30.03	0.38	0.70	**29.59**	0.38	0.80	**29.19**	0.41	0.90	**29.89**	0.59	0.00	**9.74**	0.64	0.70	**12.00**	0.58
i640-321	46.53	1.20	0.10	46.53	1.23	0.30	46.53	1.26	0.10	46.53	6.47	0.10	46.53	6.09	0.10	46.53	6.16
i640-322	46.77	1.23	0.10	46.77	1.21	0.30	46.77	1.24	0.10	46.77	6.44	0.10	46.77	6.11	0.10	46.77	6.22
i640-323	46.60	1.18	0.10	46.60	1.17	0.30	46.60	1.09	0.10	46.60	5.98	0.10	46.60	5.63	0.10	46.60	5.79
i640-324	46.68	1.16	0.10	46.68	1.13	0.30	46.68	1.16	0.10	46.68	6.08	0.10	46.68	5.74	0.10	46.68	5.94
i640-325	46.74	1.20	0.10	46.74	1.17	0.40	46.74	1.22	0.10	46.74	6.17	0.10	46.74	5.91	0.10	46.74	5.98
i640-331	8.38	0.22	0.40	**7.15**	0.23	0.20	**8.18**	0.30	0.70	8.51	0.34	0.10	9.21	0.37	0.40	8.97	0.35
i640-332	12.35	0.24	0.60	**12.12**	0.23	0.10	13.65	0.30	0.10	12.39	0.34	0.10	**7.80**	0.39	0.10	**9.62**	0.39
i640-333	9.05	0.23	0.70	**8.44**	0.22	0.20	10.30	0.29	0.90	10.29	0.35	0.30	**7.81**	0.37	0.00	**8.60**	0.39
i640-334	13.52	0.21	0.40	**10.31**	0.21	0.10	**13.21**	0.28	0.80	**12.08**	0.31	0.00	**6.95**	0.35	0.10	**6.42**	0.33
i640-335	10.99	0.20	0.80	**9.16**	0.21	0.50	11.03	0.26	0.90	11.79	0.33	0.00	**8.60**	0.38	0.10	**8.84**	0.36
i640-341	44.10	0.83	0.90	44.10	0.83	0.90	**44.00**	0.87	0.10	**43.93**	1.85	0.00	**4.77**	1.51	0.70	**20.89**	1.72
i640-342	44.30	0.80	0.90	44.30	0.80	0.90	**44.27**	0.71	0.10	**44.23**	1.87	0.00	**5.09**	1.49	0.70	**20.39**	1.86
i640-343	44.11	0.83	0.90	44.11	0.83	0.90	**44.01**	0.87	0.10	**43.95**	1.91	0.00	**4.27**	1.46	0.70	**19.35**	1.77
i640-344	44.08	0.83	0.90	44.08	0.83	0.90	**44.02**	0.85	0.10	**43.95**	1.89	0.00	**4.26**	1.52	0.70	**20.81**	1.94
i640-345	44.41	0.76	0.90	44.41	0.76	0.90	**44.35**	0.74	0.10	**44.30**	1.66	0.00	**4.43**	1.36	0.80	**14.57**	1.65
average	26.35	0.24	-	**24.37** (-1.98)	0.24	-	**24.47** (-1.88)	0.28	-	**25.12** (-1.23)	1.57	-	**18.61** (-7.74)	1.49	-	**20.38** (-5.97)	1.54

Table S9. Results of the benchmark problem set B by using the SPH.

	without centrality		with centrality															
			degree			eigenvector			closeness			vertex betweenness			edge betweenness			
name	gap	time	α	gap	time	α	gap	time	α	gap	time	α	gap	time	α	gap	time	
b01	0.00	0.00	0.90	0.00	0.00	0.80	0.00	0.00	0.90	0.27	0.00	0.20	0.00	0.00	0.00	0.00	0.00	
b02	0.51	0.00	0.90	5.06	0.00	0.90	5.30	0.00	0.50	9.04	0.00	0.00	3.61	0.00	0.10	**0.00**	0.00	
b03	0.04	0.00	0.80	**0.00**	0.00	0.10	**0.00**	0.00	0.70	**0.00**	0.00	0.30	**0.00**	0.00	0.10	**0.00**	0.00	
b04	4.58	0.00	0.90	4.85	0.00	0.90	5.08	0.00	0.90	4.58	0.00	0.30	**1.42**	0.00	0.10	**0.00**	0.00	
b05	0.00	0.00	0.80	0.00	0.00	0.80	0.00	0.00	0.60	0.00	0.00	0.60	0.00	0.00	0.10	0.00	0.00	
b06	1.64	0.00	0.70	1.64	0.00	0.70	1.64	0.00	0.70	1.64	0.00	0.70	**1.54**	0.00	0.10	**0.26**	0.00	
b07	0.00	0.00	0.90	0.00	0.00	0.60	0.00	0.00	0.80	0.00	0.00	0.10	0.00	0.00	0.10	0.00	0.00	
b08	0.00	0.00	0.80	0.00	0.00	0.20	0.00	0.00	0.80	0.00	0.00	0.10	0.00	0.00	0.10	0.00	0.00	
b09	0.04	0.00	0.90	**0.03**	0.00	0.80	0.45	0.00	0.90	0.06	0.00	0.50	**0.03**	0.00	0.10	**0.00**	0.00	
b10	4.60	0.00	0.90	**0.40**	0.00	0.90	**0.30**	0.00	0.90	4.93	0.00	0.30	**0.02**	0.00	0.60	**0.30**	0.00	
b11	2.27	0.00	0.90	**2.14**	0.00	0.90	4.14	0.00	0.90	**2.00**	0.00	0.40	**0.18**	0.00	0.00	**0.00**	0.00	
b12	0.00	0.00	0.80	0.00	0.00	0.80	0.00	0.00	0.90	0.00	0.00	0.60	0.00	0.00	0.10	0.00	0.00	
b13	7.37	0.00	0.20	**2.33**	0.00	0.10	**3.54**	0.00	0.20	**2.96**	0.00	0.00	**3.64**	0.00	0.10	**6.47**	0.00	
b14	2.27	0.00	0.50	**1.23**	0.00	0.80	2.31	0.00	0.70	**0.48**	0.00	0.70	2.28	0.00	0.20	**2.03**	0.00	
b15	0.08	0.00	0.60	**0.00**	0.00	0.40	**0.00**	0.00	0.70	**0.00**	0.00	0.70	**0.00**	0.00	0.80	**0.00**	0.00	
b16	3.06	0.00	0.80	**3.02**	0.00	0.70	**2.61**	0.00	0.70	**0.68**	0.00	0.60	**2.71**	0.00	0.10	**2.77**	0.00	
b17	0.78	0.00	0.80	1.53	0.00	0.90	0.82	0.00	0.90	1.57	0.00	0.30	0.78	0.00	0.40	**0.72**	0.00	
b18	1.40	0.00	0.80	3.01	0.00	0.40	**0.56**	0.00	0.90	2.62	0.00	0.60	2.62	0.00	0.10	1.79	0.00	
average	1.59	0.00	-	**1.40**	0.00	-	**1.49**	0.00	-	1.71	0.00	-	**1.05**	0.00	-	**0.80**	0.00	
				(-0.19)			(-0.10)			(0.12)			(-0.54)			(-0.79)		

Table S10. Results of the benchmark problem set C by using the SPH.

	without centrality		with centrality															
			degree			eigenvector			closeness			vertex betweenness			edge betweenness			
name	gap	time	α	gap	time	α	gap	time	α	gap	time	α	gap	time	α	gap	time	
c01	0.73	0.00	0.70	**0.47**	0.00	0.20	**0.56**	0.01	0.90	0.75	0.04	0.10	**0.00**	0.04	0.90	0.78	0.04	
c02	0.00	0.00	0.90	1.31	0.00	0.20	0.00	0.01	0.90	0.00	0.04	0.10	0.00	0.04	0.10	0.00	0.04	
c03	1.07	0.02	0.90	1.23	0.03	0.90	1.25	0.03	0.90	1.30	0.06	0.70	1.23	0.07	0.60	1.47	0.07	
c04	1.12	0.04	0.80	**0.65**	0.04	0.70	**0.87**	0.05	0.90	**0.87**	0.08	0.30	**0.67**	0.08	0.60	**0.91**	0.09	
c05	0.13	0.10	0.90	0.13	0.11	0.80	0.18	0.11	0.90	0.19	0.14	0.70	0.18	0.15	0.20	0.18	0.15	
c06	0.87	0.00	0.60	1.02	0.00	0.20	1.02	0.01	0.80	0.87	0.04	0.50	**0.80**	0.05	0.60	**0.84**	0.05	
c07	1.33	0.00	0.90	1.78	0.01	0.90	**1.12**	0.01	0.90	**1.00**	0.05	0.50	**0.90**	0.05	0.20	**0.47**	0.06	
c08	1.84	0.04	0.90	**1.22**	0.04	0.90	**1.19**	0.05	0.90	**1.48**	0.08	0.90	**1.00**	0.09	0.10	**1.11**	0.09	
c09	1.22	0.07	0.90	1.44	0.07	0.70	1.41	0.07	0.90	1.38	0.11	0.60	1.41	0.12	0.20	1.32	0.12	
c10	0.43	0.15	0.90	0.57	0.16	0.90	0.54	0.16	0.90	0.64	0.20	0.80	0.55	0.21	0.30	0.46	0.21	
c11	1.56	0.01	0.90	3.19	0.01	0.60	3.81	0.01	0.90	5.31	0.06	0.20	3.56	0.08	0.40	1.94	0.08	
c12	3.17	0.01	0.90	11.17	0.01	0.90	7.30	0.01	0.90	9.35	0.07	0.70	**1.83**	0.08	0.30	**1.83**	0.09	
c13	2.63	0.08	0.90	3.85	0.08	0.90	3.49	0.09	0.90	4.95	0.14	0.40	**2.29**	0.16	0.80	2.74	0.16	
c14	2.43	0.12	0.90	3.91	0.13	0.90	3.80	0.13	0.90	4.09	0.18	0.50	2.65	0.20	0.90	**1.54**	0.21	
c15	0.04	0.26	0.90	**0.01**	0.28	0.90	**0.00**	0.28	0.90	**0.03**	0.33	0.80	0.17	0.36	0.60	0.17	0.38	
c16	3.64	0.01	0.90	4.18	0.02	0.90	9.09	0.02	0.90	4.18	0.16	0.50	**2.18**	0.19	0.80	8.18	0.19	
c17	6.89	0.02	0.90	9.33	0.02	0.90	8.89	0.03	0.90	8.89	0.17	0.20	9.67	0.20	0.50	9.44	0.20	
c18	7.42	0.16	0.90	8.11	0.16	0.90	7.96	0.16	0.90	8.02	0.30	0.70	8.11	0.35	0.50	8.62	0.36	
c19	6.62	0.25	0.90	11.64	0.25	0.90	10.96	0.25	0.90	7.53	0.39	0.50	**5.48**	0.43	0.10	6.78	0.46	
c20	0.37	0.50	0.90	0.75	0.51	0.90	0.75	0.52	0.90	0.75	0.65	0.20	0.37	0.70	0.50	0.37	0.76	
average	2.18	0.09	-	3.30	0.09	-	3.21	0.10	-	3.08	0.16	-	**2.15**	0.17	-	2.46	0.18	
				(1.12)			(1.03)			(0.90)			(-0.03)			(0.28)		

Table S11. Results of the benchmark problem set D by using the SPH.

	without		with centrality														
	centrality		degree			eigenvector			closeness			vertex betweenness			edge betweenness		
name	gap	time	α	gap	time	α	gap	time	α	gap	time	α	gap	time	α	gap	time
d01	1.23	0.01	0.90	1.96	0.01	0.40	1.25	0.01	0.90	5.53	0.15	0.10	**0.79**	0.18	0.80	**1.13**	0.19
d02	4.58	0.01	0.90	**3.83**	0.01	0.50	**4.02**	0.02	0.90	**3.75**	0.16	0.10	**3.44**	0.19	0.10	**2.91**	0.20
d03	1.88	0.19	0.80	**1.45**	0.20	0.50	**1.65**	0.21	0.80	1.95	0.35	0.50	**1.62**	0.38	0.60	**1.63**	0.39
d04	1.03	0.30	0.90	**0.94**	0.33	0.30	1.16	0.34	0.90	1.30	0.47	0.80	1.33	0.50	0.40	1.33	0.51
d05	0.34	0.76	0.90	**0.28**	0.86	0.10	**0.21**	0.87	0.90	0.36	1.01	0.70	**0.21**	1.05	0.60	**0.24**	1.06
d06	7.55	0.01	0.90	**7.40**	0.01	0.20	**6.81**	0.02	0.90	8.30	0.19	0.20	**6.33**	0.22	0.80	**5.07**	0.23
d07	0.23	0.02	0.70	0.23	0.02	0.20	0.23	0.02	0.90	0.35	0.19	0.20	**0.16**	0.23	0.40	0.31	0.24
d08	2.39	0.30	0.90	3.11	0.32	0.90	3.08	0.33	0.90	3.69	0.49	0.40	**1.98**	0.53	0.70	**2.26**	0.54
d09	2.47	0.48	0.90	3.11	0.51	0.90	2.76	0.51	0.90	3.54	0.68	0.80	2.75	0.71	0.50	**2.15**	0.73
d10	0.71	1.10	0.90	1.39	1.21	0.90	1.24	1.23	0.90	1.87	1.37	0.80	1.08	1.43	0.80	0.85	1.45
d11	5.66	0.02	0.90	**4.55**	0.02	0.80	7.93	0.02	0.90	**4.76**	0.26	0.30	**3.45**	0.33	0.20	**4.00**	0.34
d12	0.24	0.04	0.90	0.86	0.04	0.90	0.67	0.04	0.90	1.00	0.28	0.30	**0.10**	0.35	0.10	**0.14**	0.36
d13	2.28	0.59	0.90	3.56	0.61	0.90	3.50	0.63	0.90	5.02	0.84	0.30	2.62	0.93	0.50	**1.67**	0.97
d14	1.39	0.92	0.90	3.00	0.95	0.90	2.78	0.97	0.90	2.92	1.17	0.50	1.68	1.29	0.20	1.48	1.34
d15	1.04	2.01	0.90	1.72	2.12	0.90	1.74	2.16	0.90	1.97	2.29	0.90	1.05	2.46	0.70	**0.88**	2.55
d16	9.85	0.05	0.90	11.69	0.05	0.90	**7.69**	0.05	0.90	13.69	0.66	0.50	10.00	0.80	0.70	**2.46**	0.83
d17	4.61	0.08	0.90	5.30	0.08	0.90	**2.78**	0.09	0.90	6.43	0.71	0.70	**2.35**	0.85	0.30	**1.83**	0.88
d18	8.22	1.21	0.90	10.75	1.17	0.90	10.76	1.18	0.90	8.53	1.79	0.80	8.65	2.01	0.20	**7.52**	2.13
d19	6.45	1.85	0.90	12.26	1.77	0.90	12.26	1.79	0.90	12.26	2.38	0.30	**6.12**	2.64	0.80	7.99	2.81
d20	1.09	3.81	0.90	1.49	3.73	0.90	1.49	3.75	0.90	1.49	4.33	0.90	**0.74**	4.69	0.50	**0.55**	4.98
average	3.16	0.66	-	3.94	0.67	-	3.70	0.68	-	4.44	0.94	-	**2.82**	1.04	-	**2.32**	1.08
				(0.78)			(0.54)			(1.28)			(-0.34)			(-0.84)	

Table S12. Results of the benchmark problem set E by using the SPH.

	without		with centrality														
	centrality		degree			eigenvector			closeness			vertex betweenness			edge betweenness		
name	gap	time	α	gap	time	α	gap	time	α	gap	time	α	gap	time	α	gap	time
e01	5.75	0.03	0.20	**1.80**	0.03	0.20	**5.30**	0.16	0.60	**4.41**	2.07	0.30	**5.01**	2.37	0.30	**5.19**	2.44
e02	8.41	0.08	0.70	**7.21**	0.08	0.80	8.44	0.20	0.40	**7.27**	2.21	0.40	**8.21**	2.48	0.30	**7.36**	2.61
e03	2.34	3.56	0.90	2.34	3.56	0.90	**2.09**	3.76	0.90	**2.32**	5.66	0.60	**2.14**	6.05	0.80	**2.06**	6.02
e04	1.34	6.17	0.90	1.34	6.17	0.50	**1.15**	6.24	0.90	1.55	8.20	0.50	**1.23**	8.59	0.70	**1.14**	8.79
e05	0.37	15.78	0.90	0.37	15.78	0.60	**0.35**	14.37	0.90	0.48	17.40	0.90	**0.32**	17.99	0.30	**0.33**	17.52
e06	7.45	0.07	0.90	7.45	0.07	0.70	7.67	0.22	0.90	8.11	2.64	0.80	7.56	3.00	0.30	**4.66**	3.11
e07	10.81	0.12	0.90	10.81	0.12	0.60	**7.99**	0.26	0.90	10.86	2.65	0.20	**7.96**	3.02	0.70	**7.50**	3.13
e08	2.85	5.79	0.90	2.85	5.79	0.70	**2.38**	6.19	0.90	3.20	8.07	0.70	**2.40**	8.95	0.80	**2.06**	9.12
e09	2.68	9.05	0.90	2.68	9.05	0.70	**2.42**	9.82	0.90	3.15	11.65	0.70	**2.26**	12.38	0.10	**2.16**	12.98
e10	0.70	22.07	0.90	0.70	22.08	0.70	**0.64**	22.65	0.90	1.09	24.66	0.80	**0.53**	25.82	0.50	**0.51**	23.41
e11	10.76	0.15	0.90	10.76	0.15	0.90	**10.18**	0.22	0.90	11.65	3.74	0.20	**6.53**	4.37	0.00	**3.53**	4.54
e12	2.99	0.28	0.90	2.99	0.29	0.90	3.34	0.36	0.90	3.49	3.84	0.60	**2.69**	4.45	0.10	**2.66**	4.65
e13	4.01	11.02	0.90	4.01	11.03	0.90	**3.89**	11.28	0.90	5.73	14.27	0.50	**3.36**	16.40	0.30	**2.40**	17.48
e14	2.33	17.70	0.90	2.33	17.71	0.90	2.55	18.25	0.90	3.43	20.27	0.50	**1.62**	23.04	0.20	**1.03**	24.28
e15	1.30	38.51	0.90	1.30	38.51	0.90	1.33	39.64	0.90	1.47	40.34	0.70	**0.64**	46.62	0.20	**0.47**	48.90
e16	12.40	0.30	0.90	12.40	0.30	0.90	**12.27**	0.37	0.90	12.40	10.20	0.90	**7.33**	11.29	0.80	**5.33**	11.78
e17	7.20	0.55	0.90	7.20	0.55	0.90	8.88	0.61	0.90	**5.44**	10.64	0.10	**4.80**	11.51	0.10	**4.56**	11.98
e18	13.30	20.19	0.90	13.30	20.19	0.90	14.36	20.03	0.90	**12.96**	30.34	0.90	**10.42**	35.34	0.40	**11.13**	38.56
e19	7.39	26.05	0.90	7.39	26.05	0.90	7.91	26.35	0.90	7.39	35.53	0.90	**5.27**	39.37	0.10	**4.46**	42.40
e20	1.71	66.65	0.90	1.71	66.65	0.90	1.71	67.92	0.90	1.71	77.15	0.70	**0.82**	91.58	0.80	**0.89**	96.30
average	5.30	12.21	-	**5.05**	12.21	-	**5.24**	12.45	-	5.41	16.58	-	**4.06**	18.73	-	**3.47**	19.50
				(-0.25)			(-0.06)			(0.11)			(-1.24)			(-1.83)	

Table S13. Results of the benchmark problem set I080 by using the SPH. Texts with a gray background are complete graph instances.

	without centrality		with centrality															
			degree			eigenvector			closeness			vertex betweenness			edge betweenness		
name	gap	time	α	gap	time	α	gap	time	α	gap	time	α	gap	time	α	gap	time
i080-001	4.58	0.00	0.60	**3.63**	0.00	0.20	**4.24**	0.00	0.60	**2.69**	0.00	0.60	**4.51**	0.00	0.60	**4.50**	0.00
i080-002	6.84	0.00	0.00	**3.05**	0.00	0.90	**6.28**	0.00	0.90	6.93	0.00	0.00	**0.00**	0.00	0.30	**4.92**	0.00
i080-003	1.10	0.00	0.20	**0.55**	0.00	0.00	**0.00**	0.00	0.80	**0.33**	0.00	0.00	**0.00**	0.00	0.00	**0.00**	0.00
i080-004	3.84	0.00	0.50	6.69	0.00	0.90	4.79	0.00	0.70	7.74	0.00	0.60	4.57	0.00	0.70	4.35	0.00
i080-005	4.45	0.00	0.80	**0.94**	0.00	0.50	**0.80**	0.00	0.70	**4.19**	0.00	0.50	**2.04**	0.00	0.70	**4.27**	0.00
i080-011	6.84	0.00	0.90	7.01	0.00	0.90	7.05	0.00	0.10	**2.50**	0.00	0.20	**4.04**	0.00	0.70	**3.62**	0.00
i080-012	2.17	0.00	0.80	**1.46**	0.00	0.70	**0.67**	0.00	0.50	**1.30**	0.00	0.20	**0.63**	0.00	0.80	**1.75**	0.00
i080-013	7.51	0.00	0.70	**0.61**	0.00	0.80	**0.13**	0.00	0.10	**0.57**	0.00	0.00	**0.00**	0.00	0.70	**1.31**	0.00
i080-014	12.49	0.00	0.90	**11.04**	0.00	0.90	**10.90**	0.00	0.90	13.70	0.00	0.50	**8.51**	0.00	0.20	**6.88**	0.00
i080-015	6.59	0.00	0.00	7.16	0.00	0.00	7.16	0.00	0.00	7.16	0.00	0.50	**3.33**	0.00	0.60	**5.84**	0.00
i080-021	25.19	0.00	0.10	25.19	0.00	0.20	25.19	0.00	0.80	25.19	0.01	0.10	25.19	0.01	0.10	25.19	0.01
i080-022	25.38	0.00	0.10	25.38	0.00	0.20	25.38	0.00	0.60	25.38	0.01	0.10	25.38	0.01	0.10	25.38	0.01
i080-023	25.30	0.00	0.10	25.30	0.00	0.30	25.30	0.00	0.40	25.30	0.01	0.10	25.30	0.01	0.10	25.30	0.01
i080-024	26.87	0.00	0.10	26.87	0.00	0.30	26.87	0.00	0.50	26.87	0.01	0.10	26.87	0.01	0.10	26.87	0.01
i080-025	27.62	0.00	0.10	27.62	0.00	0.20	27.62	0.00	0.60	27.62	0.01	0.10	27.62	0.01	0.10	27.62	0.01
i080-031	4.66	0.00	0.80	4.95	0.00	0.70	**2.47**	0.00	0.80	**1.95**	0.00	0.80	**0.65**	0.00	0.90	**2.89**	0.00
i080-032	7.16	0.00	0.50	**2.55**	0.00	0.00	**6.52**	0.00	0.50	**2.28**	0.00	0.80	**3.09**	0.00	0.20	**1.21**	0.00
i080-033	5.03	0.00	0.90	6.88	0.00	0.90	**1.78**	0.00	0.70	6.24	0.00	0.70	**3.39**	0.00	0.90	**1.31**	0.00
i080-034	4.55	0.00	0.60	6.24	0.00	0.90	**4.49**	0.00	0.90	6.52	0.00	0.10	5.39	0.00	0.20	5.19	0.00
i080-035	4.63	0.00	0.30	**1.53**	0.00	0.90	4.83	0.00	0.30	5.19	0.00	0.70	**0.66**	0.00	0.50	**1.59**	0.00
i080-041	15.46	0.00	0.10	**1.57**	0.00	0.20	**1.57**	0.00	0.00	**1.57**	0.00	0.60	**12.22**	0.00	0.70	**9.93**	0.00
i080-042	12.26	0.00	0.90	13.82	0.00	0.50	**7.69**	0.00	0.90	13.90	0.00	0.70	**7.01**	0.00	0.70	**8.54**	0.00
i080-043	8.74	0.00	0.90	14.78	0.00	0.50	**1.75**	0.00	0.90	**5.19**	0.00	0.90	**7.75**	0.00	0.30	**6.91**	0.00
i080-044	12.87	0.00	0.60	**1.34**	0.00	0.70	**1.34**	0.00	0.10	**1.34**	0.00	0.00	**1.58**	0.00	0.50	**6.58**	0.00
i080-045	12.11	0.00	0.80	**4.55**	0.00	0.70	**5.18**	0.00	0.00	**4.77**	0.00	0.40	**4.85**	0.00	0.80	**6.10**	0.00
i080-101	5.76	0.00	0.20	**4.87**	0.00	0.30	**3.14**	0.00	0.70	**1.69**	0.00	0.60	**0.00**	0.00	0.70	**0.00**	0.00
i080-102	0.06	0.00	0.60	0.87	0.00	0.90	0.06	0.00	0.90	0.81	0.00	0.90	0.46	0.00	0.80	0.88	0.00
i080-103	4.24	0.00	0.60	**4.03**	0.00	0.60	**3.62**	0.00	0.30	**3.00**	0.00	0.70	**3.45**	0.00	0.00	**2.96**	0.00
i080-104	11.42	0.00	0.70	**2.39**	0.00	0.10	**2.88**	0.00	0.80	**2.45**	0.00	0.80	**2.77**	0.00	0.20	**6.34**	0.00
i080-105	0.00	0.00	0.00	0.00	0.00	0.00	0.00	0.00	0.00	0.00	0.00	0.00	0.00	0.00	0.40	0.00	0.00
i080-111	2.04	0.00	0.50	3.46	0.00	0.60	**1.41**	0.00	0.90	**1.64**	0.00	0.20	**0.65**	0.00	0.30	**1.09**	0.00
i080-112	7.68	0.00	0.70	**1.37**	0.00	0.70	**2.48**	0.00	0.50	**5.32**	0.00	0.70	**1.12**	0.00	0.80	**5.47**	0.00
i080-113	4.82	0.00	0.00	11.15	0.00	0.90	7.54	0.00	0.00	11.15	0.00	0.80	**2.26**	0.00	0.90	**2.44**	0.00
i080-114	18.24	0.00	0.00	**16.08**	0.00	0.50	19.22	0.00	0.00	**11.80**	0.00	0.20	**4.51**	0.00	0.30	**5.31**	0.00
i080-115	9.50	0.00	0.40	**1.00**	0.00	0.40	**1.79**	0.00	0.70	**1.42**	0.00	0.20	**3.30**	0.00	0.80	**1.21**	0.00
i080-121	31.58	0.00	0.10	31.58	0.00	0.10	31.58	0.00	0.10	31.58	0.01	0.10	31.58	0.01	0.10	31.58	0.01
i080-122	31.97	0.00	0.10	31.97	0.00	0.30	31.97	0.00	0.80	31.97	0.01	0.10	31.97	0.01	0.10	31.97	0.01
i080-123	31.36	0.00	0.10	31.36	0.00	0.40	31.36	0.00	0.10	31.36	0.01	0.10	31.36	0.01	0.10	31.36	0.01
i080-124	30.68	0.00	0.10	30.68	0.00	0.10	30.68	0.00	0.10	30.68	0.01	0.10	30.68	0.01	0.10	30.68	0.01
i080-125	31.11	0.00	0.10	31.11	0.00	0.20	31.11	0.00	0.80	31.11	0.01	0.10	31.11	0.01	0.10	31.11	0.01
i080-131	10.04	0.00	0.20	**4.86**	0.00	0.10	**3.81**	0.00	0.50	**6.59**	0.00	0.20	**6.89**	0.00	0.00	**4.90**	0.00
i080-132	7.73	0.00	0.50	**5.29**	0.00	0.50	**5.31**	0.00	0.50	**6.34**	0.00	0.60	**5.04**	0.00	0.30	**6.63**	0.00
i080-133	1.82	0.00	0.10	**0.84**	0.00	0.70	1.99	0.00	0.80	3.12	0.00	0.80	**1.36**	0.00	0.20	**0.88**	0.00
i080-134	6.66	0.00	0.50	**4.11**	0.00	0.60	**4.37**	0.00	0.90	7.37	0.00	0.40	**2.55**	0.00	0.50	**5.30**	0.00
i080-135	3.20	0.00	0.70	**0.48**	0.00	0.30	**2.59**	0.00	0.50	**0.34**	0.00	0.90	**3.01**	0.00	0.90	**3.03**	0.00
i080-141	10.94	0.00	0.80	**5.59**	0.00	0.60	**5.59**	0.00	0.40	**5.59**	0.00	0.10	**4.23**	0.00	0.50	**6.76**	0.00
i080-142	13.63	0.00	0.70	**12.70**	0.00	0.30	**7.61**	0.00	0.10	14.29	0.00	0.90	**12.13**	0.00	0.80	**11.78**	0.00
i080-143	4.51	0.00	0.10	**3.44**	0.00	0.20	**1.67**	0.00	0.30	6.81	0.00	0.60	**2.95**	0.00	0.70	7.60	0.00
i080-144	13.30	0.00	0.70	14.99	0.00	0.60	**7.16**	0.00	0.50	**7.17**	0.00	0.50	**7.71**	0.00	0.70	**6.73**	0.00
i080-145	12.77	0.00	0.10	**7.00**	0.00	0.60	**11.84**	0.00	0.00	**2.67**	0.00	0.00	**2.67**	0.00	0.20	**6.84**	0.00

Table S13. (Continued) Results of the benchmark problem set I080 by using the SPH. Texts with a gray background are complete graph instances.

	without centrality		with centrality															
			degree			eigenvector			closeness			vertex betweenness			edge betweenness		
name	gap	time	α	gap	time	α	gap	time	α	gap	time	α	gap	time	α	gap	time
i080-201	2.89	0.00	0.60	3.76	0.00	0.80	**1.17**	0.00	0.50	3.69	0.00	0.60	**0.01**	0.00	0.80	**2.36**	0.00
i080-202	1.56	0.00	0.20	2.39	0.00	0.10	**0.57**	0.00	0.70	2.42	0.00	0.50	**0.21**	0.00	0.60	**0.84**	0.00
i080-203	5.46	0.00	0.30	6.55	0.00	0.90	**5.44**	0.00	0.10	6.23	0.00	0.10	**2.72**	0.00	0.10	**2.92**	0.00
i080-204	4.21	0.00	0.80	**3.34**	0.00	0.50	**3.15**	0.00	0.10	**3.10**	0.00	0.10	**3.39**	0.00	0.60	4.26	0.00
i080-205	8.02	0.00	0.90	**2.32**	0.00	0.10	**2.39**	0.00	0.80	**2.23**	0.00	0.80	**2.09**	0.00	0.30	**2.99**	0.00
i080-211	7.06	0.00	0.90	9.15	0.00	0.80	8.27	0.00	0.80	9.11	0.00	0.10	**6.22**	0.00	0.30	**5.77**	0.00
i080-212	18.13	0.00	0.90	**12.74**	0.00	0.90	**12.40**	0.00	0.90	**12.75**	0.00	0.50	**7.38**	0.00	0.40	**5.99**	0.00
i080-213	14.02	0.00	0.20	14.71	0.00	0.20	14.46	0.00	0.90	16.69	0.00	0.30	**3.92**	0.00	0.50	**3.41**	0.00
i080-214	19.50	0.00	0.90	21.02	0.00	0.90	21.56	0.00	0.30	20.19	0.00	0.10	**2.63**	0.00	0.50	**2.99**	0.00
i080-215	20.93	0.00	0.20	**8.58**	0.00	0.20	**8.42**	0.00	0.50	**13.56**	0.00	0.40	**9.23**	0.00	0.30	**4.80**	0.00
i080-221	38.89	0.00	0.10	38.89	0.00	0.40	38.89	0.00	0.10	38.89	0.01	0.10	38.89	0.01	0.10	38.89	0.01
i080-222	38.81	0.00	0.10	38.81	0.00	0.40	38.81	0.00	0.10	38.81	0.01	0.10	38.81	0.01	0.10	38.81	0.01
i080-223	39.01	0.00	0.10	39.01	0.00	0.30	39.01	0.00	0.10	39.01	0.01	0.10	39.01	0.01	0.10	39.01	0.01
i080-224	38.46	0.00	0.10	38.46	0.00	0.50	38.46	0.00	0.10	38.46	0.01	0.10	38.46	0.01	0.10	38.46	0.01
i080-225	38.44	0.00	0.10	38.44	0.00	0.50	38.44	0.00	0.10	38.44	0.01	0.10	38.44	0.01	0.10	38.44	0.01
i080-231	7.60	0.00	0.80	**3.83**	0.00	0.10	**4.80**	0.00	0.40	**4.75**	0.00	0.70	**3.67**	0.00	0.20	**3.21**	0.00
i080-232	8.99	0.00	0.80	**4.91**	0.00	0.80	**5.17**	0.00	0.50	**5.39**	0.00	0.50	**5.15**	0.00	0.90	**6.70**	0.00
i080-233	4.21	0.00	0.60	4.51	0.00	0.90	**4.04**	0.00	0.20	6.28	0.00	0.60	5.16	0.00	0.20	**3.70**	0.00
i080-234	3.36	0.00	0.80	6.41	0.00	0.90	**3.10**	0.00	0.90	6.41	0.00	0.90	3.67	0.00	0.80	4.91	0.00
i080-235	5.56	0.00	0.80	**2.99**	0.00	0.80	**3.04**	0.00	0.90	**3.34**	0.00	0.90	**3.24**	0.00	0.90	**4.14**	0.00
i080-241	26.14	0.00	0.90	26.31	0.00	0.90	26.14	0.00	0.90	26.14	0.00	0.60	**3.67**	0.00	0.70	**4.76**	0.00
i080-242	17.26	0.00	0.50	**16.08**	0.00	0.20	**16.08**	0.00	0.80	17.26	0.00	0.60	**4.05**	0.00	0.50	**6.12**	0.00
i080-243	21.45	0.00	0.20	**14.48**	0.00	0.20	**14.48**	0.00	0.90	22.37	0.00	0.30	**5.37**	0.00	0.60	**8.49**	0.00
i080-244	9.17	0.00	0.80	15.86	0.00	0.60	15.86	0.00	0.40	15.72	0.00	0.60	**4.36**	0.00	0.60	**4.86**	0.00
i080-245	28.50	0.00	0.80	28.58	0.00	0.50	28.55	0.00	0.80	28.53	0.00	0.70	**6.32**	0.00	0.70	**8.12**	0.00
i080-301	6.95	0.00	0.90	**5.99**	0.00	0.30	**2.24**	0.00	0.70	**5.28**	0.00	0.70	**2.20**	0.00	0.80	**0.47**	0.00
i080-302	5.50	0.00	0.80	**4.24**	0.00	0.10	**5.25**	0.00	0.90	5.67	0.00	0.30	**2.57**	0.00	0.70	**2.54**	0.00
i080-303	2.28	0.00	0.60	**1.26**	0.00	0.10	2.74	0.00	0.70	**1.70**	0.00	0.00	**1.11**	0.00	0.50	**1.72**	0.00
i080-304	1.38	0.00	0.80	3.72	0.00	0.70	4.00	0.00	0.90	3.86	0.00	0.90	**0.28**	0.00	0.50	**0.20**	0.00
i080-305	4.47	0.00	0.90	**3.13**	0.00	0.70	**3.03**	0.00	0.20	5.01	0.00	0.10	**1.53**	0.00	0.10	**1.31**	0.00
i080-311	17.93	0.00	0.90	20.02	0.00	0.90	19.24	0.00	0.90	19.03	0.00	0.10	**6.43**	0.00	0.50	**5.05**	0.00
i080-312	18.93	0.00	0.90	20.75	0.00	0.40	**11.54**	0.00	0.90	20.69	0.00	0.40	**6.57**	0.00	0.50	**6.42**	0.00
i080-313	10.18	0.00	0.90	12.80	0.00	0.90	11.72	0.00	0.30	15.72	0.00	0.70	**4.94**	0.00	0.60	**5.58**	0.00
i080-314	6.54	0.00	0.60	**4.32**	0.00	0.90	**5.00**	0.00	0.70	9.72	0.00	0.60	**3.71**	0.00	0.70	**4.24**	0.00
i080-315	16.50	0.00	0.10	**12.20**	0.00	0.70	**11.91**	0.00	0.50	**12.19**	0.00	0.10	**3.42**	0.00	0.40	**2.73**	0.00
i080-321	40.56	0.00	0.10	40.56	0.00	0.40	40.56	0.00	0.10	40.56	0.01	0.10	40.56	0.01	0.10	40.56	0.01
i080-322	39.85	0.00	0.10	39.85	0.00	0.30	39.85	0.00	0.50	39.85	0.01	0.10	39.85	0.01	0.10	39.85	0.01
i080-323	40.02	0.00	0.10	40.02	0.00	0.50	40.02	0.00	0.80	40.02	0.01	0.10	40.02	0.01	0.10	40.02	0.01
i080-324	40.31	0.00	0.10	40.31	0.00	0.30	40.31	0.00	0.10	40.31	0.01	0.10	40.31	0.01	0.10	40.31	0.01
i080-325	41.03	0.00	0.10	41.03	0.00	0.30	41.03	0.00	0.80	41.03	0.01	0.10	41.03	0.01	0.10	41.03	0.01
i080-331	11.12	0.00	0.80	**7.36**	0.00	0.90	**9.11**	0.00	0.90	**8.50**	0.00	0.50	**4.24**	0.00	0.60	**4.49**	0.00
i080-332	2.68	0.00	0.20	**0.56**	0.00	0.70	**2.10**	0.00	0.60	3.47	0.00	0.60	2.93	0.00	0.50	**1.34**	0.00
i080-333	7.12	0.00	0.50	**4.08**	0.00	0.90	**6.55**	0.00	0.30	**3.67**	0.00	0.50	**2.91**	0.00	0.20	**3.39**	0.00
i080-334	3.90	0.00	0.80	3.98	0.00	0.90	4.44	0.00	0.90	7.12	0.00	0.40	**3.07**	0.00	0.90	**2.93**	0.00
i080-335	6.21	0.00	0.90	**4.85**	0.00	0.70	6.35	0.00	0.60	6.39	0.00	0.30	**0.85**	0.00	0.10	**1.24**	0.00
i080-341	18.29	0.00	0.90	19.10	0.00	0.90	**15.89**	0.00	0.90	19.03	0.00	0.60	**3.49**	0.00	0.30	**3.81**	0.00
i080-342	29.49	0.00	0.90	29.74	0.00	0.90	29.54	0.00	0.90	29.54	0.00	0.40	**3.63**	0.00	0.40	**4.52**	0.00
i080-343	32.85	0.00	0.90	33.33	0.00	0.90	32.95	0.00	0.90	32.88	0.00	0.10	**6.07**	0.00	0.60	**5.08**	0.00
i080-344	31.83	0.00	0.70	32.02	0.00	0.90	31.95	0.00	0.90	31.83	0.00	0.10	**3.55**	0.00	0.30	**3.75**	0.00
i080-345	15.25	0.00	0.90	24.83	0.00	0.90	15.53	0.00	0.90	15.55	0.00	0.70	**3.96**	0.00	0.70	**4.38**	0.00
average	14.58	0.00	-	**13.44**	0.00	-	**12.86**	0.00	-	**13.61**	0.00	-	**9.74**	0.00	-	**10.13**	0.00
				(-1.14)			(-1.72)			(-0.97)			(-4.84)			(-4.45)	

Table S14. Results of the benchmark problem set I160 by using the SPH. Texts with a gray background are complete graph instances.

	without centrality		with centrality															
			degree			eigenvector			closeness			vertex betweenness			edge betweenness		
name	gap	time	α	gap	time	α	gap	time	α	gap	time	α	gap	time	α	gap	time
i160-001	3.11	0.00	0.40	3.57	0.00	0.90	3.92	0.00	0.60	**1.40**	0.00	0.50	3.26	0.00	0.30	**1.65**	0.00
i160-002	4.08	0.00	0.60	**2.31**	0.00	0.50	**0.09**	0.00	0.90	**2.75**	0.00	0.90	**1.09**	0.00	0.80	**0.54**	0.00
i160-003	3.50	0.00	0.90	3.66	0.00	0.80	**3.26**	0.00	0.90	3.64	0.00	0.10	**2.05**	0.00	0.70	**3.17**	0.00
i160-004	5.02	0.00	0.30	**4.97**	0.00	0.10	**4.71**	0.00	0.10	**4.42**	0.00	0.80	5.03	0.00	0.90	5.02	0.00
i160-005	7.45	0.00	0.90	**4.88**	0.00	0.70	**7.13**	0.00	0.30	7.78	0.00	0.50	**1.48**	0.00	0.20	**1.89**	0.00
i160-011	7.32	0.00	0.90	7.88	0.00	0.80	7.39	0.00	0.10	7.55	0.01	0.40	**5.14**	0.01	0.30	**5.13**	0.01
i160-012	6.06	0.00	0.40	6.38	0.00	0.90	**5.24**	0.00	0.50	6.79	0.01	0.90	**6.00**	0.01	0.30	**5.60**	0.01
i160-013	8.28	0.00	0.90	8.43	0.00	0.80	**5.74**	0.00	0.80	**8.04**	0.01	0.10	**6.94**	0.01	0.80	**7.88**	0.01
i160-014	4.98	0.00	0.40	5.19	0.00	0.80	5.49	0.00	0.10	5.41	0.01	0.90	5.46	0.01	0.90	5.72	0.01
i160-015	8.07	0.00	0.90	**6.67**	0.00	0.60	**7.02**	0.00	0.70	**6.76**	0.01	0.30	**6.02**	0.01	0.80	**5.71**	0.01
i160-021	30.47	0.01	0.10	30.47	0.01	0.10	30.47	0.01	0.10	30.47	0.04	0.10	30.47	0.04	0.10	30.47	0.04
i160-022	29.74	0.01	0.10	29.74	0.01	0.20	29.74	0.01	0.10	29.74	0.04	0.10	29.74	0.04	0.10	29.74	0.04
i160-023	29.90	0.01	0.10	29.90	0.01	0.10	29.90	0.01	0.10	29.90	0.04	0.10	29.90	0.04	0.10	29.90	0.04
i160-024	28.96	0.01	0.10	28.96	0.01	0.20	28.96	0.01	0.10	28.96	0.04	0.10	28.96	0.04	0.10	28.96	0.04
i160-025	28.99	0.01	0.10	28.99	0.01	0.40	28.99	0.01	0.10	28.99	0.04	0.10	28.99	0.04	0.10	28.99	0.04
i160-031	2.51	0.00	0.70	3.31	0.00	0.90	**2.27**	0.00	0.90	**2.50**	0.00	0.90	2.63	0.01	0.80	**2.00**	0.01
i160-032	5.72	0.00	0.20	**3.83**	0.00	0.70	**4.72**	0.00	0.00	**4.10**	0.00	0.10	**4.29**	0.00	0.10	**3.34**	0.01
i160-033	3.85	0.00	0.80	4.79	0.00	0.90	4.43	0.00	0.60	5.53	0.00	0.50	4.57	0.01	0.90	4.89	0.01
i160-034	9.75	0.00	0.60	**0.63**	0.00	0.50	**2.90**	0.00	0.50	**0.85**	0.00	0.60	**3.40**	0.01	0.80	**1.72**	0.01
i160-035	2.85	0.00	0.90	6.24	0.00	0.90	3.26	0.00	0.40	6.23	0.00	0.60	**2.73**	0.01	0.90	**2.69**	0.01
i160-041	8.50	0.00	0.90	**8.05**	0.00	0.40	**1.16**	0.00	0.80	**8.27**	0.01	0.40	**7.54**	0.01	0.90	**4.72**	0.01
i160-042	18.26	0.00	0.40	**7.58**	0.00	0.50	**7.83**	0.00	0.00	**7.84**	0.01	0.40	**8.12**	0.01	0.30	**11.37**	0.01
i160-043	14.91	0.00	0.90	14.91	0.00	0.70	14.91	0.00	0.10	14.91	0.01	0.60	**3.53**	0.01	0.70	**5.32**	0.01
i160-044	9.37	0.00	0.90	22.76	0.00	0.90	**6.41**	0.00	0.90	12.14	0.01	0.40	**8.31**	0.01	0.80	**8.72**	0.01
i160-045	4.79	0.00	0.90	6.94	0.00	0.70	7.14	0.00	0.60	9.94	0.01	0.90	**4.50**	0.01	0.70	6.80	0.01
i160-101	1.96	0.00	0.90	**0.97**	0.00	0.40	**1.92**	0.00	0.50	**0.47**	0.00	0.10	**1.03**	0.00	0.10	**0.36**	0.01
i160-102	6.45	0.00	0.90	**3.47**	0.00	0.40	**5.25**	0.00	0.90	**5.82**	0.00	0.10	**2.54**	0.00	0.00	**2.72**	0.01
i160-103	0.72	0.00	0.90	**0.41**	0.00	0.80	**0.54**	0.00	0.80	**0.66**	0.00	0.70	0.84	0.00	0.90	**0.63**	0.01
i160-104	5.36	0.00	0.70	**0.13**	0.00	0.30	**0.28**	0.00	0.40	**0.07**	0.00	0.40	**0.17**	0.00	0.70	**1.55**	0.01
i160-105	10.19	0.00	0.90	**2.57**	0.00	0.70	**5.03**	0.00	0.50	**9.30**	0.00	0.50	**0.50**	0.00	0.50	**1.75**	0.01
i160-111	15.36	0.00	0.30	**10.29**	0.00	0.90	**9.53**	0.00	0.70	**14.07**	0.01	0.60	**10.15**	0.01	0.80	**9.17**	0.01
i160-112	5.36	0.00	0.50	5.47	0.00	0.40	**4.41**	0.00	0.60	**5.29**	0.01	0.60	6.05	0.01	0.60	**4.94**	0.01
i160-113	11.85	0.00	0.90	**5.96**	0.00	0.80	**6.89**	0.00	0.90	**5.02**	0.01	0.10	**5.38**	0.01	0.50	**6.82**	0.01
i160-114	5.57	0.00	0.50	6.58	0.00	0.50	6.34	0.00	0.10	**4.95**	0.01	0.60	**3.75**	0.01	0.30	**3.75**	0.01
i160-115	4.45	0.00	0.40	5.71	0.00	0.60	4.57	0.00	0.50	**4.14**	0.01	0.10	**3.06**	0.01	0.30	**4.27**	0.01
i160-121	35.84	0.01	0.10	35.84	0.01	0.40	35.84	0.01	0.90	35.84	0.04	0.10	35.84	0.04	0.10	35.84	0.04
i160-122	36.46	0.01	0.10	36.46	0.01	0.30	36.46	0.01	0.90	36.46	0.04	0.10	36.46	0.04	0.10	36.46	0.04
i160-123	36.31	0.01	0.10	36.31	0.01	0.30	36.31	0.01	0.80	36.31	0.04	0.10	36.31	0.04	0.10	36.31	0.04
i160-124	37.76	0.01	0.10	37.76	0.01	0.20	37.76	0.01	0.90	37.76	0.04	0.10	37.76	0.04	0.10	37.76	0.04
i160-125	36.88	0.01	0.10	36.88	0.01	0.30	36.88	0.01	0.80	36.88	0.04	0.10	36.88	0.04	0.10	36.88	0.04
i160-131	2.04	0.00	0.80	2.46	0.00	0.80	**0.44**	0.00	0.90	**0.70**	0.00	0.60	**0.49**	0.01	0.90	**1.01**	0.01
i160-132	4.02	0.00	0.90	**3.97**	0.00	0.80	4.08	0.00	0.90	6.75	0.00	0.90	**3.79**	0.01	0.90	**3.58**	0.01
i160-133	10.32	0.00	0.00	**10.22**	0.00	0.90	10.64	0.00	0.10	11.39	0.00	0.20	**5.48**	0.01	0.30	**6.79**	0.01
i160-134	6.91	0.00	0.00	**1.08**	0.00	0.80	**4.76**	0.00	0.00	**1.20**	0.00	0.80	**2.92**	0.01	0.80	**4.37**	0.01
i160-135	6.22	0.00	0.90	**5.14**	0.00	0.80	**4.75**	0.00	0.00	**3.14**	0.00	0.30	**2.98**	0.01	0.30	**1.87**	0.01
i160-141	28.09	0.00	0.80	28.25	0.00	0.80	28.09	0.00	0.70	28.09	0.01	0.50	**7.67**	0.01	0.50	**7.85**	0.01
i160-142	27.83	0.00	0.90	27.95	0.00	0.90	27.83	0.00	0.10	27.83	0.01	0.40	**5.47**	0.01	0.70	**6.27**	0.01
i160-143	9.65	0.00	0.70	15.49	0.00	0.50	12.84	0.00	0.20	11.88	0.01	0.10	**8.15**	0.01	0.90	**8.34**	0.01
i160-144	7.15	0.00	0.90	8.95	0.00	0.90	9.33	0.00	0.00	9.23	0.01	0.60	**6.19**	0.01	0.90	**6.15**	0.01
i160-145	12.90	0.00	0.10	13.23	0.00	0.50	**11.50**	0.00	0.00	**12.55**	0.01	0.70	**7.82**	0.01	0.50	**9.82**	0.01

Table S14. (Continued) Results of the benchmark problem set I160 by using the SPH. Texts with a gray background are complete graph instances.

	without centrality		with centrality															
			degree			eigenvector			closeness			vertex betweenness			edge betweenness		
name	gap	time	α	gap	time	α	gap	time	α	gap	time	α	gap	time	α	gap	time
i160-201	4.28	0.00	0.30	6.17	0.00	0.40	**1.43**	0.00	0.80	6.22	0.01	0.50	**4.08**	0.01	0.10	**2.50**	0.01
i160-202	4.81	0.00	0.20	8.73	0.00	0.90	**3.54**	0.00	0.10	7.17	0.01	0.10	5.50	0.01	0.90	**3.78**	0.01
i160-203	2.46	0.00	0.90	4.20	0.00	0.80	3.75	0.00	0.90	5.02	0.01	0.70	**1.86**	0.01	0.80	**2.42**	0.01
i160-204	9.98	0.00	0.50	**9.32**	0.00	0.80	10.03	0.00	0.70	**9.73**	0.00	0.00	**5.38**	0.01	0.20	**4.11**	0.01
i160-205	4.77	0.00	0.90	**3.65**	0.00	0.10	**4.35**	0.00	0.90	6.77	0.01	0.60	**1.82**	0.01	0.30	**1.73**	0.01
i160-211	8.65	0.00	0.70	15.55	0.00	0.90	9.00	0.00	0.80	8.95	0.01	0.40	**5.80**	0.01	0.20	**5.59**	0.01
i160-212	10.34	0.00	0.90	10.36	0.00	0.80	**10.29**	0.00	0.90	11.26	0.01	0.10	**7.42**	0.01	0.50	**4.97**	0.01
i160-213	18.75	0.00	0.60	**16.24**	0.00	0.70	**12.46**	0.00	0.90	**17.27**	0.01	0.20	**6.20**	0.01	0.50	**6.47**	0.01
i160-214	10.36	0.00	0.40	**6.02**	0.00	0.70	**6.92**	0.00	0.10	**9.28**	0.01	0.10	**7.03**	0.01	0.20	**6.75**	0.01
i160-215	22.40	0.00	0.90	22.64	0.00	0.90	22.62	0.00	0.90	22.78	0.01	0.20	**8.08**	0.01	0.20	**9.09**	0.01
i160-221	41.15	0.01	0.10	41.15	0.01	0.40	41.15	0.01	0.90	41.15	0.04	0.10	41.15	0.04	0.10	41.15	0.05
i160-222	42.09	0.01	0.10	42.09	0.01	0.30	42.09	0.01	0.80	42.09	0.04	0.10	42.09	0.04	0.10	42.09	0.05
i160-223	41.35	0.01	0.10	41.35	0.01	0.40	41.35	0.01	0.10	41.35	0.04	0.10	41.35	0.04	0.10	41.35	0.05
i160-224	41.96	0.01	0.10	41.96	0.01	0.20	41.96	0.01	0.10	41.96	0.04	0.10	41.96	0.04	0.10	41.96	0.05
i160-225	40.67	0.01	0.10	40.67	0.01	0.30	40.67	0.01	0.90	40.67	0.04	0.10	40.67	0.04	0.10	40.67	0.05
i160-231	10.28	0.00	0.40	**10.22**	0.00	0.80	**10.10**	0.00	0.80	**7.16**	0.01	0.10	**6.05**	0.01	0.30	**6.54**	0.01
i160-232	10.12	0.00	0.70	**7.36**	0.00	0.20	**8.41**	0.00	0.20	**6.48**	0.01	0.20	**3.50**	0.01	0.20	**2.25**	0.01
i160-233	7.73	0.00	0.40	**6.79**	0.00	0.40	**5.39**	0.00	0.70	**6.36**	0.01	0.50	**5.33**	0.01	0.40	**4.63**	0.01
i160-234	3.13	0.00	0.90	**0.62**	0.00	0.90	**2.58**	0.00	0.80	**1.05**	0.01	0.40	**2.21**	0.01	0.50	**2.19**	0.01
i160-235	10.82	0.00	0.70	**8.56**	0.00	0.70	**7.93**	0.00	0.20	**8.97**	0.01	0.10	**5.40**	0.01	0.10	**4.23**	0.01
i160-241	34.17	0.00	0.90	34.29	0.01	0.90	34.25	0.01	0.10	34.17	0.01	0.70	**4.15**	0.02	0.70	**6.66**	0.02
i160-242	33.45	0.00	0.90	33.51	0.01	0.90	33.49	0.01	0.90	33.45	0.01	0.30	**4.45**	0.02	0.70	**5.13**	0.02
i160-243	34.91	0.00	0.90	34.97	0.01	0.90	34.95	0.01	0.90	34.91	0.01	0.40	**5.39**	0.02	0.70	**7.36**	0.02
i160-244	33.59	0.00	0.90	33.87	0.01	0.90	33.59	0.01	0.90	33.59	0.01	0.20	**4.13**	0.01	0.70	**6.48**	0.02
i160-245	33.93	0.00	0.90	34.15	0.01	0.90	34.05	0.01	0.90	33.93	0.01	0.50	**5.17**	0.02	0.70	**6.88**	0.02
i160-301	4.66	0.00	0.80	**4.60**	0.00	0.60	**4.03**	0.00	0.50	6.82	0.01	0.20	**3.32**	0.01	0.70	**1.15**	0.01
i160-302	3.12	0.00	0.70	4.12	0.00	0.90	3.81	0.00	0.90	4.91	0.01	0.90	**2.96**	0.01	0.80	**2.93**	0.01
i160-303	5.58	0.00	0.30	**1.86**	0.00	0.50	**3.38**	0.00	0.90	**2.63**	0.01	0.10	**0.29**	0.01	0.20	**1.72**	0.01
i160-304	4.20	0.00	0.50	**3.44**	0.00	0.20	**2.92**	0.00	0.80	4.97	0.01	0.40	**1.98**	0.01	0.50	**3.56**	0.01
i160-305	4.22	0.00	0.90	**3.16**	0.00	0.30	**2.76**	0.00	0.50	**3.31**	0.01	0.40	**1.87**	0.01	0.50	**1.82**	0.01
i160-311	15.96	0.00	0.80	19.89	0.00	0.90	20.55	0.01	0.60	18.85	0.01	0.40	**5.93**	0.01	0.30	**6.04**	0.01
i160-312	26.02	0.00	0.20	**22.09**	0.00	0.20	**22.12**	0.01	0.90	26.43	0.01	0.40	**5.88**	0.01	0.40	**5.29**	0.01
i160-313	24.38	0.00	0.80	**21.17**	0.00	0.40	**20.62**	0.01	0.90	**20.73**	0.01	0.20	**5.55**	0.01	0.40	**5.84**	0.01
i160-314	25.46	0.00	0.60	**21.40**	0.00	0.80	**21.03**	0.01	0.90	25.99	0.01	0.20	**6.89**	0.01	0.60	**6.61**	0.01
i160-315	26.06	0.00	0.60	**17.72**	0.00	0.90	**21.07**	0.01	0.10	**20.39**	0.01	0.30	**4.03**	0.01	0.20	**3.46**	0.01
i160-321	43.27	0.01	0.10	43.27	0.02	0.40	43.27	0.02	0.10	43.27	0.05	0.10	43.27	0.05	0.10	43.27	0.05
i160-322	43.70	0.01	0.10	43.70	0.02	0.30	43.70	0.02	0.10	43.70	0.05	0.10	43.70	0.05	0.10	43.70	0.05
i160-323	43.38	0.01	0.10	43.38	0.02	0.40	43.38	0.02	0.10	43.38	0.05	0.10	43.38	0.05	0.10	43.38	0.05
i160-324	43.42	0.01	0.10	43.42	0.02	0.50	43.42	0.02	0.10	43.42	0.05	0.10	43.42	0.05	0.10	43.42	0.05
i160-325	43.59	0.01	0.10	43.59	0.02	0.40	43.59	0.02	0.10	43.59	0.05	0.10	43.59	0.05	0.10	43.59	0.05
i160-331	7.06	0.00	0.90	**6.90**	0.00	0.10	**6.52**	0.00	0.90	7.29	0.01	0.40	**4.83**	0.01	0.90	**4.93**	0.01
i160-332	6.86	0.00	0.30	7.93	0.00	0.90	7.07	0.00	0.30	9.73	0.01	0.10	**3.65**	0.01	0.40	**2.31**	0.01
i160-333	4.66	0.00	0.30	4.66	0.00	0.90	4.97	0.00	0.10	5.16	0.01	0.10	**3.25**	0.01	0.10	**2.87**	0.01
i160-334	5.29	0.00	0.90	5.94	0.00	0.90	**5.25**	0.00	0.80	6.11	0.01	0.50	**4.88**	0.01	0.90	5.74	0.01
i160-335	6.05	0.00	0.20	**2.84**	0.00	0.90	**5.54**	0.00	0.10	6.23	0.01	0.30	**3.73**	0.01	0.60	**3.62**	0.01
i160-341	37.13	0.01	0.90	37.28	0.01	0.90	37.17	0.01	0.90	37.13	0.02	0.60	**4.90**	0.02	0.70	**5.78**	0.02
i160-342	37.28	0.01	0.90	37.49	0.01	0.90	37.43	0.01	0.90	37.28	0.02	0.30	**3.14**	0.02	0.70	**3.77**	0.02
i160-343	38.82	0.01	0.90	38.92	0.01	0.90	38.89	0.01	0.90	38.83	0.02	0.30	**3.09**	0.02	0.60	**3.92**	0.02
i160-344	38.00	0.01	0.90	38.24	0.01	0.90	38.03	0.01	0.80	38.00	0.02	0.60	**3.65**	0.02	0.60	**4.55**	0.02
i160-345	37.38	0.01	0.90	37.48	0.01	0.90	37.43	0.01	0.80	37.38	0.02	0.50	**3.70**	0.02	0.70	**4.08**	0.02
average	17.44	0.00	-	**16.92** (-0.52)	0.00	-	**16.50** (-0.94)	0.01	-	**17.08** (-0.36)	0.02	-	**11.09** (-6.35)	0.02	-	**11.22** (-6.22)	0.02

Table S15. Results of the benchmark problem set I320 by using the SPH. Texts with a gray background are complete graph instances.

	without centrality		with centrality														
			degree			eigenvector			closeness			vertex betweenness			edge betweenness		
name	gap	time	α	gap	time	α	gap	time	α	gap	time	α	gap	time	α	gap	time
i320-001	0.08	0.00	0.50	2.10	0.00	0.30	**0.07**	0.00	0.90	0.89	0.02	0.90	0.23	0.02	0.70	2.11	0.02
i320-002	1.83	0.00	0.60	2.06	0.00	0.50	2.05	0.00	0.50	2.72	0.02	0.20	**1.27**	0.02	0.90	1.96	0.02
i320-003	9.75	0.00	0.80	**8.97**	0.00	0.20	**9.29**	0.00	0.80	**8.86**	0.02	0.50	**7.18**	0.02	0.40	**3.94**	0.02
i320-004	4.83	0.00	0.10	**4.11**	0.00	0.90	5.01	0.00	0.50	**3.95**	0.02	0.20	**3.60**	0.02	0.40	**4.48**	0.02
i320-005	4.50	0.00	0.70	**4.30**	0.00	0.20	**3.89**	0.00	0.90	**3.55**	0.02	0.50	**3.91**	0.02	0.90	**3.43**	0.02
i320-011	5.56	0.00	0.90	6.36	0.00	0.90	**3.39**	0.01	0.80	**2.50**	0.03	0.30	**3.98**	0.03	0.20	**5.22**	0.03
i320-012	11.56	0.00	0.80	**11.10**	0.00	0.60	**11.51**	0.01	0.20	**6.67**	0.03	0.40	**5.12**	0.03	0.70	**10.27**	0.03
i320-013	5.31	0.00	0.60	5.55	0.00	0.40	5.37	0.01	0.20	**5.12**	0.03	0.90	5.33	0.03	0.90	**4.91**	0.03
i320-014	2.16	0.00	0.70	3.84	0.00	0.90	**2.08**	0.01	0.90	**1.66**	0.03	0.80	**1.92**	0.03	0.80	**1.61**	0.03
i320-015	6.96	0.00	0.90	7.32	0.00	0.80	7.70	0.01	0.90	7.33	0.03	0.60	**1.94**	0.03	0.40	**6.11**	0.03
i320-021	31.68	0.03	0.10	31.68	0.03	0.10	31.68	0.04	0.10	31.68	0.30	0.10	31.68	0.29	0.10	31.68	0.31
i320-022	31.57	0.03	0.10	31.57	0.03	0.20	31.57	0.04	0.10	31.57	0.30	0.10	31.57	0.29	0.10	31.57	0.31
i320-023	33.70	0.03	0.10	33.70	0.03	0.10	33.70	0.04	0.10	33.70	0.30	0.10	33.70	0.29	0.10	33.70	0.31
i320-024	31.81	0.03	0.10	31.81	0.03	0.40	31.81	0.04	0.80	31.81	0.30	0.10	31.81	0.29	0.10	31.81	0.32
i320-025	32.00	0.03	0.10	32.00	0.03	0.20	32.00	0.04	0.10	32.00	0.30	0.10	32.00	0.29	0.10	32.00	0.31
i320-031	3.27	0.00	0.00	**2.20**	0.00	0.10	**1.77**	0.00	0.90	**2.51**	0.02	0.60	**1.71**	0.02	0.90	**1.99**	0.02
i320-032	3.51	0.00	0.60	**2.20**	0.00	0.30	**1.83**	0.00	0.70	**3.14**	0.02	0.90	3.86	0.02	0.60	**1.74**	0.02
i320-033	3.86	0.00	0.50	4.90	0.00	0.90	4.55	0.00	0.40	4.70	0.02	0.70	**3.47**	0.02	0.40	**3.50**	0.02
i320-034	9.09	0.00	0.50	**5.03**	0.00	0.30	**4.82**	0.00	0.20	**3.11**	0.02	0.30	**2.16**	0.02	0.20	**2.81**	0.02
i320-035	8.67	0.00	0.10	**1.79**	0.00	0.20	**5.75**	0.00	0.20	**3.14**	0.02	0.20	**5.81**	0.02	0.10	**4.96**	0.02
i320-041	24.39	0.01	0.40	**17.75**	0.01	0.50	**6.46**	0.01	0.20	**9.75**	0.07	0.10	**10.62**	0.07	0.80	**15.52**	0.08
i320-042	23.65	0.01	0.90	**7.48**	0.01	0.90	**8.22**	0.01	0.90	**21.55**	0.07	0.10	**8.05**	0.07	0.50	**16.07**	0.08
i320-043	12.69	0.01	0.80	13.81	0.01	0.50	13.81	0.01	0.90	13.59	0.07	0.40	**5.37**	0.07	0.70	**6.59**	0.08
i320-044	23.74	0.01	0.90	23.74	0.01	0.70	23.74	0.01	0.70	23.74	0.07	0.80	**6.66**	0.07	0.80	**8.05**	0.08
i320-045	10.52	0.01	0.90	**10.15**	0.01	0.40	12.95	0.01	0.90	10.76	0.07	0.30	**10.42**	0.07	0.80	**8.47**	0.08
i320-101	4.73	0.00	0.50	**3.52**	0.00	0.30	**4.34**	0.00	0.50	4.75	0.02	0.70	**3.29**	0.02	0.60	4.84	0.02
i320-102	3.13	0.00	0.70	3.20	0.00	0.10	**2.87**	0.00	0.90	3.64	0.02	0.10	**2.26**	0.02	0.60	3.22	0.02
i320-103	3.83	0.00	0.90	**2.21**	0.00	0.20	**2.51**	0.00	0.50	**1.99**	0.02	0.50	**2.03**	0.02	0.50	**2.11**	0.02
i320-104	8.65	0.00	0.80	9.01	0.00	0.10	**8.17**	0.00	0.10	8.71	0.02	0.70	**6.59**	0.02	0.50	**6.42**	0.02
i320-105	3.24	0.00	0.60	**2.04**	0.00	0.80	**2.79**	0.00	0.00	**2.11**	0.02	0.80	**2.79**	0.02	0.70	**2.11**	0.02
i320-111	5.97	0.01	0.80	**5.05**	0.01	0.60	**5.08**	0.01	0.90	6.09	0.03	0.90	**5.49**	0.03	0.70	6.18	0.03
i320-112	5.96	0.01	0.80	7.33	0.01	0.80	6.60	0.01	0.80	7.34	0.03	0.80	6.37	0.03	0.90	6.43	0.03
i320-113	6.91	0.01	0.80	**6.23**	0.01	0.50	**3.58**	0.01	0.50	8.11	0.03	0.10	**6.21**	0.03	0.20	**5.51**	0.03
i320-114	8.29	0.01	0.60	9.57	0.01	0.90	**4.82**	0.01	0.90	8.68	0.03	0.40	**5.30**	0.03	0.50	**3.71**	0.03
i320-115	9.54	0.01	0.80	**7.93**	0.01	0.90	9.97	0.01	0.70	**8.82**	0.03	0.90	**7.05**	0.03	0.80	**6.51**	0.03
i320-121	39.51	0.04	0.10	39.51	0.04	0.30	39.51	0.05	0.10	39.51	0.31	0.10	39.51	0.30	0.10	39.51	0.32
i320-122	40.98	0.04	0.10	40.98	0.04	0.20	40.98	0.05	0.10	40.98	0.31	0.10	40.98	0.30	0.10	40.98	0.32
i320-123	39.47	0.04	0.10	39.47	0.04	0.10	39.47	0.05	0.10	39.47	0.31	0.10	39.47	0.30	0.10	39.47	0.32
i320-124	40.02	0.04	0.10	40.02	0.04	0.30	40.02	0.05	0.10	40.02	0.30	0.10	40.02	0.30	0.10	40.02	0.32
i320-125	39.70	0.04	0.10	39.70	0.04	0.20	39.70	0.05	0.10	39.70	0.31	0.10	39.70	0.30	0.10	39.70	0.32
i320-131	7.22	0.00	0.90	8.55	0.00	0.80	**6.47**	0.01	0.00	**6.12**	0.02	0.80	**4.23**	0.02	0.40	**3.11**	0.02
i320-132	6.82	0.00	0.80	**6.47**	0.00	0.90	7.94	0.01	0.60	**5.67**	0.02	0.40	**4.41**	0.02	0.20	**5.56**	0.02
i320-133	7.47	0.00	0.30	**6.01**	0.00	0.10	**4.62**	0.01	0.40	**6.09**	0.02	0.70	**3.94**	0.02	0.80	**3.25**	0.02
i320-134	9.12	0.00	0.60	**7.28**	0.00	0.50	**7.89**	0.01	0.10	**7.76**	0.02	0.30	**4.43**	0.02	0.70	**4.14**	0.02
i320-135	4.61	0.00	0.90	4.77	0.00	0.80	**4.24**	0.01	0.90	**4.19**	0.02	0.80	5.98	0.02	0.60	**4.08**	0.02
i320-141	32.09	0.01	0.90	32.42	0.02	0.90	32.20	0.02	0.90	32.20	0.08	0.80	**5.96**	0.08	0.70	**9.28**	0.08
i320-142	33.19	0.01	0.90	33.25	0.02	0.80	33.25	0.02	0.90	33.19	0.08	0.50	**9.55**	0.08	0.70	**9.16**	0.08
i320-143	29.46	0.01	0.90	29.99	0.02	0.70	**16.71**	0.02	0.90	29.90	0.08	0.40	**7.87**	0.08	0.70	**9.68**	0.08
i320-144	34.82	0.01	0.90	34.88	0.02	0.90	34.82	0.02	0.90	34.82	0.08	0.00	**7.36**	0.08	0.70	**10.89**	0.08
i320-145	31.85	0.01	0.90	32.10	0.02	0.90	31.91	0.02	0.90	31.88	0.08	0.00	**5.62**	0.08	0.70	**9.72**	0.08

Table S15. (Continued) Results of the benchmark problem set I320 by using the SPH. Texts with a gray background are complete graph instances.

	without centrality		with centrality															
			degree			eigenvector			closeness			vertex betweenness			edge betweenness			
name	gap	time	α	gap	time	α	gap	time	α	gap	time	α	gap	time	α	gap	time	
i320-201	7.63	0.01	0.70	**5.48**	0.01	0.40	**6.40**	0.01	0.90	**5.15**	0.02	0.70	**3.64**	0.02	0.50	**4.95**	0.02	
i320-202	5.51	0.01	0.80	**2.20**	0.01	0.80	**4.94**	0.01	0.10	**4.23**	0.02	0.40	**3.11**	0.02	0.30	**2.94**	0.02	
i320-203	4.81	0.01	0.20	**4.46**	0.01	0.90	**4.71**	0.01	0.90	5.41	0.02	0.80	**4.33**	0.02	0.70	**4.79**	0.02	
i320-204	5.47	0.01	0.40	**5.04**	0.01	0.10	**4.54**	0.01	0.70	**4.53**	0.02	0.70	**4.50**	0.02	0.20	**3.54**	0.02	
i320-205	5.11	0.01	0.10	**4.51**	0.01	0.90	**4.81**	0.01	0.70	**4.83**	0.02	0.90	**4.72**	0.02	0.20	**4.04**	0.02	
i320-211	8.83	0.01	0.80	10.48	0.01	0.90	9.03	0.01	0.60	**8.67**	0.03	0.90	**7.64**	0.04	0.90	**7.41**	0.04	
i320-212	9.77	0.01	0.80	**8.24**	0.01	0.70	**8.27**	0.01	0.40	10.58	0.03	0.20	**8.32**	0.04	0.20	**7.16**	0.04	
i320-213	9.60	0.01	0.80	12.29	0.01	0.90	10.20	0.01	0.90	13.24	0.03	0.30	**7.23**	0.04	0.10	**6.87**	0.04	
i320-214	10.18	0.01	0.90	11.39	0.01	0.50	10.69	0.01	0.90	13.52	0.03	0.30	**6.74**	0.04	0.20	**7.53**	0.04	
i320-215	9.82	0.01	0.60	**8.83**	0.01	0.70	10.51	0.01	0.60	9.94	0.03	0.10	**7.71**	0.04	0.40	**7.50**	0.04	
i320-221	42.91	0.05	0.10	42.91	0.06	0.30	42.91	0.06	0.10	42.91	0.32	0.10	42.91	0.31	0.10	42.91	0.33	
i320-222	42.88	0.05	0.10	42.88	0.06	0.20	42.88	0.06	0.10	42.88	0.32	0.10	42.88	0.31	0.10	42.88	0.33	
i320-223	42.63	0.05	0.10	42.63	0.06	0.30	42.63	0.06	0.10	42.63	0.32	0.10	42.63	0.31	0.10	42.63	0.33	
i320-224	42.65	0.05	0.10	42.65	0.06	0.40	42.65	0.06	0.10	42.65	0.32	0.10	42.65	0.31	0.10	42.65	0.33	
i320-225	42.94	0.05	0.10	42.94	0.05	0.20	42.94	0.06	0.10	42.94	0.32	0.10	42.94	0.31	0.10	42.94	0.33	
i320-231	5.62	0.01	0.30	5.62	0.01	0.40	**4.60**	0.01	0.60	**5.03**	0.02	0.70	**4.91**	0.02	0.90	**4.79**	0.02	
i320-232	7.36	0.01	0.10	**3.69**	0.01	0.90	**6.71**	0.01	0.20	**6.02**	0.02	0.50	**5.75**	0.02	0.70	**4.94**	0.02	
i320-233	6.86	0.01	0.90	6.92	0.01	0.60	**5.57**	0.01	0.90	7.91	0.02	0.90	**6.82**	0.02	0.80	7.30	0.02	
i320-234	7.57	0.01	0.40	**6.70**	0.01	0.50	**6.46**	0.01	0.90	**6.94**	0.02	0.50	**5.49**	0.02	0.50	**5.78**	0.02	
i320-235	6.73	0.01	0.40	**3.44**	0.01	0.20	**3.96**	0.01	0.20	**3.26**	0.02	0.40	**4.98**	0.02	0.40	**4.65**	0.02	
i320-241	37.77	0.02	0.90	37.88	0.03	0.90	37.77	0.03	0.10	37.77	0.08	0.40	**5.21**	0.09	0.70	**6.83**	0.09	
i320-242	37.60	0.02	0.90	37.71	0.03	0.90	37.64	0.03	0.10	37.60	0.08	0.20	**4.54**	0.09	0.70	**7.23**	0.09	
i320-243	37.31	0.02	0.90	37.44	0.03	0.90	37.31	0.03	0.10	37.31	0.08	0.20	**4.60**	0.09	0.70	**5.89**	0.09	
i320-244	37.21	0.02	0.90	37.27	0.03	0.90	37.21	0.03	0.10	37.21	0.08	0.30	**4.83**	0.09	0.70	**5.76**	0.09	
i320-245	37.87	0.02	0.90	37.92	0.03	0.90	37.87	0.03	0.10	37.87	0.08	0.20	**5.28**	0.09	0.70	**7.24**	0.09	
i320-301	3.73	0.01	0.60	**2.75**	0.01	0.60	**3.24**	0.02	0.90	**2.88**	0.03	0.30	**2.07**	0.03	0.30	**3.41**	0.03	
i320-302	2.97	0.01	0.90	3.55	0.01	0.80	**2.81**	0.02	0.90	4.38	0.03	0.90	**2.44**	0.03	0.90	3.20	0.03	
i320-303	3.58	0.01	0.30	**3.25**	0.01	0.80	**2.32**	0.02	0.90	3.77	0.03	0.60	**2.05**	0.03	0.50	**2.90**	0.03	
i320-304	3.96	0.01	0.50	**2.71**	0.01	0.10	**3.04**	0.02	0.20	**3.20**	0.03	0.50	**2.02**	0.03	0.20	**1.63**	0.03	
i320-305	5.28	0.01	0.90	**3.42**	0.01	0.10	**3.52**	0.02	0.20	**3.67**	0.03	0.20	**2.45**	0.03	0.10	**2.30**	0.03	
i320-311	22.46	0.03	0.90	24.41	0.03	0.40	23.97	0.03	0.90	23.90	0.05	0.40	**6.82**	0.05	0.30	**6.58**	0.05	
i320-312	27.12	0.03	0.90	27.72	0.03	0.90	**24.43**	0.03	0.90	27.72	0.05	0.10	**7.78**	0.05	0.10	**6.79**	0.05	
i320-313	28.70	0.03	0.70	**26.34**	0.03	0.80	**25.90**	0.03	0.90	**28.38**	0.05	0.10	**5.61**	0.05	0.20	**5.82**	0.05	
i320-314	21.84	0.03	0.40	**20.34**	0.03	0.60	21.95	0.03	0.50	**20.70**	0.05	0.20	**6.98**	0.05	0.50	**6.55**	0.05	
i320-315	29.68	0.03	0.90	**29.43**	0.03	0.70	**28.09**	0.03	0.10	**29.41**	0.05	0.10	**6.97**	0.05	0.30	**6.81**	0.05	
i320-321	45.28	0.08	0.10	45.28	0.09	0.30	45.28	0.09	0.10	45.28	0.35	0.10	45.28	0.35	0.10	45.28	0.37	
i320-322	45.19	0.08	0.10	45.19	0.09	0.30	45.19	0.09	0.10	45.19	0.35	0.10	45.19	0.35	0.10	45.19	0.36	
i320-323	45.41	0.08	0.10	45.41	0.09	0.40	45.41	0.10	0.10	45.41	0.35	0.10	45.41	0.35	0.10	45.41	0.36	
i320-324	45.45	0.08	0.10	45.45	0.09	0.30	45.45	0.09	0.10	45.45	0.35	0.10	45.45	0.35	0.10	45.45	0.36	
i320-325	45.41	0.08	0.10	45.41	0.09	0.40	45.41	0.10	0.10	45.41	0.35	0.10	45.41	0.35	0.10	45.41	0.36	
i320-331	8.37	0.02	0.60	9.39	0.02	0.40	**7.66**	0.02	0.90	8.65	0.03	0.40	**4.95**	0.03	0.10	**4.73**	0.04	
i320-332	8.02	0.02	0.90	**5.87**	0.02	0.10	**5.88**	0.02	0.90	**6.78**	0.03	0.50	**4.54**	0.04	0.40	**4.64**	0.04	
i320-333	8.60	0.02	0.90	**8.20**	0.02	0.50	**6.46**	0.02	0.80	9.17	0.03	0.10	**6.59**	0.03	0.20	**5.49**	0.04	
i320-334	6.58	0.02	0.90	8.15	0.02	0.80	7.49	0.02	0.90	8.06	0.03	0.70	**5.74**	0.03	0.70	**5.58**	0.04	
i320-335	4.80	0.02	0.40	6.32	0.02	0.60	5.08	0.02	0.70	6.36	0.03	0.70	**4.26**	0.03	0.20	**3.18**	0.04	
i320-341	41.45	0.05	0.90	41.59	0.06	0.90	41.46	0.06	0.80	41.46	0.11	0.60	**2.34**	0.12	0.70	**3.11**	0.12	
i320-342	42.11	0.05	0.90	42.18	0.06	0.90	42.18	0.06	0.80	42.12	0.11	0.70	**2.29**	0.12	0.70	**2.84**	0.12	
i320-343	41.38	0.05	0.90	41.52	0.06	0.90	41.45	0.06	0.90	41.40	0.11	0.40	**2.55**	0.12	0.80	**3.34**	0.12	
i320-344	41.59	0.05	0.90	41.83	0.06	0.90	41.69	0.06	0.90	41.64	0.11	0.40	**2.15**	0.12	0.70	**3.34**	0.12	
i320-345	41.98	0.05	0.90	42.07	0.05	0.90	42.01	0.06	0.90	41.98	0.11	0.10	**1.88**	0.11	0.70	**3.09**	0.12	
average	19.27	0.02	-	**18.76** (-0.51)	0.02	-	**18.36** (-0.91)	0.02	-	**18.85** (-0.42)	0.10	-	**11.93** (-7.34)	0.10	-	**12.33** (-6.94)	0.10	

Table S16. Results of the benchmark problem set I640 by using the SPH. Texts with a gray background are complete graph instances.

	without centrality		with centrality															
			degree			eigenvector			closeness			vertex betweenness			edge betweenness			
name	gap	time	α	gap	time	α	gap	time	α	gap	time	α	gap	time	α	gap	time	
i640-001	7.55	0.01	0.60	**4.79**	0.01	0.40	9.58	0.05	0.80	**6.61**	0.13	0.40	**5.54**	0.14	0.50	**2.47**	0.14	
i640-002	6.73	0.01	0.30	**5.60**	0.01	0.80	**4.62**	0.05	0.20	**6.04**	0.12	0.90	**4.43**	0.14	0.00	**1.36**	0.14	
i640-003	4.63	0.01	0.70	**4.28**	0.01	0.60	**3.27**	0.06	0.80	**3.94**	0.12	0.70	**3.72**	0.14	0.60	**2.74**	0.15	
i640-004	4.58	0.01	0.60	**3.48**	0.01	0.10	4.61	0.04	0.30	**3.84**	0.12	0.40	**4.00**	0.14	0.70	**4.32**	0.14	
i640-005	5.92	0.01	0.00	**3.03**	0.01	0.10	**5.45**	0.04	0.40	**4.11**	0.12	0.20	**1.07**	0.14	0.10	**1.16**	0.14	
i640-011	9.97	0.02	0.90	9.97	0.02	0.30	10.34	0.05	0.90	10.20	0.21	0.80	**9.39**	0.23	0.90	**9.54**	0.23	
i640-012	9.88	0.02	0.70	**3.28**	0.02	0.90	11.25	0.05	0.90	**8.84**	0.20	0.80	**6.03**	0.23	0.80	**4.35**	0.23	
i640-013	10.09	0.02	0.50	**9.05**	0.02	0.70	10.53	0.05	0.00	**8.90**	0.21	0.30	**7.83**	0.23	0.30	**8.44**	0.23	
i640-014	12.57	0.02	0.90	12.57	0.02	0.30	**12.22**	0.05	0.80	**11.23**	0.21	0.10	**9.10**	0.22	0.20	**7.88**	0.23	
i640-015	4.86	0.02	0.90	4.86	0.02	0.90	**4.25**	0.06	0.10	6.10	0.21	0.40	5.54	0.23	0.40	5.68	0.24	
i640-021	34.36	0.19	0.10	34.36	0.20	0.10	34.36	0.28	0.10	34.36	5.73	0.10	34.36	5.38	0.10	34.36	5.48	
i640-022	33.77	0.17	0.10	33.77	0.17	0.20	33.77	0.23	0.10	33.77	5.37	0.10	33.77	5.05	0.10	33.77	5.02	
i640-023	33.87	0.20	0.10	33.87	0.20	0.10	33.87	0.28	0.10	33.87	5.77	0.10	33.87	5.40	0.10	33.87	5.50	
i640-024	33.87	0.20	0.10	33.87	0.20	0.40	33.87	0.27	0.10	33.87	5.68	0.10	33.87	5.38	0.10	33.87	5.51	
i640-025	34.73	0.20	0.10	34.73	0.20	0.20	34.73	0.28	0.10	34.73	5.65	0.10	34.73	5.34	0.10	34.73	5.47	
i640-031	7.46	0.01	0.80	**4.96**	0.01	0.90	**4.90**	0.07	0.90	**2.66**	0.14	0.90	**4.30**	0.16	0.80	**3.25**	0.16	
i640-032	11.17	0.01	0.40	**7.63**	0.01	0.20	**9.54**	0.05	0.00	**7.10**	0.14	0.10	**6.91**	0.16	0.10	**3.29**	0.16	
i640-033	3.53	0.01	0.00	**2.13**	0.01	0.10	**2.95**	0.07	0.20	**3.48**	0.14	0.10	**2.00**	0.15	0.20	**0.46**	0.16	
i640-034	7.16	0.01	0.40	**6.97**	0.01	0.80	**3.86**	0.05	0.70	7.21	0.14	0.60	**3.66**	0.15	0.80	**3.14**	0.16	
i640-035	2.40	0.01	0.90	2.40	0.01	0.80	**1.82**	0.08	0.90	**1.61**	0.14	0.70	**1.58**	0.16	0.90	**1.47**	0.16	
i640-041	27.62	0.06	0.10	**19.24**	0.05	0.30	**15.71**	0.08	0.90	27.76	1.07	0.10	**11.60**	1.02	0.70	**17.02**	1.05	
i640-042	15.90	0.06	0.20	**5.67**	0.05	0.60	**7.16**	0.09	0.50	**12.12**	1.07	0.80	**4.04**	1.02	0.60	**10.53**	1.06	
i640-043	24.96	0.06	0.00	24.96	0.05	0.00	24.96	0.07	0.00	24.96	1.04	0.70	**7.61**	1.01	0.70	**11.18**	1.04	
i640-044	15.82	0.06	0.40	**7.46**	0.05	0.50	**6.35**	0.08	0.40	16.55	1.05	0.60	**7.69**	1.03	0.70	**12.86**	1.06	
i640-045	9.16	0.06	0.80	**8.65**	0.06	0.90	**7.25**	0.09	0.60	**8.95**	1.05	0.70	**8.32**	1.01	0.90	**7.63**	1.05	
i640-101	8.09	0.02	0.90	8.09	0.02	0.40	**7.99**	0.07	0.40	8.31	0.13	0.90	**8.03**	0.15	0.70	**7.99**	0.16	
i640-102	4.62	0.02	0.90	4.62	0.02	0.20	**4.32**	0.06	0.90	5.57	0.13	0.60	**3.52**	0.15	0.80	**3.85**	0.16	
i640-103	5.91	0.02	0.80	**5.35**	0.02	0.70	**5.42**	0.06	0.20	**4.85**	0.13	0.80	**5.48**	0.15	0.40	**4.45**	0.16	
i640-104	4.61	0.02	0.90	4.61	0.02	0.80	7.25	0.09	0.80	**4.41**	0.14	0.80	**3.67**	0.15	0.20	**3.17**	0.16	
i640-105	3.24	0.02	0.80	**3.12**	0.02	0.20	4.85	0.08	0.70	**2.93**	0.13	0.30	**3.21**	0.15	0.60	3.29	0.16	
i640-111	9.99	0.04	0.80	**9.38**	0.04	0.70	**8.21**	0.08	0.90	**9.39**	0.24	0.20	**7.51**	0.25	0.10	**6.34**	0.26	
i640-112	9.05	0.04	0.90	9.05	0.04	0.40	**8.43**	0.08	0.30	9.52	0.24	0.10	9.32	0.25	0.40	**8.93**	0.26	
i640-113	9.30	0.04	0.70	**8.44**	0.04	0.50	**8.00**	0.08	0.00	10.49	0.24	0.90	**8.84**	0.25	0.90	**8.61**	0.26	
i640-114	8.57	0.04	0.90	8.57	0.04	0.80	**7.03**	0.08	0.30	**8.49**	0.23	0.30	**7.41**	0.25	0.60	**6.86**	0.26	
i640-115	9.63	0.04	0.80	**8.96**	0.04	0.60	**6.97**	0.07	0.40	**9.39**	0.24	0.10	**8.37**	0.25	0.30	**7.78**	0.26	
i640-121	42.13	0.27	0.10	42.13	0.27	0.20	42.13	0.35	0.10	42.13	5.59	0.10	42.13	5.25	0.10	42.13	5.37	
i640-122	42.23	0.27	0.10	42.23	0.26	0.20	42.23	0.34	0.10	42.23	5.53	0.10	42.23	5.15	0.10	42.23	5.38	
i640-123	41.50	0.21	0.10	41.50	0.21	0.20	41.50	0.32	0.10	41.50	5.45	0.10	41.50	5.12	0.10	41.50	5.19	
i640-124	41.93	0.26	0.10	41.93	0.26	0.20	41.93	0.31	0.10	41.93	5.43	0.10	41.93	5.09	0.10	41.93	5.14	
i640-125	41.93	0.26	0.10	41.93	0.27	0.20	41.93	0.34	0.10	41.93	5.69	0.10	41.93	5.41	0.10	41.93	5.32	
i640-131	5.06	0.02	0.90	5.06	0.02	0.90	5.27	0.08	0.90	**4.99**	0.15	0.60	**4.90**	0.17	0.20	**4.75**	0.17	
i640-132	7.50	0.02	0.80	**7.15**	0.02	0.50	**6.92**	0.06	0.90	9.02	0.15	0.80	**6.88**	0.17	0.70	**6.70**	0.17	
i640-133	6.33	0.02	0.80	**5.46**	0.02	0.70	6.65	0.08	0.50	7.49	0.15	0.70	6.85	0.19	0.70	**5.67**	0.17	
i640-134	5.68	0.02	0.80	**5.56**	0.02	0.30	6.43	0.07	0.60	6.43	0.15	0.80	**3.99**	0.17	0.90	**3.65**	0.18	
i640-135	10.19	0.02	0.70	**8.87**	0.02	0.10	**8.56**	0.08	0.80	10.67	0.15	0.60	**8.45**	0.17	0.30	**7.38**	0.17	
i640-141	36.39	0.11	0.90	36.39	0.12	0.90	**36.37**	0.15	0.10	**36.37**	1.13	0.10	**5.74**	1.06	0.80	**6.71**	1.13	
i640-142	36.40	0.12	0.90	36.40	0.12	0.90	**36.24**	0.15	0.10	**36.24**	1.12	0.60	**5.82**	1.09	0.70	**9.72**	1.11	
i640-143	36.54	0.11	0.90	36.54	0.11	0.90	**36.48**	0.14	0.10	**36.48**	1.09	0.30	**6.75**	1.04	0.70	**10.83**	1.10	
i640-144	36.33	0.11	0.90	36.33	0.12	0.90	**36.29**	0.14	0.10	**36.29**	1.12	0.20	**6.00**	1.08	0.70	**10.90**	1.13	
i640-145	35.49	0.12	0.80	35.49	0.11	0.70	**35.47**	0.14	0.10	**35.45**	1.13	0.10	**7.28**	1.08	0.70	**9.67**	1.12	

Table S16. (Continued) Results of the benchmark problem set I640 by using the SPH. Texts with a gray background are complete graph instances.

	without centrality		with centrality														
			degree			eigenvector			closeness			vertex betweenness			edge betweenness		
name	gap	time	α	gap	time	α	gap	time	α	gap	time	α	gap	time	α	gap	time
i640-201	6.31	0.04	0.70	**5.76**	0.04	0.50	**4.88**	0.09	0.50	8.78	0.15	0.30	**1.34**	0.17	0.10	**3.48**	0.18
i640-202	4.89	0.04	0.90	4.89	0.04	0.10	**4.35**	0.08	0.90	**4.74**	0.15	0.80	**4.04**	0.18	0.90	**4.08**	0.18
i640-203	4.72	0.04	0.90	4.72	0.04	0.90	5.24	0.08	0.70	**4.40**	0.15	0.50	**2.50**	0.17	0.60	**2.24**	0.17
i640-204	5.45	0.04	0.80	**3.93**	0.04	0.90	6.22	0.10	0.00	6.13	0.15	0.10	**4.62**	0.17	0.50	**4.79**	0.17
i640-205	3.09	0.04	0.80	**2.72**	0.04	0.10	3.30	0.10	0.90	3.19	0.15	0.50	**2.39**	0.17	0.70	**2.79**	0.17
i640-211	13.72	0.07	0.60	**12.11**	0.07	0.50	**10.69**	0.10	0.00	**13.25**	0.26	0.90	**9.64**	0.28	0.40	**9.34**	0.29
i640-212	11.16	0.08	0.90	11.16	0.08	0.90	**10.27**	0.11	0.70	12.26	0.27	0.40	**10.10**	0.29	0.50	**10.10**	0.30
i640-213	10.06	0.08	0.80	**9.92**	0.08	0.50	**8.87**	0.11	0.40	10.60	0.27	0.10	**9.46**	0.29	0.10	**9.53**	0.30
i640-214	12.29	0.08	0.60	**11.75**	0.08	0.50	**10.84**	0.11	0.90	**11.94**	0.28	0.40	**9.52**	0.29	0.10	**8.13**	0.29
i640-215	12.02	0.08	0.50	**11.32**	0.08	0.70	**11.42**	0.12	0.10	13.75	0.27	0.70	**9.87**	0.29	0.20	**9.14**	0.30
i640-221	44.07	0.38	0.10	44.07	0.40	0.30	44.07	0.45	0.10	44.07	5.67	0.10	44.07	5.26	0.10	44.07	5.40
i640-222	44.36	0.39	0.10	44.36	0.39	0.30	44.36	0.45	0.10	44.36	5.68	0.10	44.36	5.31	0.10	44.36	5.33
i640-223	44.15	0.37	0.10	44.15	0.38	0.20	44.15	0.44	0.10	44.15	5.62	0.10	44.15	5.26	0.10	44.15	5.42
i640-224	44.41	0.29	0.10	44.41	0.29	0.30	44.41	0.39	0.10	44.41	5.56	0.10	44.41	5.23	0.10	44.41	5.31
i640-225	44.18	0.30	0.10	44.18	0.31	0.20	44.18	0.36	0.10	44.18	5.56	0.10	44.18	5.24	0.10	44.18	5.32
i640-231	4.90	0.05	0.90	4.90	0.05	0.90	5.20	0.10	0.90	7.27	0.17	0.90	**4.21**	0.19	0.90	5.59	0.19
i640-232	8.22	0.03	0.70	**7.29**	0.03	0.10	**5.99**	0.10	0.90	**6.91**	0.17	0.70	**6.06**	0.19	0.20	**4.99**	0.19
i640-233	11.69	0.04	0.40	**6.36**	0.05	0.70	**5.64**	0.09	0.90	**9.16**	0.17	0.80	**5.03**	0.19	0.70	**6.66**	0.20
i640-234	7.24	0.05	0.60	**4.44**	0.05	0.90	**6.72**	0.10	0.50	**6.38**	0.17	0.60	**5.87**	0.19	0.70	**6.08**	0.19
i640-235	7.22	0.04	0.60	**6.26**	0.05	0.70	**6.26**	0.10	0.90	8.66	0.17	0.70	**5.94**	0.21	0.70	**5.34**	0.20
i640-241	40.09	0.20	0.90	40.09	0.20	0.90	**39.99**	0.23	0.10	**39.98**	1.21	0.60	**3.84**	1.18	0.80	**5.48**	1.23
i640-242	40.69	0.20	0.90	40.69	0.20	0.90	**40.63**	0.23	0.10	**40.62**	1.24	0.70	**4.44**	1.17	0.70	**6.82**	1.22
i640-243	40.71	0.20	0.90	40.71	0.20	0.90	**40.64**	0.22	0.10	**40.62**	1.15	0.70	**3.65**	1.11	0.70	**6.69**	1.18
i640-244	39.96	0.20	0.90	39.96	0.20	0.90	**39.94**	0.23	0.10	**39.91**	1.22	0.80	**3.38**	1.18	0.70	**6.95**	1.24
i640-245	40.46	0.18	0.90	40.46	0.18	0.90	**40.44**	0.23	0.10	**40.39**	1.20	0.40	**3.79**	1.18	0.70	**6.32**	1.23
i640-301	4.65	0.12	0.90	4.65	0.13	0.90	**3.53**	0.19	0.30	6.27	0.24	0.70	**3.74**	0.26	0.90	**3.69**	0.27
i640-302	3.61	0.13	0.80	**3.52**	0.12	0.10	3.87	0.19	0.90	3.77	0.24	0.40	**2.07**	0.26	0.30	**3.20**	0.27
i640-303	3.75	0.13	0.90	3.75	0.13	0.20	3.98	0.21	0.80	3.81	0.24	0.70	**2.37**	0.27	0.30	**2.68**	0.27
i640-304	4.55	0.10	0.90	4.55	0.10	0.50	4.77	0.19	0.40	6.50	0.24	0.60	**3.19**	0.27	0.40	**2.49**	0.28
i640-305	6.30	0.13	0.50	**6.28**	0.13	0.20	**5.04**	0.19	0.10	**6.13**	0.24	0.10	**3.78**	0.28	0.10	**3.32**	0.27
i640-311	23.55	0.26	0.90	23.55	0.26	0.70	**23.43**	0.30	0.90	26.06	0.47	0.10	**6.35**	0.47	0.10	**5.38**	0.48
i640-312	25.30	0.26	0.90	25.30	0.26	0.20	**24.07**	0.28	0.90	26.10	0.47	0.40	**7.59**	0.47	0.20	**7.03**	0.47
i640-313	24.31	0.26	0.90	24.31	0.26	0.80	**21.71**	0.29	0.90	24.38	0.46	0.10	**7.64**	0.47	0.10	**6.03**	0.47
i640-314	26.83	0.25	0.90	26.83	0.26	0.80	26.86	0.29	0.60	26.86	0.46	0.40	**5.64**	0.48	0.20	**4.61**	0.47
i640-315	29.51	0.26	0.90	29.51	0.26	0.80	**28.91**	0.29	0.70	**29.42**	0.44	0.10	**6.97**	0.46	0.20	**6.12**	0.44
i640-321	46.53	0.86	0.10	46.53	0.86	0.30	46.53	0.88	0.10	46.53	6.13	0.10	46.53	5.74	0.10	46.53	5.85
i640-322	46.77	0.87	0.10	46.77	0.87	0.30	46.77	0.90	0.10	46.77	6.06	0.10	46.77	5.74	0.10	46.77	5.88
i640-323	46.60	0.81	0.10	46.60	0.82	0.30	46.60	0.88	0.10	46.60	5.87	0.10	46.60	5.54	0.10	46.60	5.69
i640-324	46.68	0.85	0.10	46.68	0.86	0.30	46.68	0.88	0.10	46.68	5.94	0.10	46.68	5.46	0.10	46.68	5.46
i640-325	46.74	0.80	0.10	46.74	0.80	0.40	46.74	0.85	0.10	46.74	5.75	0.10	46.74	5.57	0.10	46.74	5.73
i640-331	5.49	0.16	0.40	**5.42**	0.16	0.70	**5.28**	0.24	0.90	7.37	0.28	0.70	**4.52**	0.29	0.50	**4.28**	0.27
i640-332	8.14	0.16	0.70	**7.28**	0.16	0.90	**7.42**	0.23	0.60	8.46	0.28	0.10	**4.55**	0.32	0.40	**4.14**	0.31
i640-333	6.60	0.15	0.60	**5.42**	0.15	0.50	**5.60**	0.21	0.60	7.30	0.26	0.30	**5.08**	0.28	0.70	**4.95**	0.28
i640-334	7.84	0.12	0.20	**7.61**	0.12	0.60	**5.71**	0.19	0.90	7.86	0.25	0.10	**4.23**	0.27	0.60	**4.06**	0.27
i640-335	7.09	0.15	0.80	**6.97**	0.15	0.90	**6.66**	0.21	0.90	8.08	0.28	0.40	**4.76**	0.30	0.60	**4.56**	0.31
i640-341	44.10	0.55	0.90	44.10	0.55	0.90	**44.00**	0.52	0.10	**43.93**	1.44	0.80	**1.30**	1.40	0.70	**2.34**	1.53
i640-342	44.30	0.54	0.90	44.30	0.55	0.90	**44.27**	0.58	0.10	**44.23**	1.55	0.10	**1.53**	1.52	0.80	**2.00**	1.58
i640-343	44.11	0.45	0.90	44.11	0.46	0.90	**44.01**	0.48	0.10	**43.95**	1.44	0.10	**1.40**	1.37	0.70	**2.38**	1.45
i640-344	44.08	0.55	0.90	44.08	0.55	0.90	**44.02**	0.56	0.10	**43.95**	1.54	0.30	**1.27**	1.52	0.80	**2.18**	1.55
i640-345	44.41	0.55	0.90	44.41	0.55	0.90	**44.35**	0.60	0.10	**44.30**	1.58	0.40	**1.38**	1.55	0.80	**1.94**	1.50
average	20.59	0.17	-	**19.85**	0.17	-	**19.84**	0.21	-	**20.57**	1.51	-	**12.66**	1.44	-	**12.93**	1.47
				(-0.74)			(-0.75)			(-0.02)			(-7.93)			(-7.66)	

Table S17. Results of the benchmark problem set B by using the ADH.

	without centrality		with centrality														
			degree			eigenvector			closeness			vertex betweenness			edge betweenness		
name	gap	time	α	gap	time	α	gap	time	α	gap	time	α	gap	time	α	gap	time
b01	0.00	0.00	0.70	0.00	0.00	0.50	0.00	0.00	0.80	0.00	0.00	0.10	0.00	0.00	0.00	0.00	0.00
b02	1.16	0.00	0.90	**0.00**	0.00	0.40	**0.00**	0.00	0.70	**0.00**	0.00	0.10	**0.00**	0.00	0.10	**0.00**	0.00
b03	0.00	0.00	0.80	0.00	0.00	0.10	0.00	0.00	0.70	0.00	0.00	0.30	0.00	0.00	0.10	0.00	0.00
b04	0.00	0.00	0.80	5.08	0.00	0.80	5.08	0.00	0.80	5.08	0.00	0.70	0.00	0.00	0.00	0.00	0.00
b05	0.00	0.00	0.80	0.00	0.00	0.80	0.00	0.00	0.20	1.64	0.00	0.40	0.00	0.00	0.10	0.00	0.00
b06	1.64	0.00	0.70	3.28	0.00	0.70	3.28	0.00	0.60	7.38	0.00	0.30	**0.00**	0.00	0.30	**0.00**	0.00
b07	0.00	0.00	0.80	0.00	0.00	0.50	0.00	0.00	0.60	0.00	0.00	0.00	0.90	0.00	0.00	0.90	0.00
b08	0.00	0.00	0.10	0.00	0.00	0.10	0.00	0.00	0.60	0.00	0.00	0.10	0.00	0.00	0.10	0.00	0.00
b09	0.45	0.01	0.60	0.45	0.01	0.50	**0.00**	0.01	0.60	**0.00**	0.01	0.50	0.45	0.01	0.30	0.45	0.01
b10	4.65	0.00	0.80	4.65	0.00	0.90	**0.00**	0.00	0.80	**0.00**	0.00	0.60	4.65	0.00	0.00	**0.00**	0.00
b11	2.27	0.00	0.50	2.27	0.00	0.80	2.27	0.00	0.80	2.27	0.00	0.30	**0.00**	0.00	0.00	**0.00**	0.00
b12	0.57	0.01	0.70	0.57	0.01	0.70	1.72	0.01	0.70	0.57	0.01	0.50	1.15	0.01	0.10	1.15	0.01
b13	6.06	0.00	0.20	**2.42**	0.00	0.50	**0.00**	0.00	0.10	**2.42**	0.00	0.10	**2.42**	0.01	0.00	**5.45**	0.01
b14	0.43	0.01	0.80	0.43	0.01	0.50	0.43	0.01	0.10	0.43	0.01	0.90	0.43	0.01	0.60	0.43	0.01
b15	0.94	0.02	0.60	**0.63**	0.02	0.40	0.94	0.02	0.50	**0.63**	0.02	0.90	0.94	0.02	0.80	0.94	0.02
b16	0.00	0.00	0.60	0.00	0.00	0.70	0.00	0.01	0.50	0.00	0.01	0.20	0.00	0.01	0.10	0.00	0.01
b17	0.76	0.01	0.70	1.53	0.01	0.90	0.76	0.01	0.80	1.53	0.01	0.10	0.76	0.01	0.20	0.76	0.01
b18	1.04	0.02	0.80	2.29	0.02	0.40	**0.46**	0.02	0.90	**0.46**	0.02	0.50	**0.92**	0.02	0.10	**0.92**	0.02
average	1.11	0.00	-	1.31	0.00	-	**0.83**	0.01	-	1.25	0.01	-	**0.70**	0.01	-	**0.61**	0.01
				(0.20)			(-0.28)			(0.14)			(-0.41)			(-0.50)	

Table S18. Results of the benchmark problem set C by using the ADH.

	without centrality		with centrality														
			degree			eigenvector			closeness			vertex betweenness			edge betweenness		
name	gap	time	α	gap	time	α	gap	time	α	gap	time	α	gap	time	α	gap	time
c01	4.71	0.14	0.50	**0.00**	0.14	0.10	**0.00**	0.14	0.30	**2.35**	0.16	0.20	**0.00**	0.19	0.20	4.71	0.19
c02	2.78	0.14	0.60	2.78	0.14	0.10	2.78	0.14	0.40	2.78	0.16	0.10	2.78	0.18	0.10	2.78	0.18
c03	1.88	0.37	0.60	3.85	0.38	0.90	3.58	0.37	0.90	2.92	0.40	0.90	**1.59**	0.41	0.70	2.12	0.41
c04	0.74	0.69	0.90	**0.65**	0.70	0.40	0.83	0.70	0.90	**0.46**	0.73	0.60	**0.46**	0.74	0.70	**0.46**	0.74
c05	1.18	2.44	0.90	**0.38**	2.52	0.40	**0.82**	2.52	0.90	**0.67**	2.54	0.60	**0.44**	2.58	0.10	1.27	2.56
c06	1.82	0.23	0.30	**0.00**	0.21	0.10	**0.00**	0.22	0.40	**0.00**	0.23	0.10	3.64	0.28	0.10	3.64	0.28
c07	4.90	0.23	0.70	**0.00**	0.22	0.70	**3.92**	0.23	0.80	**0.00**	0.26	0.30	**3.92**	0.28	0.10	4.90	0.28
c08	2.95	0.47	0.90	3.34	0.48	0.90	**2.75**	0.48	0.90	3.73	0.51	0.40	**2.75**	0.53	0.10	3.14	0.53
c09	2.77	0.78	0.90	**2.40**	0.80	0.60	**2.40**	0.82	0.90	4.38	0.84	0.50	**2.69**	0.85	0.40	3.54	0.85
c10	0.87	2.56	0.90	1.01	2.68	0.60	1.01	2.68	0.90	1.46	2.72	0.70	**0.73**	2.73	0.10	**0.82**	2.74
c11	6.25	0.47	0.80	**0.00**	0.43	0.80	**0.00**	0.45	0.90	**0.00**	0.50	0.20	**3.12**	0.55	0.10	**3.12**	0.59
c12	2.17	0.46	0.90	2.17	0.45	0.90	2.17	0.47	0.80	6.52	0.47	0.10	2.17	0.54	0.00	2.17	0.63
c13	2.41	0.69	0.90	**2.33**	0.70	0.90	3.49	0.71	0.90	3.49	0.75	0.30	2.71	0.79	0.10	4.65	0.82
c14	3.94	0.99	0.90	**2.17**	1.02	0.90	4.95	1.03	0.90	**3.10**	1.06	0.40	**3.72**	1.13	0.10	**3.41**	1.14
c15	1.46	2.71	0.90	**0.89**	2.91	0.90	**0.72**	2.91	0.90	**0.72**	2.95	0.10	**0.90**	2.97	0.10	**1.26**	3.00
c16	3.45	0.94	0.70	18.18	0.73	0.70	18.18	0.73	0.80	9.09	0.92	0.60	**0.00**	1.12	0.10	9.09	1.20
c17	5.56	0.94	0.90	5.56	0.90	0.90	5.56	0.91	0.90	5.56	1.03	0.20	**0.00**	1.10	0.10	16.67	1.21
c18	9.33	1.16	0.90	**7.08**	1.14	0.90	**7.96**	1.15	0.90	**9.29**	1.27	0.20	9.73	1.37	0.00	9.73	1.46
c19	11.88	1.45	0.90	**10.51**	1.47	0.90	**9.59**	1.48	0.80	**10.96**	1.50	0.10	**8.22**	1.68	0.10	**7.53**	1.79
c20	8.22	3.07	0.60	**4.49**	3.11	0.80	**4.49**	3.28	0.50	**4.49**	3.13	0.30	**4.49**	3.55	0.10	**4.49**	3.64
average	3.96	1.00	-	**3.39**	1.01	-	**3.76**	1.02	-	**3.60**	1.06	-	**2.70**	1.12	-	4.47	1.15
				(-0.57)			(-0.20)			(-0.36)			(-1.26)			(0.51)	

Table S19. Results of the benchmark problem set D by using the ADH.

	without		with centrality															
	centrality			degree			eigenvector			closeness			vertex betweenness			edge betweenness	
name	gap	time	α	gap	time	α	gap	time	α	gap	time	α	gap	time	α	gap	time
d01	7.55	0.94	0.50	**3.77**	0.96	0.10	**3.77**	0.97	0.80	**3.77**	1.08	0.10	**3.77**	1.29	0.10	**3.77**	1.16
d02	0.00	0.91	0.80	0.00	0.95	0.10	0.00	0.94	0.80	0.00	1.07	0.50	0.00	1.13	0.20	0.00	1.24
d03	2.08	3.17	0.80	**1.15**	3.27	0.40	**1.79**	3.30	0.50	**1.41**	3.34	0.50	**1.92**	3.46	0.70	2.17	3.47
d04	0.75	5.88	0.90	1.24	6.13	0.80	**0.62**	6.15	0.90	1.11	6.27	0.70	0.83	6.33	0.40	**0.52**	6.33
d05	0.37	22.26	0.90	0.65	23.80	0.20	0.46	23.66	0.90	0.68	23.54	0.30	0.55	23.40	0.10	0.58	23.43
d06	4.48	1.64	0.70	7.46	1.61	0.40	4.48	1.67	0.80	7.46	1.76	0.30	4.48	1.90	0.00	**0.00**	2.17
d07	1.94	1.62	0.70	**0.00**	1.60	0.10	**0.00**	1.61	0.60	**0.00**	1.65	0.60	**0.00**	1.87	0.20	3.88	1.89
d08	2.74	3.85	0.90	**2.71**	3.97	0.60	**1.77**	3.98	0.90	**2.52**	4.05	0.20	**2.33**	4.13	0.10	**1.96**	4.23
d09	2.59	6.54	0.90	**2.56**	6.80	0.70	**1.86**	6.77	0.90	3.04	6.83	0.60	**1.59**	6.88	0.40	**2.07**	6.91
d10	0.85	22.70	0.90	1.33	23.37	0.30	1.00	23.25	0.90	1.37	23.41	0.50	0.85	23.71	0.30	0.95	23.88
d11	27.59	3.39	0.90	**0.00**	3.38	0.90	**0.00**	3.50	0.80	**10.34**	3.36	0.10	**3.45**	3.84	0.10	**3.45**	4.05
d12	0.00	3.40	0.70	0.00	3.10	0.70	4.76	3.18	0.90	0.00	3.51	0.10	0.00	3.69	0.10	0.00	4.03
d13	5.12	5.54	0.90	**5.00**	5.69	0.90	5.20	5.76	0.90	5.20	5.80	0.30	**4.00**	6.09	0.40	**3.60**	6.27
d14	3.71	8.25	0.90	**3.00**	8.72	0.90	**2.40**	8.78	0.90	**2.02**	8.84	0.10	**2.40**	9.13	0.90	**2.25**	9.24
d15	2.20	23.88	0.90	**1.61**	25.86	0.90	**1.25**	25.79	0.90	2.59	25.44	0.20	**1.52**	25.72	0.10	**1.79**	26.33
d16	7.69	6.92	0.80	7.69	5.88	0.90	7.69	6.54	0.80	7.69	6.22	0.20	**0.00**	7.45	0.10	15.38	8.37
d17	2.87	6.93	0.90	8.70	6.50	0.90	8.70	6.60	0.90	4.35	7.04	0.30	4.35	7.69	0.10	**0.00**	8.48
d18	7.42	8.99	0.90	8.07	8.95	0.90	**7.17**	8.97	0.90	7.63	9.42	0.20	8.07	10.05	0.10	8.52	10.72
d19	7.38	11.56	0.90	8.39	11.82	0.90	8.06	11.91	0.90	**5.27**	12.34	0.40	8.39	13.02	0.10	**6.13**	13.59
d20	6.66	26.29	0.80	**4.00**	27.62	0.90	**4.47**	28.25	0.60	**4.31**	25.59	0.60	**5.96**	28.06	0.10	7.82	28.57
average	4.70	8.32	-	**3.37**	8.57	-	**3.27**	8.65	-	**3.54**	8.60	-	**2.72**	8.99	-	**3.24**	9.26
				(-1.33)			(-1.43)			(-1.16)			(-1.98)			(-1.46)	

Table S20. Results of the benchmark problem set E by using the ADH.

	without		with centrality															
	centrality			degree			eigenvector			closeness			vertex betweenness			edge betweenness	
name	gap	time	α	gap	time	α	gap	time	α	gap	time	α	gap	time	α	gap	time
e01	1.80	16.58	0.00	1.80	17.71	0.10	1.80	17.21	0.80	3.60	17.22	0.20	**0.00**	16.11	0.40	1.80	19.58
e02	9.81	16.12	0.40	**6.07**	16.87	0.40	**5.14**	13.33	0.90	**5.14**	18.27	0.60	**5.14**	19.53	0.30	**7.94**	16.96
e03	2.04	66.68	0.80	**1.97**	67.08	0.90	2.39	65.80	0.90	**1.99**	66.45	0.10	**1.92**	68.31	0.90	**1.84**	67.04
e04	1.39	126.95	0.90	1.39	126.95	0.60	**1.16**	134.01	0.80	1.57	132.60	0.30	**1.27**	132.79	0.80	**1.08**	137.04
e05	0.97	497.92	0.80	**0.92**	495.64	0.10	**0.96**	503.36	0.90	**0.87**	488.33	0.70	**0.69**	510.72	0.90	**0.81**	512.05
e06	0.00	30.62	0.80	0.00	30.37	0.30	0.00	31.92	0.90	9.59	31.66	0.10	6.85	33.71	0.10	0.00	33.88
e07	2.76	26.27	0.90	2.76	26.28	0.90	3.45	31.16	0.90	11.72	31.90	0.20	2.76	34.49	0.10	2.76	34.65
e08	2.23	83.56	0.90	2.23	83.56	0.20	2.73	84.37	0.90	2.55	84.13	0.60	**2.05**	80.05	0.70	**1.67**	87.83
e09	2.55	151.27	0.90	2.55	151.27	0.80	3.02	150.87	0.90	3.65	150.63	0.60	**2.22**	155.31	0.30	**2.30**	156.16
e10	1.11	486.66	0.90	1.11	486.66	0.80	**0.96**	486.10	0.90	**1.08**	498.64	0.80	**1.05**	500.74	0.10	1.14	507.19
e11	0.00	62.61	0.80	0.00	60.50	0.70	2.94	58.28	0.90	0.00	61.22	0.10	0.00	67.47	0.10	0.00	78.18
e12	17.91	63.29	0.70	**8.96**	58.41	0.90	**2.99**	65.43	0.90	**13.43**	62.68	0.10	**2.99**	69.93	0.80	**7.46**	73.67
e13	4.63	115.43	0.90	4.63	115.43	0.90	**3.75**	117.53	0.90	**4.14**	117.66	0.30	**3.75**	125.81	0.90	**3.44**	122.31
e14	4.34	185.72	0.90	4.34	185.72	0.90	**3.81**	184.79	0.90	**4.03**	184.62	0.10	**3.35**	194.51	0.10	**2.94**	196.28
e15	1.68	563.15	0.90	1.68	563.15	0.90	**1.58**	553.92	0.90	**1.45**	546.77	0.20	**1.08**	565.02	0.10	**0.97**	567.47
e16	20.00	118.75	0.80	**6.67**	109.10	0.80	20.00	111.35	0.80	**6.67**	115.63	0.10	**0.00**	148.53	0.10	**6.67**	166.17
e17	8.00	121.22	0.90	8.00	121.22	0.90	8.00	121.43	0.90	**0.00**	128.12	0.10	**4.00**	143.63	0.00	8.00	174.16
e18	10.77	174.07	0.90	10.77	174.07	0.90	10.99	174.15	0.90	**10.38**	178.86	0.40	11.35	205.76	0.10	**9.75**	220.15
e19	8.64	216.58	0.90	8.64	216.58	0.90	**7.52**	227.07	0.90	**7.58**	226.54	0.50	**6.60**	254.04	0.10	**7.52**	283.43
e20	5.25	666.62	0.90	5.25	666.62	0.90	5.37	597.17	0.40	**4.83**	606.35	0.60	6.04	633.01	0.10	6.48	620.48
average	5.29	189.50	-	**3.99**	188.66	-	**4.43**	186.46	-	**4.71**	187.41	-	**3.15**	197.97	-	**3.73**	203.73
				(-1.30)			(-0.86)			(-0.58)			(-2.14)			(-1.56)	

Table S21. Results of the benchmark problem set I080 by using the ADH. Texts with a gray background are complete graph instances.

	without centrality		with centrality															
			degree			eigenvector			closeness			vertex betweenness			edge betweenness		
name	gap	time	α	gap	time	α	gap	time	α	gap	time	α	gap	time	α	gap	time
i080-001	0.00	0.00	0.70	0.00	0.00	0.80	0.00	0.00	0.60	0.00	0.00	0.90	4.70	0.00	0.70	4.70	0.00
i080-002	5.23	0.00	0.00	**0.00**	0.00	0.10	5.23	0.00	0.00	**0.00**	0.00	0.00	**0.00**	0.00	0.40	**0.00**	0.00
i080-003	0.00	0.00	0.00	0.00	0.00	0.00	0.00	0.00	0.20	0.00	0.00	0.00	0.00	0.00	0.00	0.00	0.00
i080-004	5.20	0.00	0.50	6.65	0.00	0.80	5.20	0.00	0.00	6.81	0.00	0.50	5.20	0.00	0.30	5.20	0.00
i080-005	0.61	0.00	0.70	**0.22**	0.00	0.70	0.61	0.00	0.50	0.84	0.00	0.30	**0.22**	0.00	0.80	**0.22**	0.00
i080-011	2.37	0.00	0.80	**0.81**	0.00	0.90	2.91	0.00	0.10	2.50	0.00	0.00	**0.00**	0.00	0.80	**0.20**	0.00
i080-012	10.78	0.00	0.00	**0.88**	0.00	0.80	**0.47**	0.00	0.90	**0.47**	0.00	0.60	**0.34**	0.00	0.10	**0.34**	0.00
i080-013	7.97	0.00	0.00	**0.87**	0.00	0.70	7.97	0.00	0.10	**0.51**	0.00	0.10	**0.00**	0.00	0.30	**0.00**	0.00
i080-014	0.00	0.00	0.90	5.87	0.00	0.90	0.00	0.00	0.90	0.00	0.00	0.80	0.00	0.00	0.70	0.00	0.00
i080-015	0.94	0.00	0.00	7.16	0.00	0.90	0.94	0.00	0.90	6.49	0.00	0.20	**0.54**	0.00	0.20	**0.54**	0.00
i080-021	0.00	0.01	0.60	0.00	0.01	0.70	0.00	0.01	0.40	0.00	0.01	0.60	0.00	0.01	0.60	0.00	0.01
i080-022	0.08	0.01	0.60	0.08	0.01	0.70	0.08	0.01	0.30	0.08	0.01	0.60	0.08	0.01	0.60	0.08	0.01
i080-023	0.00	0.01	0.60	0.00	0.01	0.90	0.00	0.01	0.30	0.00	0.01	0.60	0.00	0.01	0.60	0.00	0.01
i080-024	0.00	0.01	0.60	0.00	0.01	0.70	0.00	0.01	0.60	0.00	0.01	0.60	0.00	0.01	0.60	0.00	0.01
i080-025	0.00	0.01	0.50	0.00	0.01	0.60	0.00	0.01	0.30	0.00	0.01	0.50	0.00	0.01	0.50	0.00	0.01
i080-031	0.00	0.00	0.40	0.00	0.00	0.10	0.00	0.00	0.30	0.00	0.00	0.50	0.00	0.00	0.80	0.00	0.00
i080-032	4.65	0.00	0.60	5.03	0.00	0.90	4.65	0.00	0.90	**4.50**	0.00	0.20	**0.05**	0.00	0.20	**0.05**	0.00
i080-033	5.18	0.00	0.60	5.18	0.00	0.30	**0.67**	0.00	0.20	**0.67**	0.00	0.70	**0.22**	0.00	0.10	**0.00**	0.00
i080-034	4.98	0.00	0.10	**0.24**	0.00	0.20	**0.24**	0.00	0.00	**0.24**	0.00	0.10	5.33	0.00	0.30	5.39	0.00
i080-035	5.16	0.00	0.60	**0.00**	0.00	0.50	**0.00**	0.00	0.00	**0.00**	0.00	0.70	**0.00**	0.00	0.90	**0.00**	0.00
i080-041	1.57	0.00	0.50	1.57	0.00	0.90	**0.24**	0.00	0.90	**0.24**	0.01	0.20	**0.24**	0.01	0.50	**0.24**	0.01
i080-042	6.29	0.00	0.80	8.08	0.00	0.60	**0.00**	0.00	0.90	6.29	0.01	0.80	**5.36**	0.01	0.50	**5.13**	0.01
i080-043	0.00	0.00	0.90	6.18	0.00	0.40	0.15	0.00	0.70	6.25	0.01	0.70	6.25	0.01	0.70	0.15	0.01
i080-044	0.00	0.00	0.90	1.10	0.00	0.90	1.02	0.00	0.90	0.00	0.01	0.70	0.37	0.01	0.60	0.37	0.01
i080-045	5.50	0.00	0.40	**0.08**	0.00	0.60	**0.08**	0.00	0.10	**0.08**	0.00	0.40	**0.08**	0.01	0.90	**0.08**	0.01
i080-101	10.66	0.00	0.90	**3.83**	0.00	0.30	**3.68**	0.00	0.60	**7.17**	0.00	0.10	**3.91**	0.00	0.60	**3.83**	0.00
i080-102	0.08	0.00	0.50	0.21	0.00	0.10	0.08	0.00	0.90	0.08	0.00	0.80	0.21	0.00	0.70	4.41	0.00
i080-103	2.46	0.00	0.60	2.46	0.00	0.90	2.46	0.00	0.90	2.46	0.00	0.10	2.46	0.00	0.20	2.46	0.00
i080-104	0.00	0.00	0.30	3.86	0.00	0.10	0.00	0.00	0.20	0.00	0.00	0.90	0.00	0.00	0.40	0.00	0.00
i080-105	8.85	0.00	0.00	**0.00**	0.00	0.00	**0.00**	0.00	0.50	**4.09**	0.00	0.20	**4.09**	0.00	0.10	**8.31**	0.00
i080-111	0.24	0.00	0.90	**0.00**	0.00	0.80	0.44	0.00	0.90	5.02	0.00	0.10	**0.15**	0.00	0.90	0.24	0.00
i080-112	4.30	0.00	0.00	**0.74**	0.00	0.20	**0.74**	0.00	0.20	**0.16**	0.00	0.80	**3.66**	0.00	0.50	5.15	0.00
i080-113	10.67	0.00	0.90	**5.73**	0.00	0.50	**6.05**	0.00	0.70	**5.73**	0.00	0.90	**5.31**	0.00	0.20	**0.37**	0.00
i080-114	10.87	0.00	0.80	**4.96**	0.00	0.90	**0.00**	0.00	0.90	**9.97**	0.00	0.10	**0.00**	0.00	0.60	**0.00**	0.00
i080-115	0.11	0.00	0.90	5.14	0.00	0.90	0.11	0.00	0.70	0.11	0.00	0.60	0.16	0.00	0.40	0.16	0.00
i080-121	0.32	0.01	0.40	0.32	0.01	0.60	0.32	0.01	0.60	0.32	0.01	0.40	0.32	0.01	0.40	0.32	0.01
i080-122	0.00	0.01	0.50	0.00	0.01	0.60	0.00	0.01	0.30	0.00	0.01	0.50	0.00	0.01	0.50	0.00	0.01
i080-123	0.00	0.01	0.40	0.00	0.01	0.60	0.00	0.01	0.00	0.00	0.01	0.40	0.00	0.01	0.40	0.00	0.01
i080-124	0.00	0.01	0.50	0.00	0.01	0.60	0.00	0.01	0.10	0.00	0.01	0.50	0.00	0.01	0.50	0.00	0.01
i080-125	0.00	0.01	0.40	0.00	0.01	0.60	0.00	0.01	0.40	0.00	0.01	0.40	0.00	0.01	0.40	0.00	0.01
i080-131	8.36	0.00	0.40	**3.81**	0.00	0.10	**3.81**	0.00	0.40	**3.81**	0.00	0.60	**4.12**	0.00	0.50	**4.33**	0.00
i080-132	0.00	0.00	0.80	5.28	0.00	0.80	3.94	0.00	0.10	5.28	0.00	0.90	0.00	0.00	0.20	0.32	0.00
i080-133	4.16	0.00	0.10	**0.84**	0.00	0.70	**0.09**	0.00	0.00	**0.93**	0.00	0.60	**0.00**	0.00	0.50	**0.88**	0.00
i080-134	0.97	0.00	0.40	1.40	0.00	0.70	0.97	0.00	0.10	1.40	0.00	0.10	**0.43**	0.00	0.30	**0.43**	0.00
i080-135	3.66	0.00	0.70	**0.00**	0.00	0.10	**0.00**	0.00	0.80	**0.00**	0.00	0.40	**0.19**	0.00	0.90	**0.33**	0.00
i080-141	4.87	0.00	0.80	**0.00**	0.00	0.60	**0.00**	0.00	0.40	**0.00**	0.01	0.50	**0.00**	0.01	0.40	**0.00**	0.01
i080-142	7.85	0.00	0.60	**0.00**	0.00	0.50	**0.00**	0.00	0.30	**0.00**	0.01	0.90	**4.80**	0.01	0.50	8.43	0.01
i080-143	0.85	0.00	0.60	**0.57**	0.00	0.20	**0.74**	0.00	0.40	**0.74**	0.01	0.70	5.43	0.01	0.20	**0.06**	0.01
i080-144	5.36	0.00	0.90	5.36	0.00	0.90	6.26	0.00	0.90	5.36	0.01	0.70	5.36	0.01	0.90	5.36	0.01
i080-145	0.00	0.00	0.70	0.51	0.00	0.90	0.51	0.00	0.90	0.51	0.01	0.30	0.40	0.01	0.90	0.00	0.01

Table S21. (Continued) Results of the benchmark problem set I080 by using the ADH. Texts with a gray background are complete graph instances.

	without centrality		with centrality															
			degree			eigenvector			closeness			vertex betweenness			edge betweenness			
name	gap	time	α	gap	time	α	gap	time	α	gap	time	α	gap	time	α	gap	time
i080-201	4.03	0.00	0.60	**0.00**	0.00	0.50	**0.00**	0.00	0.60	**0.00**	0.00	0.10	**0.00**	0.00	0.40	**0.00**	0.00
i080-202	0.00	0.00	0.20	2.82	0.00	0.90	0.00	0.00	0.70	4.62	0.00	0.80	0.17	0.00	0.90	0.24	0.00
i080-203	4.13	0.00	0.80	**4.09**	0.00	0.20	**2.41**	0.00	0.00	**2.41**	0.00	0.10	**3.96**	0.00	0.10	**0.33**	0.00
i080-204	3.98	0.00	0.80	4.14	0.00	0.40	**2.03**	0.00	0.00	**3.96**	0.00	0.10	**3.58**	0.00	0.20	**2.03**	0.00
i080-205	4.16	0.00	0.90	**2.26**	0.00	0.80	**2.19**	0.00	0.20	**0.53**	0.00	0.90	**2.19**	0.00	0.10	**2.39**	0.00
i080-211	2.59	0.00	0.80	4.19	0.00	0.90	2.62	0.00	0.50	4.05	0.00	0.30	**0.44**	0.01	0.90	2.59	0.01
i080-212	5.28	0.00	0.90	**0.41**	0.00	0.70	**0.30**	0.00	0.80	**0.41**	0.01	0.40	**0.41**	0.01	0.80	**0.41**	0.01
i080-213	5.19	0.00	0.80	**2.77**	0.00	0.80	**4.54**	0.00	0.70	**2.34**	0.01	0.20	**2.26**	0.01	0.20	**1.50**	0.01
i080-214	3.40	0.00	0.70	3.83	0.00	0.70	3.40	0.00	0.60	**0.67**	0.01	0.10	**0.72**	0.01	0.20	**0.51**	0.01
i080-215	1.96	0.00	0.90	1.96	0.00	0.20	3.37	0.00	0.80	3.18	0.01	0.50	1.96	0.01	0.80	1.96	0.01
i080-221	0.00	0.01	0.30	0.00	0.01	0.60	0.00	0.01	0.70	0.00	0.01	0.30	0.00	0.01	0.30	0.00	0.01
i080-222	0.00	0.01	0.30	0.00	0.01	0.70	0.00	0.01	0.10	0.00	0.01	0.30	0.00	0.01	0.30	0.00	0.01
i080-223	0.06	0.01	0.30	0.06	0.01	0.60	0.06	0.01	0.70	0.06	0.01	0.30	0.06	0.01	0.30	0.06	0.01
i080-224	0.06	0.01	0.30	0.06	0.01	0.50	0.06	0.01	0.50	**0.00**	0.01	0.30	0.06	0.01	0.30	0.06	0.01
i080-225	0.00	0.01	0.30	0.00	0.01	0.50	0.00	0.01	0.10	0.00	0.01	0.30	0.00	0.01	0.30	0.00	0.01
i080-231	6.68	0.00	0.80	**0.00**	0.00	0.60	**2.41**	0.00	0.90	**0.00**	0.00	0.70	**2.25**	0.00	0.60	**2.41**	0.00
i080-232	6.50	0.00	0.50	**2.50**	0.00	0.80	**4.41**	0.00	0.00	**2.50**	0.00	0.90	6.50	0.00	0.90	**2.07**	0.00
i080-233	5.05	0.00	0.90	**0.44**	0.00	0.30	**4.64**	0.00	0.70	**0.83**	0.00	0.50	**4.91**	0.00	0.40	**0.78**	0.00
i080-234	6.46	0.00	0.60	**2.50**	0.00	0.10	**4.61**	0.00	0.70	**4.12**	0.00	0.80	**0.05**	0.00	0.50	**2.18**	0.00
i080-235	7.13	0.00	0.40	**1.83**	0.00	0.10	**1.83**	0.00	0.20	**1.83**	0.00	0.30	**2.09**	0.00	0.30	**0.00**	0.00
i080-241	3.17	0.00	0.70	**0.65**	0.00	0.90	**1.38**	0.00	0.50	**0.65**	0.01	0.70	**0.00**	0.01	0.70	**0.00**	0.01
i080-242	2.83	0.00	0.80	**0.32**	0.00	0.80	**0.81**	0.00	0.50	**0.46**	0.01	0.40	**0.12**	0.01	0.90	**1.68**	0.01
i080-243	4.20	0.00	0.90	**0.00**	0.00	0.80	**0.12**	0.00	0.50	**2.96**	0.01	0.70	**1.87**	0.01	0.40	**0.12**	0.01
i080-244	3.06	0.00	0.90	**2.45**	0.00	0.50	3.09	0.00	0.50	3.09	0.01	0.70	3.06	0.01	0.40	3.87	0.01
i080-245	2.77	0.00	0.90	3.89	0.00	0.60	3.40	0.00	0.60	3.40	0.01	0.50	**1.90**	0.01	0.60	**1.90**	0.01
i080-301	0.20	0.00	0.80	0.27	0.00	0.80	0.20	0.00	0.50	0.27	0.00	0.80	2.21	0.00	0.50	1.83	0.00
i080-302	3.47	0.00	0.60	**3.31**	0.00	0.90	3.47	0.00	0.60	**1.67**	0.00	0.80	**1.60**	0.00	0.90	**1.60**	0.00
i080-303	7.81	0.00	0.80	**3.27**	0.00	0.20	**3.58**	0.00	0.70	**1.45**	0.00	0.20	**1.54**	0.00	0.20	**1.64**	0.00
i080-304	1.90	0.00	0.90	5.75	0.00	0.90	5.53	0.00	0.60	5.46	0.00	0.80	1.90	0.00	0.50	**1.83**	0.00
i080-305	3.41	0.00	0.40	**2.21**	0.00	0.30	**2.17**	0.00	0.70	**2.17**	0.00	0.40	**3.35**	0.00	0.20	**1.25**	0.00
i080-311	6.70	0.00	0.60	**3.58**	0.00	0.70	**3.47**	0.00	0.60	**2.94**	0.01	0.60	**3.21**	0.01	0.90	**3.21**	0.01
i080-312	0.64	0.00	0.90	0.82	0.00	0.90	0.86	0.01	0.80	0.82	0.01	0.90	**0.55**	0.01	0.70	**0.62**	0.01
i080-313	2.55	0.00	0.90	2.95	0.00	0.90	2.95	0.00	0.50	2.95	0.01	0.10	2.77	0.01	0.50	**1.80**	0.01
i080-314	0.86	0.00	0.80	2.95	0.00	0.30	1.24	0.00	0.80	0.95	0.01	0.10	2.72	0.01	0.90	0.86	0.01
i080-315	0.00	0.00	0.90	0.90	0.00	0.90	0.85	0.00	0.50	0.36	0.01	0.90	0.00	0.01	0.90	0.02	0.01
i080-321	0.00	0.01	0.20	0.00	0.01	0.50	0.00	0.01	0.10	0.00	0.01	0.20	0.00	0.01	0.20	0.00	0.01
i080-322	0.00	0.01	0.20	0.00	0.01	0.50	0.00	0.01	0.90	0.00	0.01	0.20	0.00	0.01	0.20	0.00	0.01
i080-323	0.00	0.01	0.20	0.00	0.01	0.50	0.00	0.01	0.70	0.00	0.01	0.20	0.00	0.01	0.20	0.00	0.01
i080-324	0.00	0.01	0.20	0.00	0.01	0.50	0.00	0.01	0.00	0.00	0.01	0.20	0.00	0.01	0.20	0.00	0.01
i080-325	0.00	0.01	0.20	0.00	0.01	0.50	0.00	0.01	0.50	0.00	0.01	0.20	0.00	0.01	0.20	0.00	0.01
i080-331	4.78	0.00	0.70	**1.63**	0.00	0.90	**1.17**	0.00	0.50	**1.63**	0.00	0.90	**1.78**	0.00	0.70	**1.93**	0.00
i080-332	2.29	0.00	0.20	**0.73**	0.00	0.50	**0.13**	0.00	0.70	**0.34**	0.00	0.80	4.03	0.00	0.30	**1.75**	0.00
i080-333	4.16	0.00	0.40	**1.77**	0.00	0.80	**1.12**	0.00	0.50	**3.57**	0.00	0.40	**3.61**	0.00	0.60	**2.86**	0.00
i080-334	3.13	0.00	0.90	**1.92**	0.00	0.90	**0.06**	0.00	0.70	**2.18**	0.00	0.80	**1.67**	0.00	0.90	**1.69**	0.00
i080-335	5.09	0.00	0.60	**0.59**	0.00	0.70	5.33	0.00	0.70	**1.86**	0.00	0.10	**0.00**	0.00	0.20	**0.10**	0.00
i080-341	0.00	0.00	0.90	2.38	0.00	0.70	0.05	0.01	0.90	0.00	0.01	0.90	0.00	0.01	0.50	1.63	0.01
i080-342	0.16	0.00	0.80	0.25	0.00	0.90	2.47	0.01	0.70	0.48	0.01	0.80	0.16	0.01	0.90	0.21	0.01
i080-343	3.72	0.00	0.90	**2.52**	0.01	0.70	**2.52**	0.01	0.60	**2.52**	0.01	0.70	**1.46**	0.01	0.50	**1.30**	0.01
i080-344	2.02	0.00	0.90	**0.14**	0.01	0.80	2.20	0.01	0.80	**0.00**	0.01	0.80	2.02	0.01	0.80	2.02	0.01
i080-345	0.23	0.00	0.90	1.75	0.00	0.80	2.28	0.01	0.70	2.42	0.01	0.20	0.39	0.01	0.80	1.75	0.01
average	2.87	0.00	-	**1.76** (-1.11)	0.00	-	**1.50** (-1.37)	0.00	-	**1.65** (-1.22)	0.01	-	**1.44** (-1.43)	0.01	-	**1.25** (-1.62)	0.01

Table S22. Results of the benchmark problem set I160 by using the ADH. Texts with a gray background are complete graph instances.

	without centrality		with centrality															
			degree			eigenvector			closeness			vertex betweenness			edge betweenness			
name	gap	time	α	gap	time	α	gap	time	α	gap	time	α	gap	time	α	gap	time
i160-001	10.76	0.01	0.60	**4.38**	0.01	0.10	**7.83**	0.01	0.50	**0.84**	0.01	0.10	**0.44**	0.01	0.70	**0.00**	0.01
i160-002	2.14	0.01	0.90	**0.00**	0.01	0.60	**0.09**	0.01	0.00	**0.93**	0.01	0.60	**0.00**	0.01	0.70	**0.09**	0.01
i160-003	4.14	0.01	0.90	**0.04**	0.01	0.30	4.31	0.01	0.80	**0.04**	0.01	0.80	**0.00**	0.01	0.50	**0.00**	0.01
i160-004	9.20	0.01	0.30	**0.00**	0.01	0.00	**5.27**	0.01	0.20	**5.27**	0.01	0.70	**5.27**	0.01	0.10	9.20	0.01
i160-005	0.00	0.01	0.90	0.00	0.01	0.70	0.20	0.01	0.60	4.01	0.01	0.30	0.20	0.01	0.90	0.20	0.01
i160-011	5.31	0.01	0.90	5.43	0.02	0.70	**0.54**	0.02	0.90	**5.07**	0.02	0.10	5.31	0.02	0.30	**0.18**	0.02
i160-012	6.91	0.01	0.70	**0.00**	0.02	0.70	**0.00**	0.02	0.80	**0.00**	0.02	0.20	**6.23**	0.02	0.30	**0.00**	0.02
i160-013	11.50	0.01	0.80	**5.48**	0.02	0.10	**6.50**	0.02	0.00	**7.22**	0.02	0.30	**7.04**	0.02	0.20	**6.14**	0.02
i160-014	6.02	0.01	0.00	**1.57**	0.01	0.50	6.41	0.02	0.10	6.41	0.02	0.60	6.02	0.02	0.90	**0.51**	0.02
i160-015	11.09	0.01	0.90	**0.00**	0.02	0.80	11.99	0.02	0.80	**0.00**	0.02	0.70	**4.69**	0.02	0.70	**4.69**	0.02
i160-021	0.00	0.04	0.50	0.00	0.04	0.60	0.00	0.04	0.10	0.00	0.07	0.50	0.00	0.07	0.50	0.00	0.07
i160-022	0.00	0.04	0.50	0.00	0.04	0.60	0.00	0.04	0.10	0.00	0.07	0.50	0.00	0.07	0.50	0.00	0.07
i160-023	0.00	0.04	0.50	0.00	0.04	0.60	0.00	0.04	0.10	0.00	0.07	0.50	0.00	0.07	0.50	0.00	0.07
i160-024	0.36	0.04	0.50	0.36	0.04	0.60	0.36	0.04	0.70	0.36	0.07	0.50	0.36	0.07	0.50	0.36	0.07
i160-025	1.83	0.04	0.60	**0.00**	0.04	0.60	**0.00**	0.04	0.10	1.83	0.07	0.60	**0.00**	0.07	0.60	**0.00**	0.07
i160-031	0.00	0.01	0.30	0.00	0.01	0.50	0.00	0.01	0.00	0.00	0.01	0.50	0.00	0.01	0.80	4.79	0.01
i160-032	10.69	0.01	0.90	**2.23**	0.01	0.30	**6.44**	0.01	0.10	**6.65**	0.01	0.50	**0.00**	0.01	0.40	**0.00**	0.01
i160-033	13.61	0.01	0.60	**0.33**	0.01	0.20	**3.95**	0.01	0.60	**0.00**	0.01	0.80	**0.00**	0.01	0.80	**3.76**	0.01
i160-034	0.00	0.01	0.60	0.00	0.01	0.50	4.32	0.01	0.00	0.00	0.01	0.60	6.43	0.01	0.90	0.00	0.01
i160-035	5.14	0.01	0.90	9.13	0.01	0.30	**1.09**	0.01	0.80	9.13	0.01	0.80	7.56	0.01	0.50	**0.00**	0.01
i160-041	0.00	0.02	0.80	0.00	0.02	0.80	0.00	0.02	0.60	0.00	0.03	0.20	0.00	0.03	0.80	3.55	0.04
i160-042	6.33	0.02	0.70	**0.87**	0.02	0.70	**0.87**	0.02	0.20	**5.79**	0.03	0.10	9.76	0.03	0.40	9.35	0.04
i160-043	0.00	0.02	0.60	1.48	0.02	0.70	1.36	0.02	0.10	1.03	0.03	0.50	0.00	0.03	0.30	0.00	0.04
i160-044	11.57	0.02	0.70	**0.00**	0.02	0.70	**6.09**	0.02	0.30	**0.00**	0.03	0.10	**0.00**	0.03	0.50	**5.41**	0.04
i160-045	0.39	0.02	0.90	6.44	0.02	0.90	0.45	0.03	0.50	0.39	0.03	0.50	0.39	0.03	0.90	0.39	0.04
i160-101	0.00	0.01	0.80	0.00	0.01	0.50	0.00	0.01	0.90	0.00	0.01	0.10	0.00	0.01	0.20	0.00	0.01
i160-102	7.63	0.01	0.90	**2.24**	0.01	0.10	**4.91**	0.01	0.40	**3.15**	0.01	0.60	**0.00**	0.01	0.30	**0.00**	0.01
i160-103	2.06	0.01	0.00	**0.29**	0.01	0.10	**0.31**	0.01	0.80	**0.31**	0.01	0.10	**0.42**	0.01	0.10	**0.29**	0.01
i160-104	14.10	0.01	0.40	**0.05**	0.01	0.00	**2.71**	0.01	0.20	**0.05**	0.01	0.40	**9.60**	0.01	0.10	**12.06**	0.01
i160-105	7.91	0.01	0.50	**0.00**	0.01	0.10	**0.00**	0.01	0.90	**2.58**	0.01	0.20	**0.00**	0.01	0.70	**2.58**	0.01
i160-111	7.11	0.02	0.40	**4.53**	0.02	0.80	**4.36**	0.02	0.90	**4.36**	0.02	0.20	**3.31**	0.02	0.30	**3.07**	0.02
i160-112	0.48	0.02	0.80	**0.07**	0.02	0.70	**0.07**	0.02	0.70	**0.00**	0.02	0.80	0.48	0.02	0.60	**0.03**	0.02
i160-113	6.07	0.02	0.60	**3.21**	0.02	0.20	**0.00**	0.02	0.90	**3.11**	0.02	0.50	6.07	0.02	0.70	**0.73**	0.02
i160-114	4.18	0.02	0.80	**0.07**	0.02	0.90	**2.14**	0.02	0.90	**1.71**	0.02	0.60	**1.71**	0.02	0.30	**1.77**	0.02
i160-115	9.19	0.02	0.90	**4.09**	0.02	0.70	**0.54**	0.02	0.80	**4.32**	0.02	0.30	**1.36**	0.02	0.30	**0.99**	0.02
i160-121	0.04	0.04	0.30	0.04	0.04	0.50	0.04	0.04	0.10	0.04	0.07	0.30	0.04	0.07	0.30	0.04	0.07
i160-122	0.00	0.04	0.30	0.00	0.04	0.50	0.00	0.04	0.10	0.00	0.07	0.30	0.00	0.07	0.30	0.00	0.07
i160-123	0.00	0.04	0.30	0.00	0.04	0.50	0.00	0.04	0.10	0.00	0.07	0.30	0.00	0.07	0.30	0.00	0.07
i160-124	0.26	0.04	0.30	**0.00**	0.04	0.50	0.26	0.04	0.10	0.26	0.07	0.30	**0.00**	0.07	0.30	**0.00**	0.07
i160-125	0.00	0.04	0.30	0.00	0.04	0.50	0.00	0.04	0.10	0.00	0.07	0.30	0.00	0.07	0.30	0.00	0.07
i160-131	5.10	0.01	0.60	**0.60**	0.01	0.70	**2.65**	0.01	0.90	**0.48**	0.01	0.80	**0.77**	0.01	0.60	**2.53**	0.01
i160-132	2.38	0.01	0.30	3.71	0.01	0.80	**0.87**	0.01	0.70	**1.36**	0.01	0.90	2.61	0.01	0.90	**2.32**	0.01
i160-133	15.40	0.01	0.50	**10.99**	0.01	0.80	**12.86**	0.01	0.00	**5.66**	0.01	0.30	**2.57**	0.02	0.10	**7.95**	0.02
i160-134	8.18	0.01	0.00	**0.55**	0.01	0.90	**5.82**	0.01	0.00	**0.55**	0.01	0.80	**3.26**	0.02	0.30	**3.03**	0.02
i160-135	6.14	0.01	0.30	**5.44**	0.01	0.30	**3.28**	0.01	0.90	**3.42**	0.01	0.30	**0.86**	0.01	0.30	**0.81**	0.01
i160-141	3.45	0.02	0.70	**0.00**	0.02	0.60	**0.00**	0.02	0.60	**0.00**	0.03	0.50	**0.00**	0.03	0.90	**0.24**	0.04
i160-142	1.37	0.02	0.90	5.97	0.02	0.80	2.22	0.02	0.50	1.91	0.03	0.30	1.37	0.03	0.30	1.37	0.04
i160-143	6.61	0.02	0.60	**3.72**	0.02	0.70	**3.72**	0.02	0.60	**3.60**	0.03	0.10	**4.38**	0.03	0.80	**5.94**	0.04
i160-144	4.76	0.02	0.90	**0.50**	0.02	0.60	**1.27**	0.02	0.30	**2.95**	0.03	0.60	**0.19**	0.04	0.70	**0.19**	0.04
i160-145	1.12	0.02	0.90	1.12	0.03	0.70	1.12	0.02	0.50	1.12	0.03	0.20	**0.16**	0.03	0.60	2.25	0.04

Table S22. (Continued) Results of the benchmark problem set I160 by using the ADH. Texts with a gray background are complete graph instances.

	without centrality		with centrality															
			degree			eigenvector			closeness			vertex betweenness			edge betweenness			
name	gap	time	α	gap	time	α	gap	time	α	gap	time	α	gap	time	α	gap	time
i160-201	0.78	0.01	0.20	4.52	0.01	0.50	0.78	0.01	0.40	1.81	0.02	0.60	0.78	0.02	0.50	0.78	0.02
i160-202	1.33	0.01	0.90	1.53	0.01	0.90	1.33	0.01	0.90	**0.20**	0.02	0.90	2.84	0.02	0.80	1.33	0.02
i160-203	10.56	0.01	0.90	**7.99**	0.01	0.10	**6.49**	0.01	0.70	**7.94**	0.02	0.30	**4.67**	0.02	0.60	**5.44**	0.02
i160-204	2.62	0.01	0.50	2.94	0.01	0.90	2.62	0.01	0.90	4.20	0.02	0.90	**1.23**	0.02	0.90	**1.25**	0.02
i160-205	2.64	0.01	0.50	3.06	0.01	0.40	2.64	0.01	0.90	2.82	0.02	0.80	2.64	0.02	0.80	**1.39**	0.02
i160-211	4.67	0.02	0.90	**2.27**	0.02	0.80	5.02	0.02	0.80	4.67	0.03	0.40	**2.06**	0.03	0.50	**2.06**	0.03
i160-212	3.23	0.02	0.70	3.54	0.02	0.80	**0.58**	0.02	0.80	3.77	0.03	0.40	3.30	0.03	0.50	4.98	0.03
i160-213	4.04	0.02	0.80	**2.71**	0.02	0.80	**2.53**	0.02	0.70	**2.62**	0.03	0.40	4.13	0.03	0.20	**2.16**	0.03
i160-214	3.23	0.02	0.70	3.74	0.02	0.70	4.07	0.02	0.80	**3.06**	0.03	0.10	**2.47**	0.03	0.70	3.23	0.03
i160-215	6.51	0.02	0.80	**5.73**	0.02	0.70	**5.74**	0.02	0.90	6.92	0.03	0.20	**5.13**	0.03	0.50	**5.09**	0.03
i160-221	0.00	0.04	0.20	0.00	0.04	0.50	0.00	0.04	0.10	0.00	0.07	0.20	0.00	0.07	0.20	0.00	0.08
i160-222	0.00	0.04	0.20	0.00	0.04	0.50	0.00	0.04	0.10	0.00	0.07	0.20	0.00	0.07	0.20	0.00	0.08
i160-223	0.00	0.04	0.20	0.00	0.04	0.50	0.00	0.04	0.10	0.00	0.07	0.20	0.00	0.07	0.20	0.00	0.08
i160-224	0.00	0.04	0.20	0.00	0.04	0.50	0.00	0.04	0.10	0.00	0.07	0.20	0.00	0.07	0.20	0.00	0.08
i160-225	0.00	0.04	0.20	0.00	0.04	0.50	0.00	0.04	0.10	0.00	0.07	0.20	0.00	0.07	0.20	0.00	0.08
i160-231	12.73	0.01	0.20	**2.48**	0.01	0.10	**5.79**	0.02	0.90	**6.02**	0.02	0.40	**8.18**	0.02	0.80	**3.90**	0.02
i160-232	6.77	0.01	0.80	**5.76**	0.01	0.40	**2.90**	0.02	0.70	7.41	0.02	0.20	**3.23**	0.02	0.20	**1.77**	0.02
i160-233	5.66	0.01	0.60	**0.33**	0.01	0.60	**4.15**	0.02	0.60	**3.14**	0.02	0.80	**2.54**	0.02	0.60	**2.67**	0.02
i160-234	2.43	0.01	0.00	**0.29**	0.01	0.20	**0.29**	0.02	0.50	**0.14**	0.02	0.70	**1.06**	0.02	0.70	**1.06**	0.02
i160-235	8.55	0.01	0.90	**3.25**	0.01	0.60	**1.94**	0.02	0.90	**3.16**	0.02	0.30	**3.39**	0.02	0.10	**6.09**	0.02
i160-241	0.61	0.03	0.90	1.10	0.03	0.70	1.08	0.03	0.10	1.08	0.03	0.70	**0.28**	0.04	0.70	**0.14**	0.04
i160-242	1.04	0.03	0.90	2.76	0.03	0.80	2.80	0.03	0.50	1.35	0.04	0.90	1.19	0.04	0.70	**1.00**	0.04
i160-243	2.95	0.03	0.90	**2.12**	0.03	0.70	**0.12**	0.03	0.00	2.97	0.03	0.50	**1.23**	0.04	0.90	**1.31**	0.04
i160-244	2.34	0.03	0.80	**1.36**	0.03	0.90	**1.26**	0.03	0.80	**1.28**	0.04	0.40	**1.14**	0.04	0.90	**0.57**	0.04
i160-245	2.50	0.03	0.80	2.54	0.03	0.70	**2.46**	0.03	0.90	2.50	0.04	0.60	**2.18**	0.04	0.60	**1.30**	0.04
i160-301	4.80	0.02	0.90	**4.11**	0.02	0.80	5.67	0.02	0.90	**1.68**	0.03	0.30	**1.78**	0.03	0.80	**1.67**	0.03
i160-302	3.49	0.02	0.90	**1.92**	0.02	0.70	**1.88**	0.02	0.80	**2.85**	0.03	0.40	**1.01**	0.03	0.40	3.67	0.03
i160-303	1.66	0.02	0.90	**1.06**	0.02	0.60	**1.17**	0.02	0.80	**1.17**	0.03	0.10	**0.18**	0.03	0.20	**0.29**	0.03
i160-304	0.99	0.02	0.90	1.01	0.02	0.90	**0.13**	0.02	0.90	1.08	0.03	0.60	**0.11**	0.03	0.60	**0.19**	0.03
i160-305	1.92	0.02	0.90	2.17	0.02	0.80	2.07	0.02	0.10	3.13	0.03	0.90	2.12	0.03	0.90	1.99	0.03
i160-311	4.60	0.03	0.90	**3.19**	0.03	0.90	**1.53**	0.03	0.90	**2.27**	0.04	0.40	**3.82**	0.04	0.20	**2.32**	0.04
i160-312	3.00	0.03	0.60	**2.36**	0.03	0.70	4.93	0.03	0.70	3.54	0.04	0.60	3.00	0.04	0.90	3.00	0.04
i160-313	2.13	0.03	0.90	2.58	0.03	0.80	2.69	0.03	0.90	2.51	0.04	0.60	**1.31**	0.04	0.90	**1.31**	0.04
i160-314	4.18	0.03	0.70	**2.76**	0.03	0.80	**2.34**	0.03	0.70	**2.81**	0.04	0.10	**3.24**	0.04	0.60	**2.65**	0.04
i160-315	3.29	0.03	0.90	**0.87**	0.03	0.90	**0.62**	0.03	0.90	**0.90**	0.04	0.40	**0.53**	0.04	0.50	**0.78**	0.04
i160-321	0.44	0.05	0.10	**0.34**	0.05	0.50	**0.34**	0.05	0.10	**0.34**	0.08	0.10	**0.34**	0.08	0.10	**0.34**	0.09
i160-322	0.66	0.05	0.50	**0.42**	0.05	0.50	**0.42**	0.05	0.40	**0.42**	0.09	0.50	**0.42**	0.08	0.50	**0.42**	0.09
i160-323	0.09	0.05	0.10	0.09	0.05	0.40	0.09	0.05	0.10	0.09	0.08	0.10	0.09	0.08	0.10	0.09	0.09
i160-324	0.36	0.05	0.10	0.36	0.05	0.40	0.36	0.05	0.10	0.36	0.09	0.10	0.36	0.08	0.10	0.36	0.09
i160-325	0.11	0.05	0.10	0.19	0.05	0.40	0.19	0.05	0.10	0.19	0.08	0.10	0.19	0.08	0.10	0.19	0.09
i160-331	5.74	0.02	0.70	**3.12**	0.02	0.50	**0.74**	0.03	0.90	**4.86**	0.03	0.90	**2.54**	0.03	0.50	**2.73**	0.03
i160-332	2.88	0.02	0.80	**2.71**	0.02	0.50	4.99	0.03	0.90	2.95	0.03	0.50	2.90	0.03	0.20	**1.19**	0.03
i160-333	3.59	0.03	0.90	**2.04**	0.02	0.90	**2.88**	0.03	0.90	**2.03**	0.03	0.20	**0.78**	0.03	0.50	**0.75**	0.03
i160-334	5.40	0.02	0.80	**3.02**	0.02	0.60	**2.92**	0.03	0.70	**3.71**	0.03	0.50	**3.77**	0.03	0.20	**4.88**	0.03
i160-335	8.51	0.02	0.40	**1.49**	0.02	0.30	**3.80**	0.03	0.70	**0.44**	0.03	0.50	**1.28**	0.03	0.20	**2.34**	0.03
i160-341	3.16	0.04	0.70	**1.37**	0.04	0.70	**2.62**	0.04	0.50	**1.79**	0.05	0.40	**1.08**	0.05	0.90	**1.08**	0.05
i160-342	0.96	0.04	0.80	**0.32**	0.04	0.90	**0.31**	0.04	0.80	1.11	0.05	0.60	**0.81**	0.05	0.80	**0.36**	0.05
i160-343	2.14	0.04	0.90	**1.18**	0.04	0.70	**1.60**	0.04	0.70	**1.20**	0.05	0.90	2.15	0.05	0.80	**0.83**	0.05
i160-344	0.34	0.04	0.80	0.63	0.04	0.90	0.63	0.04	0.90	0.34	0.05	0.90	0.34	0.05	0.90	0.36	0.05
i160-345	1.14	0.04	0.70	1.44	0.04	0.80	1.65	0.04	0.50	1.20	0.05	0.80	**0.98**	0.05	0.50	1.35	0.05
average	3.81	0.02	-	**1.86**	0.02	-	**2.18**	0.02	-	**2.06**	0.03	-	**1.88**	0.03	-	**1.79**	0.04
				(-1.95)			(-1.63)			(-1.75)			(-1.93)			(-2.02)	

Table S23. Results of the benchmark problem set I320 by using the ADH. Texts with a gray background are complete graph instances.

	without centrality		with centrality															
			degree			eigenvector			closeness			vertex betweenness			edge betweenness		
name	gap	time	α	gap	time	α	gap	time	α	gap	time	α	gap	time	α	gap	time
i320-001	0.00	0.04	0.80	2.84	0.05	0.70	0.00	0.05	0.90	2.84	0.06	0.20	0.00	0.06	0.70	0.00	0.06
i320-002	0.91	0.04	0.40	**0.53**	0.05	0.30	0.91	0.05	0.60	0.91	0.06	0.30	**0.00**	0.06	0.70	0.91	0.06
i320-003	3.47	0.04	0.90	**3.33**	0.05	0.10	3.47	0.05	0.90	3.47	0.06	0.40	**3.23**	0.06	0.80	**0.07**	0.06
i320-004	14.52	0.04	0.80	**6.37**	0.05	0.20	**2.99**	0.05	0.10	**4.17**	0.06	0.40	**3.48**	0.06	0.50	**2.99**	0.06
i320-005	3.41	0.04	0.90	**0.00**	0.05	0.10	**0.00**	0.05	0.10	**0.10**	0.06	0.70	**0.00**	0.06	0.90	**0.00**	0.06
i320-011	1.61	0.10	0.90	**0.68**	0.10	0.80	**1.07**	0.10	0.60	**1.02**	0.12	0.40	**0.44**	0.12	0.50	**0.44**	0.12
i320-012	4.06	0.10	0.80	4.16	0.10	0.90	4.16	0.10	0.90	12.67	0.12	0.90	4.06	0.12	0.70	4.06	0.12
i320-013	6.03	0.10	0.00	7.67	0.09	0.90	**5.94**	0.10	0.40	**2.36**	0.11	0.30	**1.30**	0.12	0.70	**1.30**	0.12
i320-014	8.98	0.10	0.90	**0.44**	0.10	0.60	**1.46**	0.09	0.90	**0.87**	0.12	0.40	**4.71**	0.12	0.20	**5.05**	0.12
i320-015	10.05	0.10	0.80	**5.54**	0.10	0.90	**7.58**	0.10	0.20	**0.97**	0.11	0.60	**0.00**	0.12	0.70	**0.00**	0.12
i320-021	0.00	0.27	0.50	0.00	0.26	0.60	0.00	0.26	0.10	0.00	0.53	0.50	0.00	0.51	0.50	0.00	0.53
i320-022	0.00	0.27	0.40	0.00	0.25	0.60	0.00	0.26	0.10	0.00	0.52	0.40	0.00	0.51	0.40	0.00	0.53
i320-023	0.00	0.27	0.40	0.00	0.25	0.60	0.00	0.26	0.10	0.00	0.52	0.40	0.00	0.51	0.40	0.00	0.53
i320-024	0.00	0.27	0.50	0.00	0.25	0.60	0.00	0.26	0.10	0.00	0.52	0.50	0.00	0.51	0.50	0.00	0.53
i320-025	0.00	0.27	0.40	0.00	0.25	0.60	0.00	0.26	0.10	0.00	0.52	0.40	0.00	0.51	0.40	0.00	0.53
i320-031	1.35	0.06	0.70	1.68	0.06	0.10	1.35	0.06	0.80	1.68	0.07	0.40	1.35	0.07	0.40	**0.64**	0.07
i320-032	3.61	0.06	0.90	4.08	0.06	0.80	**0.69**	0.06	0.90	**3.36**	0.07	0.90	3.61	0.07	0.60	**0.25**	0.07
i320-033	12.06	0.06	0.50	**7.91**	0.06	0.80	**3.47**	0.06	0.60	**7.98**	0.07	0.60	**3.14**	0.07	0.40	**3.76**	0.08
i320-034	0.00	0.06	0.80	0.00	0.06	0.70	0.00	0.06	0.90	0.00	0.07	0.60	0.00	0.07	0.80	0.00	0.08
i320-035	3.77	0.06	0.40	**0.00**	0.06	0.30	**0.34**	0.06	0.70	**0.34**	0.07	0.30	**0.00**	0.07	0.40	**0.00**	0.08
i320-041	7.38	0.19	0.70	**3.51**	0.17	0.70	**4.10**	0.17	0.50	**4.10**	0.24	0.40	7.38	0.26	0.50	7.38	0.28
i320-042	0.24	0.19	0.50	0.83	0.15	0.80	**0.00**	0.19	0.70	0.24	0.25	0.50	**0.00**	0.26	0.90	**0.00**	0.27
i320-043	0.23	0.19	0.90	0.75	0.19	0.80	0.75	0.19	0.50	0.75	0.24	0.30	**0.17**	0.26	0.50	0.23	0.28
i320-044	0.59	0.19	0.60	**0.00**	0.16	0.70	**0.00**	0.17	0.20	0.59	0.21	0.20	0.59	0.25	0.90	0.59	0.27
i320-045	0.00	0.19	0.90	0.00	0.20	0.60	0.00	0.16	0.80	0.00	0.26	0.60	0.00	0.26	0.90	10.14	0.27
i320-101	0.11	0.05	0.90	1.87	0.05	0.20	0.11	0.05	0.90	1.87	0.06	0.10	0.11	0.07	0.30	0.11	0.07
i320-102	3.44	0.05	0.50	**2.18**	0.05	0.20	**2.05**	0.05	0.30	**2.18**	0.06	0.30	**1.84**	0.07	0.30	**1.62**	0.07
i320-103	2.39	0.05	0.80	2.42	0.05	0.80	**2.28**	0.05	0.40	**0.46**	0.06	0.90	2.42	0.07	0.90	2.42	0.07
i320-104	5.35	0.05	0.90	**5.17**	0.05	0.30	**3.44**	0.05	0.90	**5.17**	0.06	0.50	**4.73**	0.07	0.60	**3.26**	0.07
i320-105	1.70	0.05	0.90	3.44	0.05	0.80	1.70	0.05	0.80	3.44	0.07	0.50	**1.62**	0.07	0.90	1.70	0.07
i320-111	8.71	0.10	0.70	**2.74**	0.10	0.90	**5.20**	0.10	0.30	**0.70**	0.12	0.10	**5.29**	0.12	0.10	**4.31**	0.13
i320-112	2.49	0.10	0.50	4.01	0.10	0.90	**1.02**	0.10	0.80	**1.50**	0.12	0.10	2.94	0.12	0.30	**0.71**	0.13
i320-113	2.26	0.10	0.70	**0.55**	0.10	0.90	**0.45**	0.10	0.80	5.11	0.12	0.80	2.26	0.12	0.30	**0.21**	0.13
i320-114	0.00	0.10	0.90	1.29	0.10	0.90	0.00	0.10	0.80	0.00	0.12	0.30	0.00	0.12	0.30	0.00	0.13
i320-115	7.36	0.10	0.80	**7.17**	0.10	0.90	**4.91**	0.10	0.90	10.59	0.13	0.20	**5.38**	0.13	0.90	**2.60**	0.13
i320-121	0.00	0.27	0.20	0.00	0.26	0.50	0.00	0.26	0.10	0.00	0.52	0.20	0.00	0.51	0.20	0.00	0.53
i320-122	0.00	0.27	0.20	0.00	0.25	0.50	0.00	0.26	0.10	0.00	0.52	0.20	0.00	0.51	0.20	0.00	0.53
i320-123	0.00	0.27	0.90	0.00	0.25	0.50	0.00	0.26	0.90	0.00	0.52	0.90	0.00	0.51	0.90	0.00	0.53
i320-124	0.00	0.27	0.20	0.00	0.25	0.50	0.00	0.26	0.10	0.00	0.52	0.20	0.00	0.51	0.20	0.00	0.53
i320-125	0.15	0.27	0.30	0.15	0.25	0.50	0.15	0.26	0.10	0.15	0.52	0.30	0.15	0.51	0.30	0.15	0.53
i320-131	8.72	0.06	0.60	**6.26**	0.06	0.80	**7.69**	0.06	0.60	**2.23**	0.08	0.60	**1.62**	0.08	0.60	**2.13**	0.08
i320-132	5.64	0.06	0.60	**2.20**	0.06	0.30	**0.00**	0.06	0.50	**0.06**	0.07	0.20	5.64	0.08	0.30	**4.08**	0.08
i320-133	4.27	0.06	0.90	**2.20**	0.06	0.20	**2.67**	0.07	0.80	**2.20**	0.08	0.90	**2.38**	0.08	0.80	**2.38**	0.08
i320-134	5.08	0.06	0.50	**3.19**	0.06	0.90	5.08	0.06	0.50	**3.34**	0.08	0.30	**1.99**	0.08	0.20	**3.28**	0.08
i320-135	3.43	0.06	0.50	**2.68**	0.06	0.60	3.48	0.06	0.90	**2.34**	0.08	0.90	**3.41**	0.08	0.30	3.89	0.08
i320-141	0.00	0.20	0.90	3.19	0.20	0.80	0.00	0.19	0.70	0.00	0.26	0.90	0.39	0.26	0.80	1.80	0.27
i320-142	0.00	0.20	0.60	3.87	0.16	0.60	0.00	0.16	0.70	0.00	0.26	0.60	0.00	0.26	0.80	1.85	0.27
i320-143	2.08	0.20	0.90	**0.00**	0.20	0.80	**0.00**	0.19	0.60	**0.00**	0.26	0.40	**0.00**	0.26	0.90	**1.71**	0.27
i320-144	0.00	0.20	0.80	0.03	0.19	0.90	0.00	0.20	0.40	0.00	0.25	0.70	0.00	0.26	0.80	0.03	0.27
i320-145	0.25	0.20	0.70	0.47	0.18	0.70	0.47	0.17	0.10	0.47	0.25	0.30	**0.00**	0.26	0.90	0.36	0.27

Table S23. (Continued) Results of the benchmark problem set I320 by using the ADH. Texts with a gray background are complete graph instances.

	without centrality		with centrality															
			degree			eigenvector			closeness			vertex betweenness			edge betweenness		
name	gap	time	α	gap	time	α	gap	time	α	gap	time	α	gap	time	α	gap	time
i320-201	3.67	0.07	0.90	4.15	0.07	0.70	**3.20**	0.07	0.90	**3.48**	0.08	0.80	**3.59**	0.09	0.90	3.67	0.08
i320-202	4.50	0.07	0.90	**2.97**	0.07	0.90	**2.79**	0.07	0.20	**2.21**	0.08	0.60	**2.74**	0.08	0.70	**2.96**	0.08
i320-203	4.11	0.07	0.40	**2.28**	0.07	0.20	**4.10**	0.07	0.70	**3.01**	0.08	0.80	5.05	0.09	0.90	**3.21**	0.09
i320-204	7.86	0.07	0.70	**3.04**	0.07	0.10	**6.73**	0.07	0.60	**3.16**	0.08	0.50	**2.16**	0.09	0.80	**1.70**	0.09
i320-205	3.83	0.07	0.80	**3.11**	0.07	0.50	**3.06**	0.07	0.80	**3.02**	0.08	0.40	**3.07**	0.08	0.40	**2.76**	0.09
i320-211	5.90	0.12	0.80	**3.92**	0.12	0.60	**4.20**	0.12	0.80	**5.03**	0.14	0.30	5.90	0.14	0.80	5.90	0.14
i320-212	3.89	0.12	0.90	4.26	0.12	0.70	**3.27**	0.12	0.90	4.04	0.14	0.30	**3.64**	0.14	0.20	**2.78**	0.14
i320-213	5.17	0.12	0.90	5.24	0.12	0.80	**2.49**	0.12	0.70	**4.11**	0.14	0.10	**4.62**	0.14	0.80	**3.02**	0.14
i320-214	5.11	0.12	0.80	6.13	0.12	0.70	**4.93**	0.12	0.60	5.21	0.14	0.20	**2.71**	0.14	0.80	**3.67**	0.14
i320-215	6.64	0.12	0.80	**3.12**	0.12	0.80	**3.57**	0.12	0.90	**1.56**	0.14	0.40	**2.63**	0.15	0.70	**1.76**	0.15
i320-221	0.31	0.29	0.20	**0.27**	0.27	0.60	**0.27**	0.28	0.10	**0.27**	0.54	0.20	**0.27**	0.53	0.20	**0.27**	0.54
i320-222	0.03	0.29	0.20	0.03	0.27	0.50	0.03	0.28	0.10	0.03	0.54	0.20	0.03	0.53	0.20	0.03	0.55
i320-223	0.21	0.29	0.20	0.21	0.27	0.60	0.21	0.28	0.10	0.21	0.54	0.20	0.21	0.53	0.20	0.21	0.55
i320-224	0.04	0.29	0.70	**0.00**	0.27	0.50	**0.00**	0.28	0.60	**0.00**	0.54	0.70	**0.00**	0.53	0.70	**0.00**	0.55
i320-225	0.15	0.29	0.20	0.15	0.27	0.50	0.15	0.28	0.10	0.15	0.54	0.20	0.15	0.53	0.20	0.15	0.55
i320-231	8.44	0.08	0.40	**4.25**	0.08	0.70	**4.79**	0.08	0.20	**3.33**	0.09	0.70	**5.54**	0.10	0.40	**4.78**	0.10
i320-232	2.74	0.08	0.70	5.27	0.08	0.80	4.67	0.08	0.90	3.70	0.09	0.90	3.72	0.10	0.80	4.64	0.10
i320-233	5.83	0.08	0.90	**5.13**	0.08	0.80	**2.83**	0.08	0.30	7.44	0.09	0.80	**3.96**	0.10	0.60	**3.95**	0.10
i320-234	5.75	0.08	0.20	**3.57**	0.08	0.80	**4.76**	0.08	0.10	**3.77**	0.09	0.50	5.99	0.10	0.70	5.88	0.10
i320-235	4.65	0.08	0.50	**3.66**	0.08	0.60	**3.04**	0.08	0.80	**2.62**	0.09	0.70	**4.54**	0.10	0.80	5.56	0.10
i320-241	2.33	0.21	0.60	**1.76**	0.18	0.60	**0.36**	0.18	0.90	**1.95**	0.27	0.20	**0.88**	0.27	0.90	**1.74**	0.29
i320-242	1.70	0.21	0.80	2.09	0.20	0.90	1.73	0.21	0.90	1.82	0.27	0.70	1.70	0.28	0.80	2.88	0.28
i320-243	0.31	0.21	0.60	1.24	0.18	0.90	0.31	0.21	0.90	0.31	0.27	0.70	0.31	0.28	0.80	1.60	0.29
i320-244	0.72	0.21	0.90	0.82	0.21	0.80	1.03	0.20	0.70	0.82	0.27	0.90	0.72	0.28	0.90	0.90	0.29
i320-245	1.50	0.21	0.70	**0.14**	0.19	0.60	**0.18**	0.18	0.50	1.56	0.26	0.90	**0.00**	0.28	0.90	1.77	0.29
i320-301	3.76	0.18	0.30	**2.75**	0.18	0.70	**2.69**	0.18	0.90	**3.00**	0.20	0.60	**1.23**	0.20	0.20	**2.03**	0.20
i320-302	6.15	0.19	0.90	**4.52**	0.18	0.10	**3.27**	0.19	0.60	**5.07**	0.20	0.60	**1.90**	0.20	0.70	**2.27**	0.20
i320-303	2.84	0.19	0.60	**1.65**	0.19	0.90	**2.77**	0.19	0.70	**2.61**	0.20	0.30	**2.35**	0.20	0.90	3.32	0.20
i320-304	5.10	0.19	0.20	**1.55**	0.19	0.30	**2.17**	0.19	0.50	**2.34**	0.20	0.20	**2.25**	0.21	0.20	**2.53**	0.20
i320-305	3.51	0.19	0.90	**2.23**	0.18	0.90	**1.39**	0.19	0.50	**2.16**	0.20	0.20	**1.54**	0.20	0.10	**1.95**	0.20
i320-311	2.76	0.23	0.80	3.74	0.24	0.70	3.78	0.24	0.90	4.42	0.26	0.20	3.42	0.26	0.70	3.17	0.26
i320-312	2.64	0.24	0.90	4.25	0.24	0.70	**2.60**	0.24	0.90	4.07	0.26	0.40	3.01	0.26	0.40	**2.09**	0.26
i320-313	5.21	0.23	0.90	**3.42**	0.23	0.80	**3.08**	0.24	0.90	**4.49**	0.26	0.10	**2.54**	0.26	0.20	**2.86**	0.26
i320-314	1.85	0.24	0.90	**1.72**	0.24	0.80	3.55	0.24	0.90	2.30	0.26	0.30	1.85	0.26	0.80	**1.81**	0.26
i320-315	2.99	0.24	0.90	3.08	0.24	0.80	**2.57**	0.24	0.70	**2.83**	0.26	0.20	2.99	0.26	0.70	**2.87**	0.26
i320-321	0.38	0.39	0.90	0.38	0.38	0.80	0.38	0.40	0.80	0.38	0.64	0.90	0.38	0.64	0.90	0.38	0.65
i320-322	0.34	0.39	0.90	**0.14**	0.38	0.90	**0.14**	0.39	0.70	**0.14**	0.64	0.90	**0.14**	0.63	0.90	**0.14**	0.65
i320-323	0.20	0.39	0.80	0.20	0.38	0.80	0.20	0.40	0.70	0.20	0.64	0.80	0.20	0.64	0.80	0.20	0.65
i320-324	0.31	0.39	0.90	**0.27**	0.38	0.90	**0.27**	0.40	0.80	**0.27**	0.64	0.90	**0.27**	0.64	0.90	**0.27**	0.65
i320-325	0.45	0.39	0.90	0.45	0.38	0.90	0.45	0.40	0.80	0.45	0.64	0.90	0.45	0.64	0.90	0.45	0.65
i320-331	8.56	0.20	0.90	**5.08**	0.19	0.40	**4.41**	0.19	0.90	**6.34**	0.21	0.10	**4.25**	0.22	0.50	**3.77**	0.22
i320-332	5.85	0.20	0.60	**4.64**	0.19	0.50	**3.05**	0.20	0.60	**3.19**	0.21	0.60	**3.84**	0.21	0.40	**3.17**	0.22
i320-333	5.30	0.20	0.60	**3.95**	0.19	0.70	**4.12**	0.20	0.60	**4.40**	0.21	0.20	**3.29**	0.21	0.80	**3.89**	0.21
i320-334	5.67	0.20	0.90	6.49	0.20	0.90	**4.38**	0.20	0.30	6.04	0.21	0.40	**3.13**	0.21	0.90	**3.39**	0.22
i320-335	4.14	0.20	0.70	**2.97**	0.20	0.70	**2.91**	0.20	0.70	**2.96**	0.21	0.20	**3.30**	0.22	0.30	**3.81**	0.22
i320-341	0.62	0.32	0.90	0.87	0.32	0.80	0.77	0.32	0.80	0.91	0.37	0.80	**0.34**	0.38	0.80	0.64	0.39
i320-342	0.46	0.32	0.90	**0.06**	0.33	0.80	**0.07**	0.31	0.90	0.68	0.38	0.20	**0.30**	0.38	0.90	0.62	0.39
i320-343	1.07	0.32	0.80	1.20	0.31	0.80	**0.96**	0.32	0.70	**0.60**	0.38	0.60	**0.82**	0.39	0.90	**1.04**	0.39
i320-344	0.23	0.31	0.90	1.17	0.32	0.90	0.29	0.32	0.90	0.52	0.38	0.70	0.23	0.38	0.90	1.14	0.39
i320-345	0.63	0.32	0.90	0.77	0.32	0.80	0.63	0.31	0.90	0.91	0.38	0.40	**0.09**	0.38	0.90	0.67	0.39
average	3.02	0.17	-	**2.30** (-0.72)	0.17	-	**1.93** (-1.09)	0.17	-	**2.14** (-0.88)	0.24	-	**1.84** (-1.18)	0.24	-	**1.91** (-1.11)	0.25

Table S24. Results of the benchmark problem set I640 by using the ADH. Texts with a gray background are complete graph instances.

	without centrality		with centrality														
			degree			eigenvector			closeness			vertex betweenness			edge betweenness		
name	gap	time	α	gap	time	α	gap	time	α	gap	time	α	gap	time	α	gap	time
i640-001	0.00	0.38	0.60	0.00	0.38	0.10	6.67	0.36	0.60	2.18	0.43	0.50	2.18	0.50	0.50	2.18	0.51
i640-002	11.29	0.34	0.40	**0.53**	0.37	0.70	**3.21**	0.42	0.60	**0.53**	0.51	0.30	**0.53**	0.50	0.00	**0.53**	0.56
i640-003	5.61	0.41	0.50	5.61	0.41	0.50	5.73	0.37	0.50	5.70	0.50	0.70	5.61	0.53	0.80	5.61	0.44
i640-004	9.12	0.39	0.10	**1.98**	0.43	0.10	**3.75**	0.41	0.00	**1.98**	0.51	0.10	**1.98**	0.58	0.20	**2.00**	0.56
i640-005	9.11	0.37	0.70	**4.37**	0.38	0.10	**6.57**	0.35	0.60	**1.82**	0.44	0.30	**2.55**	0.49	0.10	**0.00**	0.54
i640-011	14.09	0.77	0.30	**10.91**	0.74	0.20	**11.96**	0.83	0.90	**11.20**	1.09	0.30	**10.03**	0.98	0.80	**13.84**	1.07
i640-012	6.98	0.90	0.90	6.98	0.90	0.90	6.98	0.81	0.90	**3.65**	1.06	0.20	**3.89**	1.09	0.50	**3.89**	1.12
i640-013	6.63	0.74	0.40	**4.25**	0.70	0.60	**3.50**	0.74	0.90	**6.29**	0.99	0.50	**6.38**	1.05	0.40	**6.38**	1.07
i640-014	8.75	0.81	0.90	8.75	0.81	0.70	**4.93**	0.81	0.80	**4.33**	1.06	0.30	**4.51**	1.15	0.20	**0.37**	1.14
i640-015	1.02	0.77	0.80	**0.09**	0.72	0.90	**0.77**	0.92	0.10	**0.09**	1.07	0.30	1.02	1.13	0.30	1.02	0.99
i640-021	0.00	2.67	0.40	0.00	2.58	0.60	0.00	2.77	0.10	0.11	8.28	0.40	0.00	7.60	0.40	0.00	7.89
i640-022	0.06	2.46	0.40	0.06	2.51	0.60	0.06	2.64	0.10	0.06	7.74	0.40	0.06	7.29	0.40	0.06	7.45
i640-023	0.06	2.70	0.40	0.06	2.78	0.60	0.06	2.82	0.10	**0.00**	8.32	0.40	0.06	8.05	0.40	0.06	8.11
i640-024	0.06	2.52	0.40	0.06	2.50	0.60	0.11	2.57	0.20	**0.00**	8.27	0.40	0.06	8.07	0.40	0.06	8.14
i640-025	0.63	2.83	0.50	**0.00**	2.83	0.60	0.63	2.79	0.10	**0.00**	8.30	0.50	**0.00**	7.94	0.50	**0.00**	8.10
i640-031	0.79	0.52	0.90	0.79	0.52	0.30	0.79	0.57	0.70	3.23	0.62	0.60	**0.00**	0.67	0.70	**0.46**	0.65
i640-032	2.17	0.51	0.90	2.17	0.51	0.40	2.32	0.50	0.90	2.32	0.60	0.40	2.17	0.66	0.80	2.17	0.64
i640-033	9.14	0.51	0.00	**0.83**	0.55	0.10	**3.44**	0.61	0.80	**3.47**	0.63	0.40	**0.37**	0.65	0.60	**0.43**	0.64
i640-034	4.27	0.49	0.90	4.27	0.49	0.80	**3.86**	0.44	0.90	4.44	0.63	0.40	**0.30**	0.61	0.50	**3.79**	0.63
i640-035	3.22	0.49	0.70	**0.00**	0.49	0.70	**0.00**	0.54	0.90	**0.33**	0.54	0.90	**0.00**	0.63	0.80	**2.55**	0.53
i640-041	9.33	2.06	0.70	**8.91**	1.74	0.90	**4.01**	1.99	0.80	**4.01**	3.27	0.20	**3.80**	3.25	0.80	**6.91**	3.50
i640-042	7.19	2.08	0.60	**3.10**	1.73	0.60	**2.90**	1.72	0.10	**2.74**	3.16	0.20	**1.91**	3.22	0.50	**4.29**	3.63
i640-043	6.06	2.10	0.70	**2.43**	1.85	0.70	**2.28**	1.83	0.60	6.06	3.24	0.10	**0.57**	3.12	0.70	**0.00**	3.56
i640-044	2.32	2.13	0.90	2.32	2.13	0.90	2.32	2.10	0.50	2.32	3.20	0.90	**0.62**	3.29	0.90	**0.62**	3.50
i640-045	0.00	2.00	0.80	0.00	1.94	0.80	0.00	1.94	0.10	0.00	3.19	0.10	0.00	3.15	0.90	0.00	3.54
i640-101	8.10	0.43	0.50	**2.76**	0.45	0.70	**3.54**	0.50	0.90	**5.64**	0.56	0.90	**5.47**	0.58	0.70	**5.64**	0.59
i640-102	8.51	0.43	0.30	**3.51**	0.45	0.20	**7.88**	0.47	0.40	**2.89**	0.55	0.40	**4.19**	0.57	0.90	**3.86**	0.58
i640-103	6.03	0.46	0.70	**5.67**	0.46	0.90	**4.35**	0.49	0.80	7.91	0.57	0.60	**3.24**	0.58	0.60	**2.09**	0.59
i640-104	5.11	0.45	0.60	**4.28**	0.45	0.30	**4.28**	0.52	0.80	**3.74**	0.56	0.40	**3.08**	0.57	0.50	**3.89**	0.58
i640-105	4.82	0.41	0.80	**4.21**	0.43	0.20	5.91	0.48	0.70	**3.49**	0.53	0.40	**3.92**	0.55	0.60	**2.32**	0.58
i640-111	6.84	0.92	0.80	**1.95**	0.89	0.60	**1.82**	0.92	0.80	**3.71**	1.17	0.10	**2.93**	1.19	0.70	**3.23**	1.19
i640-112	8.91	0.93	0.60	**8.57**	0.91	0.70	**3.78**	0.96	0.90	**5.01**	1.18	0.60	**4.92**	1.18	0.90	**6.77**	1.20
i640-113	6.16	0.93	0.60	**4.67**	0.92	0.90	**5.52**	0.98	0.90	**4.19**	1.16	0.10	7.06	1.19	0.40	**3.42**	1.16
i640-114	5.04	0.94	0.90	5.04	0.95	0.80	5.56	0.96	0.90	**3.77**	1.14	0.10	**2.71**	1.14	0.20	**2.84**	1.16
i640-115	5.15	0.91	0.90	5.15	0.91	0.80	5.28	0.95	0.60	**5.12**	1.04	0.20	6.22	1.18	0.20	**3.52**	1.20
i640-121	0.00	2.84	0.20	0.00	2.78	0.50	0.00	2.98	0.10	0.00	8.14	0.20	0.00	7.79	0.20	0.00	7.91
i640-122	0.00	2.71	0.20	0.00	2.77	0.50	0.00	2.63	0.10	0.00	7.80	0.20	0.00	7.40	0.20	0.00	7.73
i640-123	0.00	2.58	0.80	0.00	2.54	0.50	0.00	2.68	0.80	0.00	7.74	0.80	0.00	7.44	0.80	0.00	7.47
i640-124	0.04	2.74	0.20	0.04	2.79	0.50	0.04	2.80	0.10	0.04	7.94	0.20	0.04	7.65	0.20	0.04	7.71
i640-125	0.00	2.70	0.20	0.00	2.77	0.50	0.00	2.84	0.10	0.00	8.07	0.20	0.00	7.74	0.20	0.00	7.63
i640-131	4.99	0.54	0.90	4.99	0.55	0.70	**2.42**	0.58	0.90	**3.61**	0.69	0.80	**2.42**	0.69	0.20	**3.79**	0.72
i640-132	10.68	0.56	0.80	**7.59**	0.56	0.60	**7.86**	0.60	0.10	**5.43**	0.66	0.50	**7.36**	0.71	0.70	**5.80**	0.70
i640-133	6.07	0.53	0.80	**4.64**	0.54	0.20	**2.22**	0.54	0.90	**3.22**	0.67	0.80	9.51	0.72	0.80	**4.36**	0.72
i640-134	5.03	0.57	0.40	**1.59**	0.55	0.70	**3.57**	0.60	0.60	**2.58**	0.68	0.70	**5.02**	0.71	0.90	**3.93**	0.71
i640-135	5.54	0.52	0.90	5.54	0.52	0.70	**2.55**	0.54	0.70	5.89	0.68	0.60	**5.44**	0.69	0.30	**4.33**	0.71
i640-141	1.62	2.11	0.80	**1.02**	2.04	0.70	**0.73**	1.85	0.80	**0.23**	3.27	0.80	**0.23**	3.27	0.80	2.56	3.50
i640-142	1.87	2.14	0.80	**1.68**	2.03	0.80	**0.31**	2.06	0.30	**0.73**	3.09	0.50	**1.41**	3.13	0.90	**0.81**	3.26
i640-143	1.81	2.04	0.90	1.81	2.04	0.90	1.81	2.08	0.90	**0.00**	3.21	0.40	**0.33**	3.15	0.90	2.79	3.51
i640-144	3.38	2.14	0.70	**1.00**	1.89	0.70	**0.90**	1.91	0.50	**0.94**	3.27	0.10	**0.90**	3.16	0.90	3.38	3.51
i640-145	1.90	2.07	0.90	1.90	2.07	0.60	**1.42**	1.78	0.90	**1.32**	3.17	0.30	**1.25**	3.27	0.90	2.36	3.53

Table S24. (Continued) Results of the benchmark problem set I640 by using the ADH. Texts with a gray background are complete graph instances.

	without centrality		with centrality														
			degree			eigenvector			closeness			vertex betweenness			edge betweenness		
name	gap	time	α	gap	time	α	gap	time	α	gap	time	α	gap	time	α	gap	time
i640-201	8.32	0.57	0.50	**4.64**	0.60	0.70	**3.43**	0.60	0.40	**5.04**	0.68	0.20	**0.71**	0.73	0.30	**2.92**	0.71
i640-202	2.89	0.57	0.90	2.89	0.57	0.60	**2.42**	0.61	0.70	6.36	0.67	0.40	3.84	0.73	0.80	3.63	0.72
i640-203	4.60	0.57	0.90	4.60	0.57	0.50	5.55	0.60	0.60	**2.69**	0.68	0.10	**2.73**	0.71	0.10	**2.54**	0.73
i640-204	6.61	0.57	0.70	**2.79**	0.57	0.10	**4.06**	0.64	0.20	6.99	0.68	0.60	**5.09**	0.70	0.40	**5.67**	0.69
i640-205	5.58	0.54	0.80	**3.29**	0.54	0.70	**4.02**	0.62	0.80	**4.25**	0.65	0.90	**4.25**	0.69	0.10	**4.07**	0.73
i640-211	10.05	1.00	0.80	**6.74**	0.98	0.60	**6.17**	1.09	0.70	**6.43**	1.25	0.10	**5.63**	1.27	0.50	**3.28**	1.29
i640-212	5.72	1.08	0.80	**5.60**	1.05	0.70	**4.76**	1.08	0.80	**4.81**	1.26	0.30	**5.15**	1.31	0.40	**5.08**	1.31
i640-213	7.50	1.06	0.70	**6.82**	1.05	0.70	**6.14**	1.06	0.50	**5.10**	1.23	0.10	**4.28**	1.32	0.20	**4.10**	1.30
i640-214	6.85	1.03	0.80	**5.30**	1.04	0.80	**5.35**	1.07	0.80	**6.23**	1.22	0.10	**6.09**	1.26	0.20	**4.99**	1.12
i640-215	7.51	1.07	0.60	**3.66**	1.05	0.60	**3.96**	1.09	0.90	**3.78**	1.29	0.10	**5.94**	1.29	0.30	**4.26**	1.30
i640-221	0.56	2.95	0.10	0.56	2.94	0.40	0.56	2.96	0.10	0.56	8.20	0.10	0.56	7.75	0.10	0.56	7.88
i640-222	0.76	2.85	0.10	0.76	2.92	0.90	0.76	2.94	0.10	0.76	8.23	0.10	0.76	7.85	0.10	0.76	7.91
i640-223	0.61	2.89	0.10	**0.48**	2.84	0.40	0.61	2.72	0.10	**0.48**	8.01	0.10	**0.48**	7.53	0.10	**0.48**	7.64
i640-224	0.85	2.85	0.10	**0.74**	2.84	0.50	0.85	2.93	0.10	**0.74**	7.86	0.10	**0.74**	7.56	0.10	**0.74**	7.47
i640-225	0.54	2.55	0.10	0.54	2.57	0.40	0.54	2.81	0.10	0.54	7.95	0.10	0.54	7.66	0.10	0.54	7.54
i640-231	8.63	0.58	0.80	**7.57**	0.58	0.40	**6.39**	0.69	0.60	**6.79**	0.70	0.40	**4.60**	0.79	0.80	**6.02**	0.80
i640-232	4.61	0.61	0.90	4.61	0.61	0.40	**3.94**	0.65	0.90	**4.05**	0.70	0.90	5.01	0.77	0.70	**4.33**	0.79
i640-233	6.20	0.67	0.50	**5.01**	0.66	0.80	**5.06**	0.70	0.10	**5.97**	0.76	0.70	**4.01**	0.81	0.40	**4.97**	0.82
i640-234	6.28	0.54	0.70	**4.58**	0.55	0.90	**4.12**	0.54	0.30	**4.89**	0.76	0.80	6.95	0.83	0.60	**6.14**	0.82
i640-235	4.65	0.68	0.90	4.65	0.68	0.60	**2.68**	0.73	0.80	6.06	0.77	0.30	5.77	0.76	0.60	**3.88**	0.70
i640-241	0.95	2.18	0.90	0.95	2.19	0.90	**0.91**	1.97	0.20	0.99	3.39	0.20	**0.49**	3.28	0.90	1.38	3.58
i640-242	1.14	2.04	0.90	1.14	2.04	0.70	**0.80**	1.87	0.30	**1.11**	3.07	0.90	**0.33**	3.17	0.90	**0.86**	3.38
i640-243	0.43	2.16	0.90	0.43	2.16	0.70	0.44	2.03	0.20	0.43	3.27	0.30	**0.41**	3.30	0.80	1.61	3.15
i640-244	0.65	2.27	0.80	**0.49**	2.18	0.90	**0.61**	2.08	0.10	**0.41**	3.18	0.70	**0.40**	3.41	0.90	1.86	3.60
i640-245	0.77	2.25	0.90	0.77	2.25	0.90	**0.36**	2.24	0.30	1.31	3.36	0.30	**0.47**	3.26	0.90	1.30	3.51
i640-301	3.32	2.03	0.90	3.32	2.03	0.40	**2.50**	2.05	0.80	3.61	1.92	0.70	**1.97**	2.13	0.80	**2.66**	1.94
i640-302	3.52	1.78	0.90	3.52	1.79	0.10	**3.17**	1.88	0.60	3.83	1.95	0.30	**2.47**	2.04	0.90	**2.64**	2.10
i640-303	2.42	1.90	0.50	**2.34**	1.88	0.10	**2.37**	1.94	0.20	2.65	1.97	0.40	**1.41**	2.09	0.10	**2.21**	2.03
i640-304	4.37	1.96	0.50	**4.22**	1.98	0.90	**2.93**	2.01	0.80	**4.02**	2.15	0.20	**2.84**	2.20	0.80	**3.37**	2.16
i640-305	4.89	2.01	0.50	**3.54**	2.07	0.30	**3.83**	2.11	0.80	**4.09**	2.16	0.20	**3.62**	2.19	0.40	**4.01**	2.24
i640-311	2.82	2.51	0.70	**2.60**	2.49	0.90	2.84	2.49	0.80	**2.80**	2.36	0.20	**2.05**	2.71	0.90	**1.97**	2.72
i640-312	3.96	2.55	0.70	**3.59**	2.51	0.70	**3.35**	2.61	0.80	**3.52**	2.75	0.10	**3.63**	2.78	0.40	**2.87**	2.78
i640-313	2.22	2.48	0.90	2.22	2.48	0.90	2.90	2.33	0.80	2.51	2.78	0.10	2.30	2.81	0.50	**2.21**	2.75
i640-314	2.32	2.56	0.90	2.32	2.56	0.90	**1.67**	2.59	0.70	**1.44**	2.75	0.10	**1.56**	2.78	0.20	**1.69**	2.75
i640-315	2.65	2.23	0.90	2.65	2.23	0.80	3.08	2.61	0.80	2.74	2.79	0.30	**1.79**	2.74	0.70	**1.74**	2.78
i640-321	0.40	4.08	0.90	0.40	4.08	0.90	0.40	4.11	0.90	**0.14**	9.24	0.90	0.40	9.05	0.90	0.40	9.14
i640-322	0.57	4.09	0.90	0.57	4.10	0.90	0.57	4.15	0.90	**0.29**	9.13	0.90	0.57	8.44	0.90	0.57	8.63
i640-323	0.56	3.26	0.90	0.56	3.26	0.90	0.56	4.13	0.80	**0.50**	8.89	0.90	0.56	8.54	0.90	0.56	8.91
i640-324	0.31	3.34	0.90	0.31	3.34	0.90	0.31	4.13	0.90	0.31	8.35	0.90	0.31	8.68	0.90	0.31	8.01
i640-325	0.19	4.03	0.90	0.19	4.03	0.90	0.19	4.10	0.90	**0.16**	8.61	0.90	0.19	8.23	0.90	0.19	8.73
i640-331	4.49	1.82	0.80	**2.95**	1.84	0.90	**3.56**	2.01	0.80	**3.89**	2.10	0.20	4.62	2.26	0.20	**3.56**	2.33
i640-332	5.53	1.89	0.80	**4.43**	1.93	0.60	**2.38**	1.76	0.80	**5.03**	2.01	0.10	**3.03**	2.30	0.20	**3.45**	2.24
i640-333	5.77	1.71	0.80	**4.21**	1.70	0.90	**4.83**	1.75	0.30	5.92	2.29	0.50	**4.13**	2.19	0.70	**3.44**	2.31
i640-334	3.76	1.68	0.90	3.76	1.68	0.40	**2.30**	2.04	0.90	**2.47**	2.22	0.30	**3.07**	2.28	0.80	**3.22**	2.28
i640-335	5.78	1.90	0.50	**5.38**	1.96	0.50	**4.35**	2.15	0.80	**4.79**	2.25	0.40	**4.69**	2.31	0.50	**5.04**	2.17
i640-341	0.33	3.14	0.90	0.33	3.14	0.90	**0.27**	3.06	0.60	0.34	4.25	0.50	**0.19**	4.44	0.90	0.61	4.36
i640-342	0.70	3.52	0.90	0.70	3.52	0.90	**0.22**	3.59	0.10	**0.23**	4.10	0.40	**0.16**	4.04	0.90	**0.43**	4.87
i640-343	0.42	3.51	0.90	0.42	3.52	0.90	**0.33**	3.67	0.80	**0.22**	4.34	0.20	**0.30**	3.85	0.90	0.55	4.82
i640-344	0.29	3.49	0.90	0.29	3.49	0.80	0.48	3.59	0.80	0.43	4.67	0.30	**0.24**	4.49	0.90	0.98	4.63
i640-345	0.69	2.92	0.80	**0.54**	2.84	0.80	**0.34**	3.30	0.80	**0.29**	3.90	0.50	**0.13**	4.52	0.90	**0.63**	4.54
average	3.91	1.68	-	**2.81** (-1.10)	1.67	-	**2.73** (-1.18)	1.72	-	**2.79** (-1.12)	3.05	-	**2.48** (-1.43)	3.01	-	**2.54** (-1.37)	3.08
